# Health and incarceration research in Australia: a scoping review

**DOI:** 10.1016/j.lanwpc.2025.101500

**Published:** 2025-03-18

**Authors:** Sarah A. Pellicano, Lindsay A. Pearce, Alexander C. Campbell, Rebecca Shuttleworth, Stuart A. Kinner

**Affiliations:** a Justice Health Group, enAble Institute, Curtin University, Building 400 Kent Street, Bentley, WA, 6102, Australia; b Justice Health Group, Centre for Adolescent Health, Murdoch Children's Research Institute & Royal Children's Hospital, VIC, Australia; c Centre for Epidemiology and Biostatistics, Melbourne School of Population and Global Health, University of Melbourne, VIC, Australia; d Griffith Criminology Institute, Griffith University, QLD, Australia; e Centre for Mental Health and Community Wellbeing, Melbourne School of Population and Global Health, University of Melbourne, VIC, Australia

**Keywords:** Health inequities, Public health, Justice health research, Youth detention, Prison

## Abstract

**Background:**

People who experience incarceration often have complex healthcare needs and poorer health than the general population. Australia has the eighth-largest custodial population in the Western Pacific. Understanding the breadth and quality of research on this population's health is crucial for advancing health equity both in Australia and across the region. This scoping review synthesised health research involving people in contact with the criminal justice system in Australia.

**Methods:**

We searched eight databases for primary, peer-reviewed research reporting on the health of people incarcerated or previously incarcerated in Australian prisons or youth detention settings.

**Findings:**

Our search identified 11,731 unique records, and 508 met the inclusion criteria. Over half (51%) were published between 2015 and 2024. Relatively few studies provided evidence on cognitive disabilities (16%), non-communicable diseases (14%), or sexual and reproductive health (6%). Few focused on youth detention (15%) or post-release health (24%). Only 27 studies (5%) focused exclusively on the health of First Nations Australians. Most studies (86%) came from Australia's most populous states—New South Wales, Queensland, and Victoria—which account for 68% of people incarcerated each year, and 58% of incarcerated First Nations peoples.

**Interpretation:**

Despite considerable growth in the number of peer-reviewed studies on the health of people who experience incarceration, critical health issues, key populations, and Australian jurisdictions with the highest incarceration rates require urgent attention. Further high-quality research is needed to fill these evidence gaps and translate research into evidence-based strategies that address the complex and diverse health needs of justice-involved people.

**Funding:**

SP was supported by a Australian Government Research Training Program Scholarship.

**Research in context**

**Evidence before this study**

We searched PROSPERO, the Cochrane Database of Systematic Reviews, and the Joanna Briggs Institute (JBI) of Evidence Synthesis for scoping and systematic reviews, published in English from inception to March 3, 2024. Search terms included variants and combinations relating to incarceration settings (i.e., prison, youth detention), health, and Australia. We did not identify any reviews synthesising the evidence involving people incarcerated or previously incarcerated in Australian custodial settings.

**Added value of this study**

In this review, we present the first comprehensive synthesis of research on the health of people in contact with the Australian criminal justice system. We identified few studies reporting on non-communicable diseases, cognitive disabilities, or sexual and reproductive health, despite evidence that these conditions place a substantial health burden on people who experience incarceration. While 324 studies (64%) identified the proportion of First Nations participants in their samples, only 27 studies (5%) focused exclusively on First Nations peoples. Women, children and adolescents, and people of diverse genders and sexualities in custodial settings also remain under-studied. Additionally, only 18% of studies came from five of Australia's eight jurisdictions, which together account for 23% of the Australian population, 32% of people in custody, and 42% of incarcerated First Nations peoples, and collectively have an incarceration rate 55% higher than the national average.

**Implications of all the available evidence**

Australia has a substantial, wide-ranging, and rapidly growing body of justice health research. Despite this, the diverse and complex health needs of people who experience incarceration in Australia are yet to be fully understood. Important evidence gaps remain. Coordinated and ongoing collaboration between researchers, policymakers, service providers, and people with lived experience of incarceration is required to better characterise, prevent, and effectively respond to the health challenges faced by people in contact with the criminal justice system. Prioritising population-level research that identifies the diversity of health-related needs across sub-groups and jurisdictions will further inform localised, targeted interventions and policies that can address the health and social inequities experienced by people in contact with the criminal justice system.

## Introduction

Incarceration is widely recognised as a structural determinant of health inequity.^1^, ^2^, ^3^, ^4^ While the World Health Organization (WHO) Regional Office for Europe has a longstanding focus on prison health through its Health in Prisons Programme,^5^ the WHO Regional Office for the Western Pacific is yet to recognise incarceration as a driver of health inequity.^6^ With over 2.3 million people incarcerated in the Western Pacific region, constituting 21% of the global prison population,^7^ this demands urgent attention. Rising incarceration rates in the region significantly constrain prison and public health systems to provide care proportionate to the high health needs of this population.^3^ Since 2000, Australia's incarceration rate has grown by 38%—from 114 to 157 per 100,000—surpassing increases in New Zealand (17%) and China (7%).^7^ In contrast, South Korea and Japan have reduced their incarceration rates by 24% and 31%, respectively.^7^ As one of the region's wealthiest countries, Australia has an opportunity to lead in providing equitable, high-quality healthcare for incarcerated people, supporting regional and global efforts towards achieving the Sustainable Development Goals (SDGs), particularly SDG 10 (reduced inequalities).^8^^,^^9^ The health and well-being of incarcerated people are integral to meeting this target in the Western Pacific region; national and sub-national data are crucial to tracking progress towards these goals.^10^

People who experience incarceration often have poorer health than the general population, including elevated rates of mental illness, cognitive disability, chronic and infectious disease, and substance use disorders.^11^^,^^12^ These health challenges are often compounded by multiple, intersecting experiences of social disadvantage—such as poverty, unstable housing, trauma, racism, and unemployment—and structural and systemic inequalities.^12^^,^^13^ Such adversities not only increase the risk of poor health but also contribute to cycles of recidivism and reincarceration.^14^, ^15^, ^16^ The complex health needs of people who experience incarceration warrant further investigation both within custodial settings and after release.^17^^,^^18^ Exposure to incarceration can intensify pre-existing health issues, socioeconomic disadvantage, and risk-taking behaviours.^13^^,^^19^ Poor custodial conditions (e.g., overcrowding) within correctional environments exacerbate associated health issues and present barriers to providing appropriate healthcare.^10^ Access to healthcare after release from custody may be impeded by challenges securing stable housing, transportation, employment, and welfare payments.^20^^,^^21^ Thus, the structural and systemic drivers that hinder equitable access to basic needs in the community perpetuate a cycle of poor health, vulnerability, and disadvantage.^13^

The United Nations Standard Minimum Rules for the Treatment of Prisoners (Nelson Mandela Rules) require that adults experiencing incarceration receive the same standards and accessibility to health services available in the community.^22^ Although the international standard regarding healthcare in youth detention is less clear,^23^ children and adolescents in criminal justice detention retain the right to the highest attainable standard of health. Given the concentration of complex health needs in custodial settings, additional investment in custodial healthcare is required to achieve equivalence.^11^^,^^24^ The rapid churn of people moving between custodial settings and communities highlights the need for a public health approach to addressing the health of those in contact with the criminal justice system.^10^^,^^25^ Accordingly, the WHO recommends the inclusion of ‘prison health in all policies’.^25^, ^26^, ^27^, ^28^ Public health strategies that facilitate access to appropriate health services and support continuity of care for this population are fundamental to improving the health of communities and meeting the SDGs.^8^^,^^9^

Despite growing global evidence regarding the health of incarcerated populations, global prevalence estimates and cross-national comparisons can overshadow complexity and heterogeneity at the national and sub-national levels^10^—as highlighted in reviews from the United States,^29^, ^30^, ^31^ Canada,^32^ and the United Kingdom.^33^ In Australia, young people aged 10–17 are primarily held in youth detention centres, while those aged 18 or over are incarcerated in adult prisons.^34^ Incarceration rates, sex ratios, and the proportion of First Nations Australians in custody vary markedly between jurisdictions.^35^^,^^36^ Despite an overall decline in the number of young people in detention, punitive, ‘tough-on-crime’ policies in certain jurisdictions have coincided with increases^37^—in the Northern Territory, the rate of young people in detention rose from 6.6 to 22.2 per 10,000 between 2020 and 2024.^35^ Over the same period, the adult prison population grew by 8%, with Western Australia recording the highest over-representation of First Nations adults in custody, at 4364 per 100,000.^36^

As a federated state, Australia's eight State and Territory governments are responsible for providing healthcare in custodial settings and independently legislating, funding, and overseeing their criminal justice systems, including healthcare in those systems.^19^^,^^38^ Understanding the Australian evidence on the health needs of people who experience incarceration, and what has been done to address these needs, is fundamental to providing equivalent custodial healthcare and coordinated throughcare; developing effective, targeted responses; and ensuring that incarceration serves to mitigate rather than compound health inequities.^39^^,^^40^ These insights can also inform efforts to reduce health inequities across the Western Pacific.

We conducted a scoping review to synthesise and describe the nature and extent of health research involving people incarcerated or previously incarcerated in Australian prisons or youth detention. This review aimed to identify research gaps, methodological limitations, strengths, and policy and practice-relevant research priorities.

## Methods

### Protocol registration

This scoping review was conducted in accordance with the Preferred Reporting Items for Systematic Reviews and Meta-Analyses Extension for Scoping Reviews (PRISMA-ScR).^41^ The protocol was registered prospectively with Open Science Framework (https://osf.io/q4rjm/).

### Search strategy and selection criteria

We searched eight electronic databases: MEDLINE, EMBASE, Cochrane Library, CINAHL Complete, PsycINFO, Global Health, ProQuest Central, and Scopus. Our search strategy included terms relating to incarceration settings (e.g., prison, youth detention), health, and Australian jurisdictions (Table S1). We searched all databases from inception to July 15, 2023, and did a rapid update on March 3, 2024. We reviewed the reference lists of included studies, relevant reviews, and grey literature to locate additional eligible studies, and contacted experts in the field to identify in-press publications.

We included primary, peer-reviewed studies examining health conditions and/or health-related outcomes (e.g., violence-related injury or death) in people incarcerated or previously incarcerated in Australian prisons or youth detention (Table S2). We did not include studies examining health-related knowledge, perceptions of health services, non-diagnostic markers of cognitive impairment or personality, or studies of risky health behaviours that did not report associated health outcomes. Studies were categorised and considered within nine *a priori* health domains:

1) Mental health status (including self-harm and suicidal behaviour);
2) Cognitive disabilities (including neurodevelopmental conditions);
3) Substance dependence and substance-related harm;
4) Bloodborne viruses and other communicable diseases (including human immunodeficiency virus; HIV);
5) Non-communicable diseases;
6) Physical health status (including exercise and obesity);
7) Sexual and reproductive health (including sexually transmitted infections; STIs);
8) Violence victimisation and violence-related injury; and
9) Health service use.

### Study selection

We uploaded publications retrieved from the database searches to EndNote (v20.6) referencing software and removed duplicates based on author name, journal, and year.^42^ We uploaded the list of de-duplicated publications to Rayyan software for further de-duplication and screening.^43^ Two reviewers (SP, ME, or JR) independently screened 10% of titles, abstracts, and full texts to measure interrater reliability. Kappa scores indicated adequate agreement for both title/abstract screening (*κ* = 0.72, *SE* = 0.04, 95% CI [0.64, 0.79]) and full-text screening (*κ* = 0.60, *SE* = 0.15, 95% CI [0.32, 0.89]). We resolved disagreements through discussion among the reviewers. One reviewer (SP) then screened the remaining publications and resolved any uncertainties through discussion with co-authors.

### Data extraction and quality assessment

We extracted the main characteristics of included studies using Microsoft Excel (Table S3). Two reviewers (SP, ME, or JR) independently extracted data from 10% of studies and disagreements were resolved through discussion among the three reviewers. One reviewer (SP) then extracted the remaining data and resolved any uncertainties through discussion with co-authors. We used the Joanna Briggs Institute (JBI) Critical Appraisal Tools to assess the quality of included studies.^44^ We categorised studies as ‘low’, ‘medium’, or ‘high’ quality for each JBI appraisal tool using the scoring system from Carter and colleagues (Table S4).^45^ We did not exclude studies based on quality scores.

### Synthesis of results

We used a framework synthesis approach to categorise qualitative and quantitative data from included studies into the nine *a-priori* health domains.^46^ We reviewed the extracted data to identify patterns, strengths, and limitations. Additionally, we categorised studies based on their reporting of analyses stratified by the sex, gender, and sexual identity of participants; and the proportion of First Nations participants. We mapped the data to identify trends in publication dates and sample locations. Trends in study characteristics and health domains were examined separately for studies involving people incarcerated or previously incarcerated in prisons, and studies of youth detention.

## Results

**Fig. 1:**
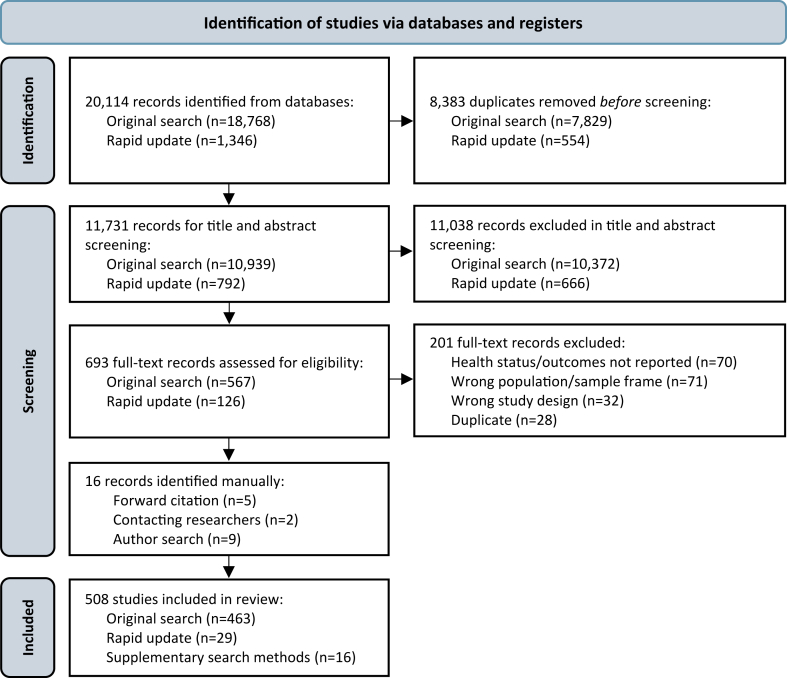
**PRISMAScR flow diagram**.

The search returned 20,114 records (18,768 from the initial search, 1346 from the update, and 16 identified manually; Fig. 1). After de-duplication, 11,731 unique records underwent title and abstract screening, and 11,038 were excluded. Of the 693 records that underwent full-text screening, 201 were excluded, leaving 492 for data extraction. Supplementary search methods identified 16 additional articles: nine from publication lists of prominent researchers in the field, five from reference lists of included studies, and two through professional networks. This yielded a final set of 508 peer-reviewed studies (Table S5).

### Quality of appraised studies

We assessed 359 studies (71%) as high quality using the JBI critical appraisal tools, with the remaining studies (n = 149, 29%) scoring medium quality. However, there was a notable risk of bias among quantitative studies: 124 studies (26%) did not identify or discuss the management of confounding factors, 96 (20%) did not adequately describe their statistical analysis methods, and 53 studies (11%) failed to report or inadequately described their participant inclusion criteria. Among the qualitative studies, 18 (46%) lacked a statement locating the researcher's cultural or theoretical orientation, and 17 (44%) did not discuss the potential influence of the researcher on data collection or interpretation.

### Trends in publication frequency

**Fig. 2:**
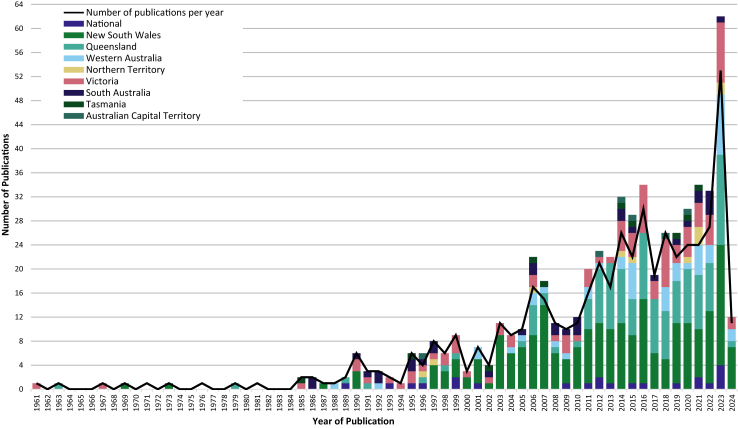
**Trends in publications over time by year and location.** Note. The stacked bars in a given year can sum to greater than 100% because 39 included studies involved cohorts from multiple jurisdictions. Data for 2024 includes only studies published up to March 3, 2024.

The earliest article was published in 1961, followed by 13 publications between 1962 and 1989. From 1990 to 2023, there was a consistent upward trend in the number of publications (Fig. 2). Forty-eight studies (9%) were published in the 1990s, 97 (19%) in the 2000s, and 210 (41%) in the 2010s. Over half (n = 258/508, 51%) were published between 2015 and 2024.

### Jurisdictional comparisons

**Fig. 3:**
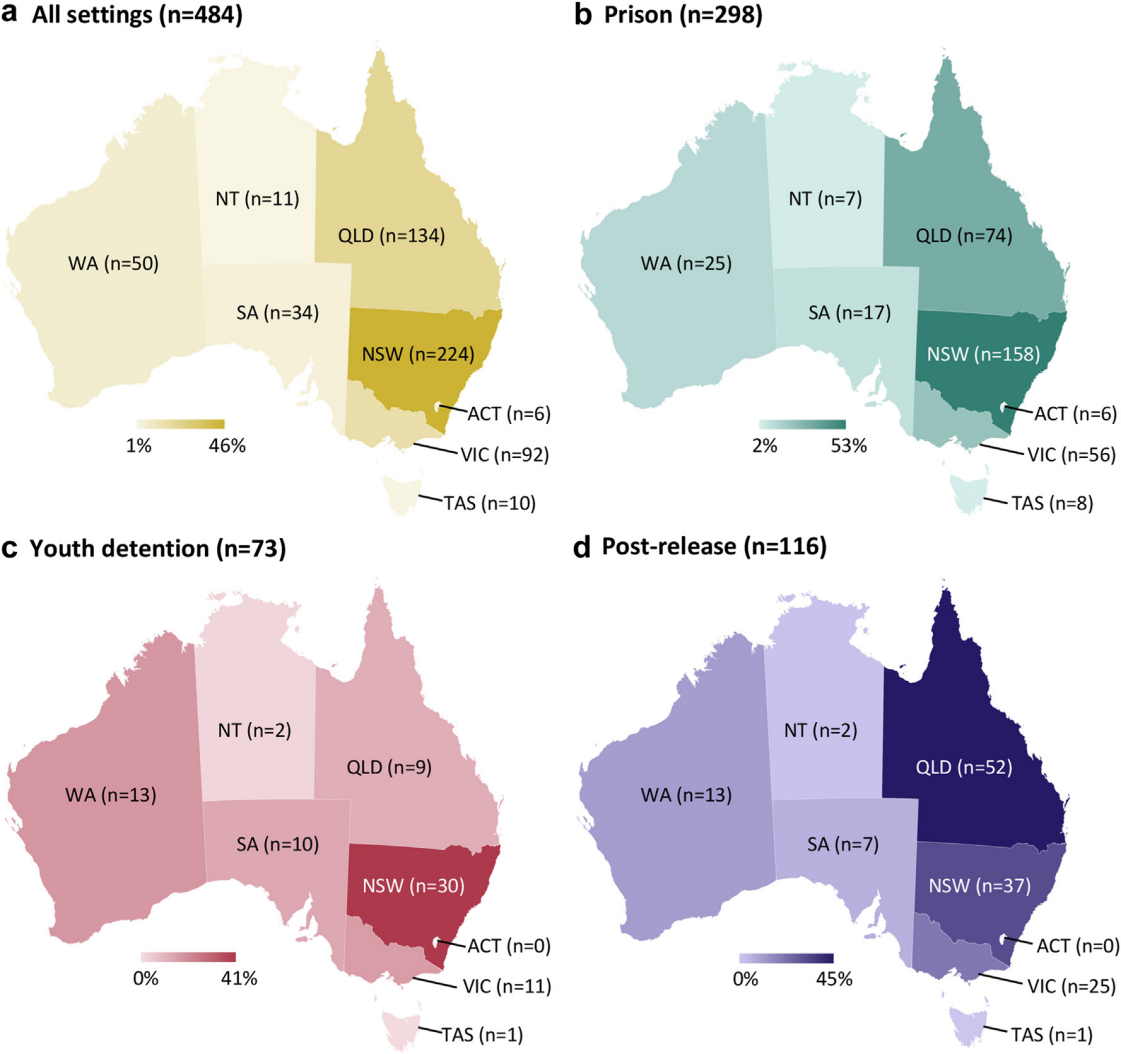
**Jurisdictional distribution of included studies**. a. All settings (n = 484): Studies across all incarceration settings. b. Prison (n = 298): Studies involving people incarcerated in prisons. c. Youth detention (n = 73): Studies involving young people in detention. d. Post-release (n = 116): Studies examining health after release from custody. Note. Studies that were national in scope (n = 22) and those that did not report the location of their sample (n = 2) are excluded from the jurisdictional distribution. ACT, Australian Capital Territory; NSW, New South Wales; NT, Northern Territory; QLD, Queensland; SA, South Australia; TAS, Tasmania; VIC, Victoria; WA, Western Australia.

Of 508 studies, only 22 (4%) involved data from every Australian State and Territory (i.e., national scope), 39 studies (8%) involved samples from two or more jurisdictions, and two (<1%) did not specify their sample location. Among the remaining 484 studies, 417 (86%) focused on the most populous jurisdictions—New South Wales, Queensland, or Victoria, while 86 (18%) involved samples from Western Australia, South Australia, the Northern Territory, Tasmania, or the Australian Capital Territory (Fig. 3).

Most studies (n = 298/484, 62%) involved people incarcerated in prisons, with 88% (n = 262/298) conducted in New South Wales, Queensland, or Victoria (Table S6). Studies of youth detention (n = 73/484, 15%) were primarily from New South Wales, Western Australia, and Victoria (n = 55/73, 75%). The jurisdictional distribution of post-release research (n = 116/484, 24%) differed considerably (Fig. 3), largely involving samples from Queensland. Notably, 29 (24%) of the 121 post-release studies did not intentionally sample people who had experienced incarceration but reported stratified results for this group.

### Studies involving adults exposed to incarceration

Most included studies (n = 431/508, 85%) involved people incarcerated or previously incarcerated in adult prisons; 82 of these studies (19%) focused on post-release health. Of the 431 studies involving adults who had experienced incarceration, six (1%) identified people who had also experienced youth detention: three identified people with histories in both prison and youth detention; one included separate samples of adults in prison and youth in detention; one included separate samples of individuals released from prison and from youth detention; and one reported on deaths that had occurred in Australian prisons and youth detention centres.

Most studies (n = 385/431, 89%) were quantitative (Table 1), and primarily cross-sectional (n = 180, 47%) or cohort studies (n = 148, 38%; prospective, n = 77; retrospective, n = 70; both retrospective and prospective, n = 1). Data linkage methods were used in 104 studies (27%), primarily investigating post-release factors related to health service use (n = 59), mortality rates (n = 32), or reincarceration (n = 20). Only eight studies (2%) were randomised controlled trials; five of these focused on substance use, and three assessed interventions to improve post-release health service use. Five (1%) studies were dedicated to infectious disease outbreak investigations. Of 37 qualitative studies, most (n = 24, 65%) involved thematic analysis of interviews, and nine (24%) examined court or medical records. These studies frequently focused on post-release healthcare experiences (n = 11); perceptions of prison-based health services (n = 6); or factors influencing hepatitis C transmission and/or treatment (n = 6).

**Table 1:** Methodological characteristics of included studies.

Characteristics	Quantitative		Qualitative		Mixed methods		All methods	
	Prison (n = 385)	Youth detention (n = 81)	Prison (n = 37)	Youth detention (n = 2)	Prison (n = 9)	Youth detention (n = 0)	Prison (n = 431)	Youth detention (n = 83)
Sampling frame								
Population-based	208 (54%)	73 (90%)	0 (0%)	0 (0%)	6 (67%)	–	214 (50%)	73 (88%)
Selected	177 (46%)	8 (10%)	37 (100%)	2 (100%)	3 (33%)	–	217 (50%)	11 (13%)
Sample size (range)	13–98,812	28–48,670	3–172	6–38	36–1075	–	3–98,812	6–48,670
Sub-population focus								
Male only	73 (19%)	9 (11%)	11 (30%)	1 (50%)	1 (11%)	–	85 (20%)	10 (12%)
Female only	21 (6%)	1 (1%)	12 (32%)	0 (0%)	2 (22%)	–	35 (8%)	1 (1%)
First Nations only	18 (5%)	1 (1%)	8 (22%)	0 (0%)	0 (0%)	–	26 (6%)	1 (1%)

Note. Studies coded as ‘selected’ specifically selected individuals with pre-existing health-related conditions, outcomes, or behaviours.

Of 431 studies, 111 (26%) sourced data from nine distinct cohorts. Of these, 13 (3%) reported data from the Prison and Transition Health (PATH) cohort,^47^ which focused on men who inject drugs and accounted for 16% of all Victoria-based publications. Twenty-four studies (6% of all prison studies) included individuals from the 1996, 2001, or 2009 New South Wales Inmate Health Surveys^48^, ^49^, ^50^; and accounted for 13% of all New South Wales-based studies. The Passports Study contributed 49 studies,^51^ representing 11% of prison studies and 31% of Queensland-based publications. Of these, 22 (45%) examined post-release outcomes, accounting for 52% of the post-release studies from Queensland and 19% of all included post-release studies. After mapping these data onto our health domains, the Passports cohort contributed to 26% of studies reporting cognitive disabilities, 19% reporting mental health status, and 17% on health service use. A similar concentration of research output was evident across the eight other distinct cohorts (Table S7).

Most studies of adults (n = 389/431, 90%) reported the sex and/or gender of participants, with 305 (71%) included more than one sex or gender. Almost half (n = 203, 47%) had a proportion of male participants, ranging between 70% and 98%, reflecting the over-representation of males in criminal justice systems^37^; 20% of studies focused exclusively on males.

Among the 35 (8%) exclusively sampling females, most studies were quantitative (n = 21/35, 60%), with sample sizes ranging from 29 to 22,363 participants; seven (33%) involved fewer than 100 participants. Most studies investigated mental disorders, substance use, and associations with offending behaviour (n = 9); health and transitional support needs (n = 8); or pregnancy-related outcomes linked to incarceration (n = 6). Six studies (17%) examined post-release health outcomes for women. Five (14%) of 35 female-only studies addressed experiences of First Nations women in prison and/or post-release, one of which explored the intersectional experiences of three First Nations transgender women.^52^

One-hundred and thirty-eight (32%) of 431 studies did not report the proportion of First Nations participants, while four (1%) exclusively involved non-Indigenous participants. Two-hundred and sixty-three studies (61%) included both First Nations and non-Indigenous participants, with the proportion of First Nations people ranging from 2% to 96%, averaging 26%. Twenty-six (6%) of 431 studies sampled only First Nations participants, primarily from New South Wales (n = 9), Queensland (n = 7), or Victoria (n = 7). Of these, 18 (69%) were quantitative (mostly cross-sectional; n = 14), and the remaining eight (31%) were qualitative. Quantitative studies of First Nations peoples had sample sizes ranging from 87 to 9353 participants, although most (n = 11, 61%) involved fewer than 300 participants. Most studies exclusively involving First Nations participants (n = 17/26, 65%) examined mental health, substance use, and associations with incarceration; few studies addressed cognitive disabilities (n = 4), non-communicable diseases (n = 3), or sexual and reproductive health (n = 3).

Twenty-six (6%) of 431 studies reported diverse gender identities (e.g., transgender or non-binary); however, five (19%) of these mis-reported transgender identity as a sexual orientation. Only 26 studies (6%) reported data on sexual identity, with the proportion of diverse sexualities reported in samples ranging from 2% to 35%. Nearly half of these studies (n = 11, 42%) originated from two cohorts,^51^^,^^53^ and predominantly involved community-based (n = 5) and prison-based (n = 4) cohorts of people who inject drugs. Only three studies reported separate results for participants who identified as non-heterosexual,^54^, ^55^, ^56^ and two compared health outcomes between heterosexual and non-heterosexual participants.^55^^,^^56^

**Fig. 4:**
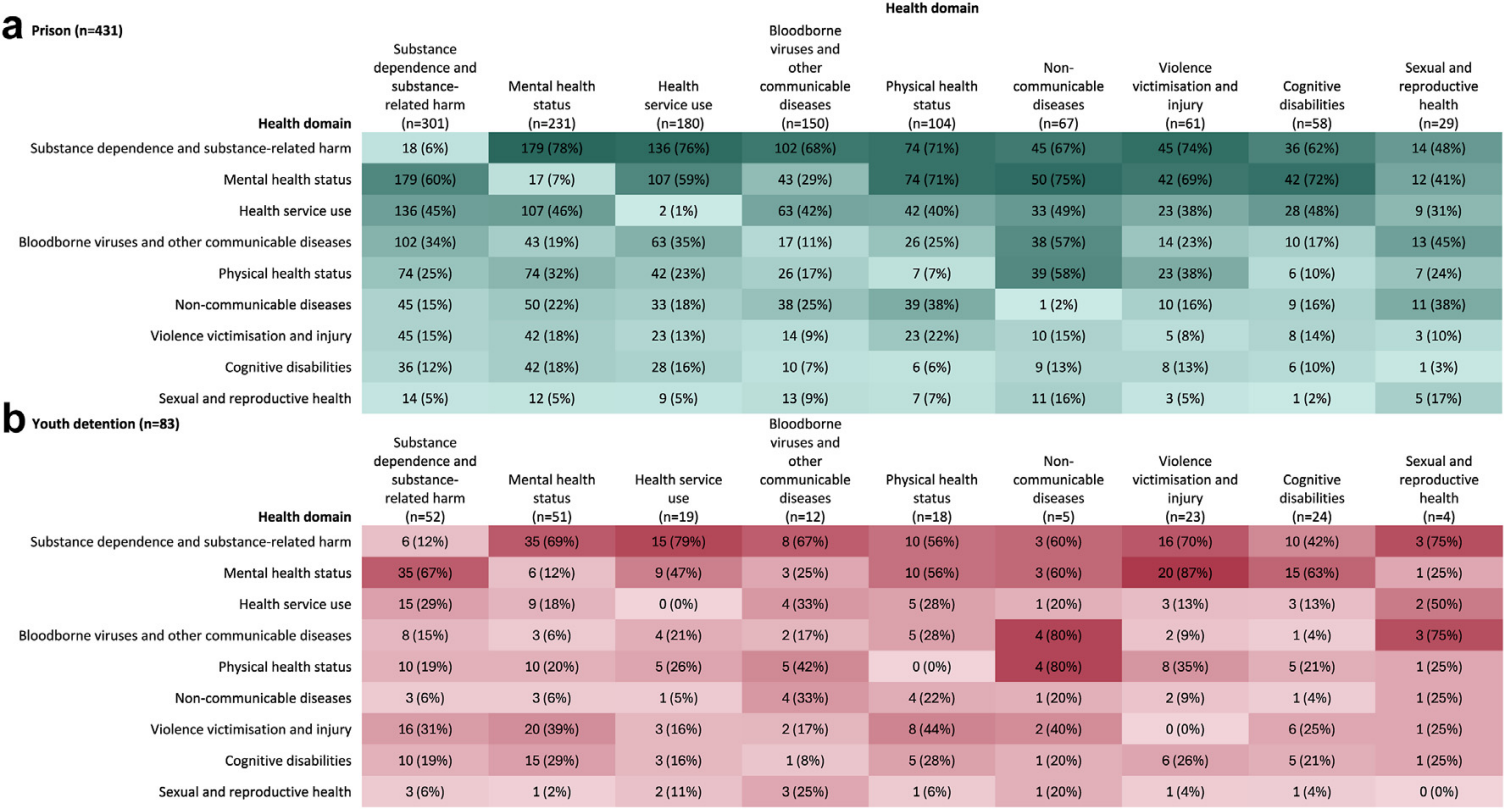
**Pairwise overlap between health domains for data reported in Australian studies**. a. Prison (n = 431): Studies involving people incarcerated or previously incarcerated in prisons. b. Youth detention (n = 83): Studies involving people incarcerated or previously incarcerated in youth detention. Note. Percentages displayed correspond to the values along the y-axis. Where a health domain intersects with itself, the number of studies indicates the proportion of data exclusively within that domain.

Most studies involving adults exposed to incarceration reported data pertaining to substance dependence and substance-related harm (70%) and/or mental health (54%; Table 2). There was considerable overlap across health domains (Fig. 4). Particularly, substance-related problems were frequently assessed alongside:

1. Mental health (n = 179, 60% of 301 substance-related studies), often identifying associations between psychopathology and offending behaviour;
2. Health service use (n = 136, 45%), including the impact, effectiveness, and acceptance of treatment for opioid dependence, and associations with reincarceration, relapse, and drug-related mortality; and
3. Bloodborne viruses (n = 102, 34%), including injecting drug use as a risk factor for hepatitis C infection and transmission within prisons and the community.

**Table 2:** Top three health conditions or outcomes researched per health domain.

A. Prison (n = 431)		B. Youth detention (n = 83)	
Health domain	n (%)	Health domain	n (%)
**Substance dependence and substance-related harm**	**301 (70%)**	**Substance dependence and substance-related harm**	**52 (63%)**
Injecting drug use	113 (26%)	Alcohol dependence	24 (29%)
Alcohol dependence	100 (23%)	Cannabis dependence; Substance dependence (any)^a^	17 (21%)
Substance dependence (unspecified)	78 (18%)	Injecting drug use	12 (15%)
**Mental health status**	**231 (54%)**	**Mental health status**	**51 (61%)**
Suicide ideation/attempt/death	98 (23%)	Suicide ideation/attempt/death	29 (35%)
Depression	68 (16%)	Anxiety; depression^a^	22 (27%)
Schizophrenia/schizoaffective disorder	59 (14%)	Self-harm ideation	21 (25%)
**Health service use**	**180 (42%)**	**Health service use**	**19 (23%)**
Treatment for opioid dependence	82 (19%)	Mental health-related service utilisation	5 (6%)
Psychiatric treatment (out-patient/in-patient/both)	32 (7%)	Hospital attendance; vaccination status^a^	4 (5%)
Hospital admissions	28 (7%)	Substance treatment use	3 (4%)
**Bloodborne viruses and other communicable diseases**	**150 (35%)**	**Bloodborne viruses and other communicable diseases**	**12 (15%)**
Hepatitis C	113 (26%)	Hepatitis C	8 (10%)
HIV	41 (10%)	Hepatitis B	6 (7%)
Hepatitis B	37 (9%)	HIV	3 (4%)
**Physical health status**	**104 (24%)**	**Physical health status**	**18 (22%)**
Mortality (natural causes or unspecified)	40 (9%)	Hearing problems/ear infections	6 (7%)
Poor self-reported physical health or functioning	20 (5%)	Mortality (cause unspecified)	5 (6%)
Poor self-reported general health	16 (4%)	Overweight and obesity	3 (4%)
**Non-communicable diseases**	**67 (16%)**	**Non-communicable diseases**	**5 (6%)**
Diabetes	23 (5%)	Asthma	3 (4%)
Cancer	19 (4%)	Deaths due to non-communicable diseases (any)	2 (2%)
Asthma	18 (4%)	Cancer; diabetes; epilepsy^a^	1 (1%)
**Violence victimisation and injury**	**61 (14%)**	**Violence victimisation and injury**	**23 (28%)**
Physical assault	31 (7%)	Sexual abuse	13 (16%)
Sexual assault/molestation/coercion	26 (6%)	Physical abuse	11 (13%)
Violence-related death	17 (4%)	Victim of violent crime or violence-related death	5 (6%)
**Cognitive disabilities**	**58 (14%)**	**Cognitive disabilities**	**24 (29%)**
Possible/borderline/diagnosed intellectual disability	38 (9%)	ADHD	15 (18%)
ADHD	8 (2%)	Borderline/diagnosed intellectual disability	8 (10%)
Organic brain disease	4 (1%)	Foetal alcohol spectrum disorder	7 (8%)
**Sexual and reproductive health**	**29 (7%)**	**Sexual and reproductive health**	**4 (5%)**
Any STI	10 (2%)	Chlamydia	3 (4%)
Chlamydia	10 (2%)	Gonorrhoea; syphilis; any STI^a^	2 (2%)
Syphilis; gonorrhoea^a^	7 (2%)	Herpes	1 (1%)

Note. Six studies identified people with experiences of youth detention and adult prison in the same sample. Percentages amount to greater than 100% due to multiple conditions/outcomes reported within individual studies.

a Each health condition/outcome listed was reported in the same number of studies.

### Studies involving people exposed to youth detention

Eighty-three studies (16% of all included studies) reported the health status of people incarcerated or previously incarcerated in youth detention. Of these, 81 (98%) were quantitative, and primarily cross-sectional (n = 60, 72%; Table 1). Twelve studies (15%) used data linkage methods, half of which (n = 6) measured rates and risk factors for mortality following contact with the youth justice system. One qualitative study explored young people's experiences of diagnostic assessment for foetal alcohol spectrum disorder (FASD) as part of a broader prevalence study,^57^ while another reviewed the medical records of six young men diagnosed with neutropenia during incarceration.^58^ Most studies (n = 54/83, 65%) involved fewer than 300 participants.

Of 83 studies, 28 (34%) reported data from ten distinct cohorts based in Victoria (n = 9, 32%), New South Wales (n = 7, 25%), Western Australia (n = 8, 29%), South Australia (n = 4, 14%), and Queensland (n = 2, 7%). After mapping these data onto our health domains, one Western Australian cohort accounted for 25% of all studies on cognitive disabilities and 57% of studies on FASD (Table S8).^57^ Additionally, one Victorian cohort accounted for 25% of all youth detention studies examining bloodborne viruses.^59^

Seventy-six (92%) youth detention studies reported sex and gender characteristics for their samples, with 65 studies (78%) involving cohorts of more than one sex or gender. Three studies reported involving transgender young people, two of which reported data from one cohort.^57^ Two studies based on the same sample reported participants’ sexual identities,^60^^,^^61^ with 8% identifying as lesbian, gay, bisexual, or queer; however, neither study stratified the results by sexual identity. Ten studies (12%) included male-only cohorts based in New South Wales (n = 4, 40%), Victoria (n = 4, 40%), and South Australia (n = 2, 20%). Sixty-six (80%) of 83 studies reported the proportion of First Nations participants, ranging from 6% to 88%. One study specifically focused on First Nations health, involving 47 Indigenous young people incarcerated in Queensland.^62^

Most studies involving young people exposed to youth detention focused on substance dependence (63%) and/or mental health (61%; Table 2). A distinct pattern of overlap was evident across health domains (Fig. 4), with substance-related issues frequently assessed alongside:

1. Mental health (n = 35, 67% of 52 substance-related studies), often examining prevalence and patterns of co-occurring substance use and mental health disorders, and associations with suicide, self-harm, and reincarceration;
2. Violence victimisation and injury (n = 16, 31%), exploring relationships between histories of trauma, mental health challenges, and offending behaviour; and
3. Health service use (n = 15, 29%), investigating service needs and histories of service use related to substance dependence and mental health.

## Discussion

This is the first comprehensive synthesis of health research involving people exposed to Australian prisons and youth detention. Our findings showcase the considerable progress made towards understanding the health of this population. Of 508 included studies, most were published after the year 2000 and in Australia's most populous states. Research predominantly involved adult males—as they comprise the largest proportion of incarcerated Australians—while there was less evidence on the health of young people, women, First Nations peoples, and sexual and gender minorities. There was an overwhelming focus on mental illness, substance dependence, bloodborne viruses, and their associations with offending. While these issues are highly prevalent in incarcerated populations,^13^^,^^63^ we identified significant gaps in geographical representation, studies of important sub-populations, and studies of many health conditions that impose a substantial health burden on people who experience incarceration. Additionally, our findings highlight methodological limitations that constrain our ability to capture the complexity of health outcomes experienced by this population.

Most studies were cross-sectional, providing limited insight into the longer-term health trajectories of people who experience incarceration, particularly after release from custody. Longitudinal, primary data collection studies are complicated by challenges in retaining participants due to marginalisation and poor health.^64^^,^^65^ Strategies to reduce attrition in ‘hard-to-reach’ populations can be time-consuming and resource-intensive; these challenges contribute to the under-representation of socially disadvantaged groups in primary research, particularly longitudinal studies.^66^^,^^67^ However, data linkage methods can help alleviate some of these barriers while following large cohorts over time.^45^^,^^68^ Integrating administrative health data with data from non-health sectors (e.g., criminal justice, housing, education) can further identify patterns of inequity among those often excluded.^69^, ^70^, ^71^ Despite Australia's substantial investment in national data linkage infrastructure,^72^ our review identified only 54 studies (11%) using population-level administrative data linkage to examine health outcomes after incarceration. Future research in this area would greatly benefit from closer engagement with health and justice data custodians to establish ongoing, multi-sectoral linkages for both new and existing cohort studies.

Australia's less populous jurisdictions were notably under-represented in this review, despite their disproportionately high incarceration rates, particularly for First Nations peoples. For example, the Northern Territory has the highest adult incarceration rate in the country, at 1107 per 100,000^37^—over twice the highest rate in the Western Pacific (Tonga, 516 per 100,000 people).^7^ This has important and growing implications for the health of First Nations communities in Australia. For example, from 2020 to 2021, the rate of youth detention in the Northern Territory rose by 79%, and nearly 100% of those detained were First Nations children.^73^^,^^74^ Incarceration in this region is strongly associated with risky alcohol use, while in other jurisdictions, illicit drug use plays a larger role.^75^^,^^76^ Despite these important sub-national differences, we identified only 11 studies (2%) from the Northern Territory, with just two (<1%) focusing on First Nations peoples. Australia's vast geography includes extensive regional and remote areas. Despite this, 86% of included studies were from Australia's most populous jurisdictions: New South Wales, Queensland, and Victoria. Together, these states account for 77% of the Australian population,^77^ 68% of the prison population, and 42% of incarcerated First Nations peoples,^37^^,^^73^ yet account for only 36% of Australia's land mass.^78^ Remoteness and area disadvantage can create distinct health needs in less densely populated regions.^79^ However, the dearth of jurisdiction-specific evidence for many health issues renders accurate sub-national comparisons fraught, if not impossible. National research with jurisdictional comparisons would help address these evidence gaps, enabling examination of how social, structural, economic, geographical, and environmental factors impact the health of justice-involved populations. Similarly, organised comparative research at the regional level would inform ongoing efforts to reduce health inequities.^6^^,^^80^^,^^81^

Few studies examined the health of young people, women, First Nations peoples, or people with diverse gender and sexual identities. Though many studies included First Nations peoples within their samples, the lack of exclusive focus risks overlooking community-led priorities and culturally specific health needs. For marginalised communities, intersectional identities—such as gender, sexuality, race, and class—can further exacerbate health and social inequalities, heighten experiences of discrimination and victimisation, and negatively affect health outcomes.^82^^,^^83^ Such vulnerability is reflected among incarcerated women, who often have histories of trauma, poverty, and physical and sexual violence that not only contribute to challenges in accessing adequate healthcare, but also perpetuate a cycle of disadvantage.^84^, ^85^, ^86^, ^87^ In addition to gender-specific health needs, varied identities within female populations (e.g., Indigenous women, transgender women, older women) contribute to distinct health inequities that warrant targeted research.^52^^,^^88^ Representing these identities in research is fundamental to identifying and responding to their unique needs.

A shift towards equity-oriented research requires involving people with lived experience of incarceration in research agendas. Education, mental health, hygiene, and transitional programmes have been cited as critical research priorities by incarcerated people.^20^ These community-driven priorities underscore that research should extend beyond immediate health concerns, including addressing structural and systemic determinants of health inequity. Our review also identified a disproportionate focus on some health issues, and relative under-investment in others. For example, 82% of all studies included a focus on mental health and/or substance use. Although these are important drivers of health burden among people who experience incarceration, they are also important drivers of reoffending, which may, in part, explain the research focus on these conditions.^89^ Similarly, 35% of studies of incarcerated and previously incarcerated adults included a focus on communicable diseases, particularly hepatitis C, whereas only 16% considered non-communicable diseases. Managing infectious diseases in people cycling between prison and community settings prevents the spread of infection into community settings,^68^ such that a focus on infectious disease in prisons has clear public health utility. However, there is good evidence that among people who experience incarceration, a much greater proportion of the burden of disease is accounted for by non-communicable diseases: In a harmonised analysis of 75,427 deaths among 1,471,526 adults released from prisons in eight countries, Borschmann and colleagues found that non-communicable diseases accounted for 39.4% of deaths, while infectious diseases accounted for only 4.8%.^90^ Given that chronic disease is also the leading contributor to disease burden in Australia and many other countries in the Western Pacific region, and is known to disproportionately affect socially disadvantaged populations,^91^ our findings suggest that the distribution of research investment is not currently proportionate to the disease burden for people who experience incarceration in Australia.

Despite an exponential increase in research on the health of justice-involved people in Australia, important questions remain unanswered. More high-quality research is needed across the full spectrum of health issues affecting people in contact with the criminal justice system. Elevated rates of health service use after release from incarceration highlight the complex multimorbidity and often preventable or treatable health conditions prevalent among incarcerated populations.^92^^,^^93^ Investment in prevention, early intervention, and health promotion is essential for reducing health inequities and alleviating the associated, avoidable economic burden on health systems.^94^ Aligning with the WHO's call for ‘prison health in all policies’,^29^ all relevant population-level policies must include people in contact with the criminal justice system, to facilitate coordination of care and ensure that public investment is proportional to need. For instance, despite numerous studies indicating high rates of self-harm and suicidality among young people in and after youth detention,^95^^,^^96^ there are no strategies in the Australian National Mental Health and Suicide Prevention Plan targeting this specific population.^97^ National frameworks that are inclusive of people who experience incarceration would meaningfully contribute to data collection and dissemination, service planning and provision, safety and quality standards, improved health outcomes, and population health equity.^13^^,^^98^

Our review had three main limitations. First, due to the diversity of study designs, we assessed study quality based on a ‘best fit’ approach to the available JBI critical appraisal tools.^44^ This allowed us to evaluate studies across varied methodologies, although other reviewers might assess them differently. Second, we did not conduct a systematic review and meta-analysis of the identified research. Although beyond the scope of our broad review, this would be a valuable direction for future research. Lastly, we excluded grey literature from our review, which resulted in the exclusion of some potentially relevant studies but also provided a degree of quality control for included studies.

## Conclusions

This review comprehensively synthesised research reporting on the health of people who experience incarceration in Australia, highlighting both the progress made and the significant gaps that persist in understanding the health needs of this highly marginalised population. Despite substantial growth in research output in the last two decades, critical health issues, key populations, and most jurisdictions remain under-studied. Similar evidence synthesis in other countries and at the regional level would support efforts to address health inequities across the Western Pacific. Targeted, equity-oriented research and investment are essential to inform effective, evidence-driven health policies and interventions that reduce disparities and improve outcomes for all people who experience incarceration.

## Contributors

SK conceptualised the study. SP conducted searches, screening, extraction, and analysis, and drafted the original manuscript. SK, LP, AC, and RS provided supervision. All authors contributed to the interpretation, reviewed and edited the manuscript, and approved the final paper.

## Data sharing statement

All data sources analysed and produced in this review are included in the References and Supplementary Materials.

## Editor note

The Lancet Group takes a neutral position with respect to territorial claims in published maps and institutional affiliations.

## Declaration of interests

We declare no competing interests.
