# Supplementary material for: Health and incarceration research in Australia: a scoping review
Source: Lancet Reg Health West Pac. 2025 Mar 18;56:101500. doi: 10.1016/j.lanwpc.2025.101500 (PMC11960673; doi:10.1016/j.lanwpc.2025.101500)
Supplement: Supplementary Tables [file mmc1.pdf]

Contents

Supplementary Table 1: Search strategy and results from CINAHL Ultimate electronic database 2

Supplementary Table 2: Inclusion and exclusion criteria 4

Supplementary Table 3: Data fields extracted from eligible studies 5

Supplementary Table 4: JBI Critical Appraisal Tools scoring system 6

Supplementary Table 5: Summary of included studies 7

Supplementary Table 6: Number of included studies by sample setting and location 50

Supplementary Table 7: Proportion of data reported across health domains for distinct cohorts—prison (n=431) 51

References 53

Supplementary Table 1: Search strategy and results from CINAHL Ultimate electronic database

No.	Search String	Search Options	Results
S1	TI (prison* OR incarcerat* OR decarcer* OR custod* OR imprison* OR internment OR detention OR detain* OR sentenc* OR inmate* OR jail* OR penitentiary OR gaol* OR correctional* OR probation* OR parole* OR remand OR offend* OR convic* OR felon* OR criminal* OR judic* OR juvenile* OR "solitary confine*" OR "reintegration" OR "institutional release" OR "re-entry") OR TI (depriv* N2 (libert* OR freedom*)) OR TI (secure N2 (facilit* OR center* OR centre* OR complex* OR unit*)) OR TI (holding N2 (facilit* OR center* OR centre* OR complex* OR unit*)) OR TI (punitive N2 (facilit* OR center* OR centre* OR complex* OR unit* OR system* OR setting* OR service* OR environment*)) OR TI (correction* N2 (facilit* OR center* OR centre* OR complex* OR unit* OR system* or setting* OR service* OR environment*)) OR TI (legal N2 (facilit* OR system* OR setting* OR service* OR involve*)) OR TI (penal N2 (facilit* OR centre* OR center* OR complex* OR unit* OR system* OR setting* OR service* OR environment*)) OR TI (justice N2 (youth* OR juvenile* OR adolescen* OR system* OR setting*))	Limiters: English Language, peer-reviewed, exclude MEDLINE records<br>Expanders: Apply equivalent subjects<br>Search modes: Boolean/Phrase	14266
S2	AB (prison* OR incarcerat* OR decarcer* OR custod* OR imprison* OR internment OR detention OR detain* OR sentenc* OR inmate* OR jail* OR penitentiary OR gaol* OR correctional* OR probation* OR parole* OR remand OR offend* OR convic* OR felon* OR criminal* OR judic* OR juvenile* OR "solitary confine*" OR "reintegration" OR "institutional release" OR "re-entry") OR AB (depriv* N2 (libert* OR freedom*)) OR AB (secure N2 (facilit* OR center* OR centre* OR complex* OR unit) OR AB (holding N2 (facilit* OR center* OR centre* OR complex* OR unit*)) OR AB (punitive N2 (facilit* OR center* OR centre* OR complex* OR unit* OR system* OR setting* OR service* OR environment*)) OR AB (correction* N2 (facilit* OR center* OR centre* OR complex* OR unit* OR system* or setting* OR service* OR environment*)) OR AB (legal N2 (facilit* OR system* OR setting* OR service* OR involve*)) OR AB (penal N2 (facilit* OR centre* OR center* OR complex* OR unit* OR system* OR setting* OR service* OR environment*)) OR AB (justice N2 (youth* OR juvenile* OR adolescen* OR system* OR setting*))	Limiters: English Language, peer-reviewed, exclude MEDLINE records<br>Expanders: Apply equivalent subjects<br>Search modes: Boolean/Phrase	25206
S3	TI (health* OR disease* OR illness* OR disorder* OR infect* OR disab* OR impair* OR mortalit* OR morbidit* OR death* OR wellbeing OR "well-being" OR "mental health" OR cognitive OR psych* OR therap* OR neuro* OR "Indigenous health" OR medicare OR suicid* OR "self-harm*" OR "self-injury" OR "nonsuicidal self-injury" OR NSSI OR "post-traumatic" OR PTSD OR "attention deficit" OR ADHD OR "oppositional defiant" OR "conduct disorder" OR depress* OR anxiety OR stress* OR schizophrenia OR bipolar OR psychopath* OR "blood-borne" OR BBV OR BBVs OR communicable or "non communicable" OR "non-communicable" OR noncommunicable OR virus OR viral OR vaccin* OR covid* OR coronavirus* OR sarscov* OR "sars-cov*" OR influenza OR asthma OR diabetes OR hepatitis OR hepatic OR tuberculosis OR cancer* OR arthritis OR cardio* OR "otitis media" OR "ear infection*" OR "hearing loss" OR obes* OR excercis* OR weigh* OR injur* OR "brain injur*" OR trauma* OR "human papillomavirus" OR HIV OR AIDs OR STD OR STDs OR STI OR STIs OR "sexually transmitted" OR herpes OR "genital wart*" OR syphilis OR chlamydia* OR monkeypox OR gonorrhoea OR pregnan* OR reproducitv* OR menstr* OR illicit OR drug* OR substance* OR tobacco OR smok* OR alcohol* OR addict* OR heroin OR cannabis OR meth* OR amphetamine* OR nicotine* OR needle* OR tattoo* OR pierc* OR opiod* OR violen* OR assault* OR condom* OR dental* OR homeless* OR accom* OR "general practitioner" OR counsell* OR medic* OR "risk* behav*" OR "adverse childhood experience*" OR ACE OR "ACEs") OR TI (self N2 (harm OR destructive OR inflict* OR injur*)) OR TI (unsafe N2 (sex* OR drug* OR substance* OR inject*)) OR TI ("at-risk" N5 (sex* OR drug* OR substance* OR alcohol* OR behav*)) OR TI (sex* N2 (exploit* OR educat* OR promot*))	Limiters: English Language, peer-reviewed, exclude MEDLINE records<br>Expanders: Apply equivalent subjects<br>Search modes: Boolean/Phrase	1296237
S4	AB (health* OR disease* OR illness* OR disorder* OR infect* OR disab* OR impair* OR mortalit* OR morbidit* OR death* OR wellbeing OR "well-being" OR "mental health" OR cognitive OR psych* OR therap* OR neuro* OR "Indigenous health" OR medicare OR suicid* OR "self-harm*" OR "self-injury" OR "nonsuicidal self-injury" OR NSSI OR "post-traumatic" OR PTSD OR "attention deficit" OR ADHD OR "oppositional defiant" OR "conduct disorder" OR depress* OR anxiety OR stress* OR schizophrenia OR bipolar OR psychopath* OR "blood-borne" OR BBV OR BBVs OR communicable or "non communicable" OR "non-communicable" OR noncommunicable OR virus OR viral OR vaccin* OR covid* OR coronavirus* OR sarscov* OR "sars-cov*" OR influenza OR asthma OR diabetes OR hepatitis OR hepatic OR tuberculosis OR cancer* OR arthritis OR cardio* OR "otitis media" OR "ear infection*" OR "hearing loss" OR obes* OR excercis* OR weigh* OR injur* OR "brain injur*" OR trauma* OR "human papillomavirus" OR HIV OR AIDs OR STD OR STDs OR STI OR STIs OR "sexually transmitted" OR herpes OR "genital wart*" OR syphilis OR chlamydia* OR monkeypox OR gonorrhoea OR pregnan* OR reproducitv* OR menstr* OR illicit OR drug* OR substance* OR tobacco OR smok* OR alcohol* OR addict* OR heroin OR cannabis OR meth* OR amphetamine* OR nicotine* OR needle* OR tattoo* OR pierc* OR opiod* OR violen* OR assault* OR condom* OR dental* OR homeless* OR accom* OR "general practitioner" OR counsell* OR medic* OR "risk* behav*" OR "adverse childhood experience*" OR ACE OR "ACEs") OR AB (self N2 (harm OR destructive OR inflict* OR injur*)) OR AB (unsafe N2 (sex* OR drug* OR substance* OR inject*)) OR AB ("at-risk" N5 (sex* OR drug* OR substance* OR alcohol* OR behav*)) OR AB (sex* N2 (exploit* OR educat* OR promot*))	Limiters: English Language, peer-reviewed, exclude MEDLINE records<br>Expanders: Apply equivalent subjects<br>Search modes: Boolean/Phrase	1310185

No.	Search String	Search Options	Results
S5	TI Australia* OR "Australian Capital Territory" OR "New South Wales" OR Victoria* OR Queensland OR "South Australia*" OR Tasmania* OR "Western Australia*" OR "Northern Territory"	Limiters: English Language, peer-reviewed, exclude MEDLINE records<br>Expanders: Apply equivalent subjects<br>Search modes: Boolean/Phrase	24005
S6	AB Australia* OR "Australian Capital Territory" OR "New South Wales" OR Victoria* OR Queensland OR "South Australia*" OR Tasmania* OR "Western Australia*" OR "Northern Territory"	Limiters: English Language, peer-reviewed, exclude MEDLINE records<br>Expanders: Apply equivalent subjects<br>Search modes: Boolean/Phrase	32832
S7	S1 OR S2	Expanders: Apply equivalent subjects<br>Search modes: Boolean/Phrase	30945
S8	S3 OR S4	Expanders: Apply equivalent subjects<br>Search modes: Boolean/Phrase	1912341
S9	S5 OR S6	Expanders: Apply equivalent subjects<br>Search modes: Boolean/Phrase	45432
S10	S7 AND S8 AND S9	Expanders: Apply equivalent subjects<br>Search modes: Boolean/Phrase	707

Supplementary Table 2: Inclusion and exclusion criteria

Component	Inclusion Criteria	Exclusion Criteria
Population	Studies examining individuals incarcerated or previously incarcerated in Australian prison or youth detention settings.	Studies of populations who have been deprived of their liberty in other settings, including immigration detention, child protection, police watch houses, and forensic psychiatric facilities.
	Studies that sample populations who have been deprived of their liberty in other settings or under non-custodial arrangements (e.g., community-based order), if the publication includes data specific to individuals incarcerated or previously incarcerated in Australian prison or youth detention settings.	Studies of populations under non-custodial arrangements (e.g., community-based supervision order).
Concept	Studies examining health needs, conditions, interventions, supports, service use, and related outcomes (as categorised in nine *a priori* domains).	Studies that do not examine health as defined within the nine *a priori* domains.<br>Studies that examine biomarkers rather than health outcomes or diagnoses.<br>Studies that examine health-related knowledge.<br>Studies on perceptions of health services or accessibility.<br>Studies that examine non-diagnostic markers of cognitive impairment or personality (e.g., aggression, visuospatial ability, or executive planning).<br>Studies that report binary measures of self-reported lifetime substance use.
	Studies examining risky health behaviours and associated health outcomes (e.g., injecting drug use and prevalence of hepatitis C infection).	Studies that examine risky health behaviours without reporting associated health status/outcomes.
	English language.	Written in a language other than English.
Context	Studies must be of populations within Australian states and territories.	Study populations outside Australia.
	Studies that compare populations between Australia and other countries, if the publication includes data specific to individuals incarcerated or previously incarcerated in Australia.	
	No limit on study date.	
Study Design	Empirical, qualitative or quantitative, peer-reviewed research studies.	Reviews, case studies, dissertations, conference proceedings, commentaries/opinion editorials, and other grey literature.

Note. Studies published before the modern peer-review process became common practice (mid-1970s) were included regardless of peer review status (n=5).

Supplementary Table 3: Data fields extracted from eligible studies

Main fields	Sub-fields
1. Author(s)	
2. Title	
3. Year of publication	
4. Year(s) study was conducted	
5. Australian jurisdiction(s) (state/territory name(s) or national)	International studies: Australian sample (*n,* %)
6. Detention setting (prison or youth detention centres)	Private or government-run facilities<br>Other setting: Post-release (i.e., focused on cohort released from prison or youth-detention setting), community-based (e.g., reports health status for a sub-sample with a history of incarceration in prison or youth detention settings), or other (e.g., includes people with history of incarceration in police cells or people sentenced to community-based orders).
7. Health domain(s) (mental health; cognitive disabilities; substance-related; non-communicable diseases; physical health status; sexual and reproductive health; violence-related; health service utilisation)	
8. Type of institutions involved with the study (e.g., Academia, government, not-for-profit, consultant)	Funding source
9. Methods (quantitative, qualitative, mixed)	Study design (e.g., data linkage, cross-sectional, retrospective cohort)<br>Secondary analysis of data (i.e., no primary data collection)<br>Sampling frame (representative or selected)<br>Participant inclusion criteria<br>Source(s) of health information (self-report, research interview, prison medical records, administrative data)
10. Demographic characteristics of study participants (number; sex; age; gender diversity; Aboriginal and Torres Strait Islander status; sexuality)	
11. Exposure(s)	
12. Outcome(s) of interest	Outcome measures
13. Outcome results	

Supplementary Table 4: JBI Critical Appraisal Tools scoring system

Quality scoring was applied using Carter and colleagues (2022) scoring system and justification.[1] Low, medium, and high quality were determined depending on the number of items in each JBI checklist. Publications were considered low quality if they scored 0 on at least 50% of the items, medium quality if they scored 1 on at least 50% of the items, and high quality if they scored 2 on at least 50% of the items.

JBI scoring limit calculations per checklist

**Checklist for cross-sectional analytical studies: 8-item version**			
Quality:	Low	Medium[a]	High[b]
Score:	0 - 3	4 - 11	12 - 16
**Checklist for quasi-experimental studies: 9-item version**			
Quality:	Low	Medium[c]	High[d]
Score:	0 - 4	5 - 13	14 - 18
**Checklist for studies reporting prevalence data: 9-item version**			
Quality:	Low	Medium[c]	High[d]
Score:	0 - 4	5 - 13	14 - 18
**Checklist for case series: 10-item version**			
Quality:	Low	Medium[e]	High[f]
Score:	0 – 4	5 – 14	15 – 20
**Checklist for case control studies: 10-item version**			
Quality:	Low	Medium[e]	High[f]
Score:	0 – 4	5 – 14	15 – 20
**Checklist for qualitative research: 10-item version**			
Quality:	Low	Medium[e]	High[f]
Score:	0 – 4	5 – 14	15 – 20
**Checklist for cohort studies: 11-item version**			
Quality:	Low	Medium[g]	High[h]
Score:	0 – 5	6 – 16	17 – 22
**Checklist for economic evaluations: 11-item version**			
Quality:	Low	Medium[g]	High[h]
Score:	0 – 5	6 – 16	17 – 22
**Checklist for randomized controlled trials: 13-item version**			
Quality:	Low	Medium[i]	High[j]
Score:	0 – 6	7 – 19	20 – 26

Lower limit justification:
[a]4*1 + 4*0 = 4; [b]4*2 + 4*1 = 12
[c]5*1 + 4*0 = 5; [d]5*2 + 4*1 = 14
[e]5*1 + 5*0 = 5; [f]5*2 + 5*1 = 15
[g]6*1 + 5*0 = 6; [h]6*2 + 5*1 = 17
[i]7*1 + 6*0 = 7; [j]7*2 + 6*1 = 20

Supplementary Table 5: Summary of included studies

Author (year)	Study aim(s)	Location	Setting	Mapped health domains	Sample
Abbott et al. (2016) [2]	Determine expectations and the lived experiences of general practitioner contact among women prior to and after release from prison	NSW	Prison<br>Other	Mental health status<br>Substance dependence and substance-related harm<br>Health service utilisation	*N* = 69; 100% female
Abbott et al. (2016) [3]	Identify health service provision available to women incarcerated in prisons, their health needs, and recommendations for improvements within prisons and post-release	NSW	Post-release<br>Prison	Mental health status<br>Substance dependence and substance-related harm<br>Bloodborne viruses and other communicable diseases<br>Non-communicable diseases<br>Physical health status<br>Sexual and reproductive health<br>Health service utilisation	*N* = 231; 100% female
Abbott et al. (2017) [4]	Explore experiences of healthcare within prisons and post-release among women with a history of incarceration	NSW	Prison	Mental health status<br>Substance dependence and substance-related harm<br>Physical health status<br>Health service utilisation	*N* = 40; 100% female
Abbott et al. (2017) [5]	Explore the transfer of health information and continuity of healthcare between prison and the community among women experiencing incarceration	NSW	Prison	Mental health status<br>Substance dependence and substance-related harm<br>Non-communicable diseases<br>Health service utilisation	*N* = 212; 100% female
Abbott et al. (2022) [6]	Capture experiences of and attitudes towards general practice care for women with a history of substance dependence and contact with the justice system	NSW	Prison<br>Post-release	Substance dependence and substance-related harm<br>Health service utilisation	*N* = 39; 100% female
Adams et al. (2009) [7]	Describe the implementation of a mental health screening unit in an NSW prison	NSW	Prison	Mental health status<br>Cognitive disabilities	*N* = 604; 100% male
Aitken et al. (2002) [8]	Measure exposure to hepatitis C, hepatitis B, and HIV among people who inject steroids	VIC	Community<br>Prison history	Substance dependence and substance-related harm<br>Bloodborne viruses and other communicable diseases	*N* = 63; *n* = 54 male; *n* = 9 female
Alan et al. (2011) [9]	Describe rates of inpatient hospital use within 12 months after release from prison	WA	Post-release<br>Prison	Mental health status<br>Substance dependence and substance-related harm<br>Non-communicable diseases<br>Health service utilisation	*N* = 7414; *n* = 6,524 male
Allnutt et al. (2008) [10]	Explore the relationship between temperament, substance use, and psychopathology among people in prisons	NSW	Prison	Mental health status<br>Substance dependence and substance-related harm	*N* = 1322; 84% male
Amarasena et al. (2015) [11]	Examine the dental health of Aboriginal adults living in the NT	NT	Prison<br>Other	Physical health status	Prison cohort: *n* = 104 (sex/gender not reported)
Andrews et al. (2011) [12]	Assess the sensitivity of the National Coroners Information System in identifying mortality among people incarcerated and released from prisons	QLD	Post-release<br>Prison	Substance dependence and substance-related harm<br>Physical health status	*N* = 677 (sex/gender not reported)

Author (year)	Study aim(s)	Location	Setting	Mapped health domains	Sample
Andrews and Kinner (2012) [13]	Examine the nature of accidental drug-related deaths compared to deaths from other causes among people released from prison	National	Post-release<br>Prison	Mental health status<br>Substance dependence and substance-related harm<br>Physical health status<br>Health service utilisation	*N* = 388; *n* = 367 male
Ashdown and Kilvert (1979) [14]	Describe the diagnosis, clinical factors, and management of granuloma inguinale	QLD	Prison	Sexual and reproductive health	*N* = 13; *n* = 7 male; *n* = 6 female
Aung et al. (2023) [15]	Understand reasons for not utilising treatment for hepatitis C and identify pathways that increase HCV treatment uptake	VIC	Community<br>Prison history	Substance dependence and substance-related harm<br>Health service utilisation	*N* = 15; *n* = 10 male; *n* = 5 female
Austin et al. (2014) [16]	Investigate suicide deaths in SA prisons and compare to non-suicide deaths	SA	Prison	Mental health status<br>Substance dependence and substance-related harm<br>Non-communicable diseases<br>Physical health status	*N* = 48 (sex/gender not reported)
Awofeso et al. (2001) [17]	Describe the investigation into an influenza outbreak in an NSW prison	NSW	Prison	Bloodborne viruses and other communicable diseases	*N* = 17 (sex/gender not reported)
Awofeso et al. (2001) [18]	Examine hepatitis B vaccine uptake among people in prison, and whether hepatitis C status influences vaccine uptake	NSW	Prison	Bloodborne viruses and other communicable diseases<br>Health service utilisation	First cohort: *n* = 455<br>Second cohort: *n* = 582<br>(sex/gender not reported)
Baidawi (2016) [19]	Measure the psychological distress levels among older people who are experiencing incarceration and compare with a younger cohort and an older community sample	NSW	Prison	Mental health status<br>Cognitive disabilities	*N* = 233; *n* = 203 male; *n* = 30 female
Baidawi and Trotter (2016) [20]	Determine the association between health, experiences of health services and environment within prisons, and psychological distress among older people incarcerated in prisons	VIC<br>NSW	Prison	Mental health status<br>Physical health status<br>Health service utilisation	*N* = 233; *n* = 203 male; *n* = 30 female
Baidawi et al. (2016) [21]	Examine the association between social experience and psychological distress among older people incarcerated in prison	VIC<br>NSW	Prison	Mental health status<br>Physical health status<br>Violence victimisation and injury	*N* = 233; *n* = 203 male; *n* = 30 female
Baidawi et al. (2016) [22]	Identify characteristics influencing distress among older people incarcerated in prisons	VIC<br>NSW	Prison	Mental health status<br>Substance dependence and substance-related harm<br>Physical health status<br>Violence victimisation and injury<br>Health service utilisation	*N* = 173; *n* = 150 male; *n* = 23 female
Bajis et al. (2019) [23]	Identify the prevalence of hepatitis C infection and liver disease, and engagement in care and treatment among people experiencing homelessness	NSW	Community<br>Prison history	Substance dependence and substance-related harm<br>Bloodborne viruses and other communicable diseases	*N* = 202; *n* = 165 male; *n* = 11 female; *n* = 2 transgender
Baldry et al. (2013) [24]	Identifying the prevalence of mental disorders and intellectual disability among people with a history of contact with the NSW justice system	NSW	Prison	Mental health status<br>Cognitive disabilities	*N* = 2731; 11.5% female

Author (year)	Study aim(s)	Location	Setting	Mapped health domains	Sample
Barling et al. (2005) [25]	Assess people incarcerated in prisons perceptions and attitudes towards healthcare and health services	NSW	Prison	Health service utilisation	*N* = 210; 97% male; 3% female
Barrett et al. (2015) [26]	Assess the acceptability, feasibility, and effectiveness of a targeted substance use and trauma prison-based program	NSW	Prison	Mental health status<br>Substance dependence and substance-related harm<br>Violence victimisation and injury	*N* = 30; 100% male
Bartholomew et al. (1967) [27]	Distinguish between offenders who present with psychiatric morbidities compared to those who do not	VIC	Prison	Mental health status<br>Substance dependence and substance-related harm<br>Physical health status<br>Non-communicable diseases	*N* = 70; 100% male
Bartlett et al. (2016) [28]	Determine factors related to clustering and genome sequences among people with recent hepatitis C infections	National	Prison<br>Other	Substance dependence and substance-related harm<br>Bloodborne viruses and other communicable diseases	*N* = 219; 69% male
Barton et al. (2017) [29]	Identify the characteristics associated with a history of suicide attempts and non-suicidal self-injury among men experiencing incarceration in prison	NSW	Prison	Mental health status<br>Substance dependence and substance-related harm	*N* = 87; 100% male
Barton et al. (2014) [30]	Explore predictors of self-harm among men incarcerated in prison	Not Reported	Prison	Mental health status	*N* = 87; 100% male
Bate et al. (2010) [31]	Determine the prevalence, relapse, and reinfection of hepatitis C among people incarcerated in prisons who have received treatment for chronic hepatitis C	SA	Prison	Bloodborne viruses and other communicable diseases<br>Non-communicable diseases<br>Health service utilisation	*N* = 74; *n* = 70 male; *n* = 4 female
Batey et al. (2008) [32]	Describe the establishment of a liver clinic service in two regional NSW prisons	NSW	Prison	Substance dependence and substance-related harm<br>Bloodborne viruses and other communicable diseases<br>Health service utilisation	*N* = 196 (sex/gender not reported)
Belcher et al. (2006) [33]	Describe prevalence and factors associated with smoking among people in prisons	NSW	Prison	Mental health status<br>Substance dependence and substance-related harm<br>Health service utilisation	*N* = 914; *n* = 747 male; *n* = 167 female
Bell et al., (2023) [34]	Examine the risk associated with incarceration and Neonatal Abstinence Syndrome for infants	WA	Prison<br>Other	Substance dependence and substance-related harm<br>Sexual and reproductive health	*N* = 708; 51.4% male infants; 47.6% female infants
Bhandari et al. (2015) [35]	Explore patterns of substance use and substance-related harm, sociodemographic characteristics, and needs among people with intellectual disability prior to release from prison	QLD	Prison	Cognitive disabilities<br>Substance dependence and substance-related harm<br>Bloodborne viruses and other communicable diseases	*N* = 1279; 96.9% male with intellectual disability; 87.1% male without intellectual disability
Bickel and Campbell (2002) [36]	Determine the prevalence of clinical disorders among young people in youth detention	TAS	Youth detention	Mental health status<br>Cognitive disabilities<br>Substance dependence and substance-related harm	*N* = 50; *n* = 43 male; *n* = 7 female
Biles (2001) [37]	Review and compare deaths that occurred in private and public prisons between 1990 and 2000	National	Prison	Mental health status<br>Physical health status	*N* = 543 (sex/gender not reported)

Author (year)	Study aim(s)	Location	Setting	Mapped health domains	Sample
Binswanger et al. (2016) [38]	Determine mortality rates among people released from prison and discern the prevalence of mental health and substance-related problems that contributed to mortality	QLD	Prison	Substance dependence and substance-related harm<br>Bloodborne viruses and other communicable diseases<br>Physical health status	*N* = 37180; *n* = 4454 female
Boonwaat et al. (2010) [39]	Evaluate the effectiveness of a prison hepatitis assessment and treatment service	NSW	Prison	Mental health status<br>Bloodborne viruses and other communicable diseases<br>Non-communicable diseases<br>Health service utilisation	*N* = 1043; *n* = 851 male; *n* = 192 female
Borschmann et al. (2014) [40]	Determine prevalence and correlates of suicidal and self-harm behaviour among young people in custody and on community orders	VIC	Youth detention	Mental health status<br>Substance dependence and substance-related harm	Youth in custody: *n* = 273; 85.7% male; 14.3% female
Borschmann et al. (2021) [41]	Compare the health and welfare outcomes of people aged under 25 years released from prisons compared to those aged older than 25 years	QLD	Prison	Mental health status<br>Substance dependence and substance-related harm<br>Health service utilisation	*N* = 1325; *n* = 1047 male; *n* = 278 female
Borschmann et al. (2020) [42]	Determine prevalence estimates for dual diagnosis for people incarcerated in prisons and the health, social, and criminal justice characteristics that may contribute to dual diagnosis; compare Australian cohort to a Brazilian cohort	QLD	Prison	Mental health status<br>Substance dependence and substance-related harm<br>Bloodborne viruses and other communicable diseases<br>Physical health status	Australian participants: *n* = 1325; *n* = 262 female
Borschmann et al. (2017) [43]	Measure the incidence and associated risk factors for emergency department contact due to self-harm after release from prison	QLD	Post-release<br>Prison	Mental health status<br>Health service utilisation	*N* = 1307; *n* = 1030 male; *n* = 277 female
Borschmann et al. (2017) [44]	Estimate the rate of ambulance attendances due to self-harm among adults released from prison	QLD	Prison	Mental health status<br>Health service utilisation	*N* = 1309; *n* = 976 male; *n* = 260 female
Borschmann et al. (2017) [45]	Measure the accuracy and predictability of self-reported histories of self-harm against medical records among people after release from prison	QLD	Post-release<br>Prison	Mental health status<br>Health service utilisation	*N* = 1315; *n* = 1037 male; *n* = 278 female
Bower et al. (2018) [46]	Determine the prevalence of fetal alcohol spectrum disorder among youth in custody	WA	Youth detention	Cognitive disabilities	*N* = 99; *n* = 93 male; *n* = 6 female
Bretaña et al. (2015) [47]	Identify prevalence and risk factors associated with hepatitis C transmission clusters among people incarcerated in prisons	NSW	Prison	Substance dependence and substance-related harm<br>Bloodborne viruses and other communicable diseases	*N* = 79; *n* = 49 male
Brömdal et al., (2023) [48]	Explore the custodial experiences and housing preferences among trans women	QLD	Post-release<br>Prison	Violence victimisation and injury	*N* = 24 (AUS sample, *n* = 4; 100% trans women)
Brothers et al., (2023) [49]	Assess the incidence of injecting-related bacterial infections associated with incarceration and opioid agonist treatment among people with opioid use disorder	NSW	Post-release<br>Prison	Substance dependence and substance-related harm<br>Health service utilisation	*N* = 7590; 35% female

Author (year)	Study aim(s)	Location	Setting	Mapped health domains	Sample
Brown and Day (2008) [50]	Explore the association between loneliness and predictors of self-harm behaviours among people on remand	SA	Prison	Mental health status	*N* = 62; 100% male
Browne et al. (2022) [51]	Determine the sociodemographic, clinical, and justice factors associated with people who are incarcerated in prisons with mental illness	NSW	Prison	Mental health status<br>Substance dependence and substance-related harm<br>Health service utilisation	*N* = 275; 100% male
Browne et al. (2023) [52]	Distinguish whether the prevalence of self-reported self-harm and suicidal behaviour, mental illness, and substance use among people incarcerated in prison has changed between years 2001, 2009, and 2015	NSW	Prison	Mental health status<br>Substance dependence and substance-related harm<br>Cognitive disabilities	*N* = 2872; *n* = 2661 male; *n* = 211 female
Burrage et al. (2021) [53]	Assessment of a cirrhosis testing method prior to treatment of hepatitis C infection	QLD	Prison<br>Other	Bloodborne viruses and other communicable diseases<br>Non-communicable diseases<br>Health service utilisation	Prison cohort: *n* = 55; 100% male
Butler et al. (2020) [54]	Explore the characteristics and predictors of frequent attendance to the emergence department among people released from custody	QLD	Post-release<br>Prison	Mental health status<br>Substance dependence and substance-related harm<br>Health service utilisation	*N* = 1307; *n* = 1030 male; *n* = 277 female
Butler et al. (2018) [55]	Determine the prevalence of suicidal and self-harm behaviour and mental disorder among adults incarcerated in prison; examine associations between demographic characteristics, mental health, justice involvement, and suicidal ideation	ACT	Prison	Mental health status<br>Substance dependence and substance-related harm	*N* = 98; 84% male; 16% female
Butler et al. (2005) [56]	Measure the prevalence estimates of mental illness among people entering and sentenced to NSW prisons	NSW	Prison	Mental health status	*N* = 1487; *n* = 1214 male; *n* = 273 female
Butler et al. (2007) [57]	Compare the mental health of Aboriginal and non-Aboriginal people experiencing incarceration in NSW prisons	NSW	Prison	Mental health status	*N* = 1470; *n* = 1208 male; *n* = 262 female
Butler et al. (2007) [58]	Compare the physical health status of people incarcerated in prisons between those with mental illness and those without	NSW	Prison	Mental health status<br>Bloodborne viruses and other communicable diseases<br>Non-communicable diseases<br>Physical health status<br>Sexual and reproductive health	*N* = 557 (sex/gender not reported)
Butler et al. (2006) [59]	Examine rates of psychiatric morbidity among people in prisons compared to the general community	NSW	Prison	Mental health status<br>Substance dependence and substance-related harm	*N* = 916; *n* = 752 male; *n* = 164 female
Butler et al. (2008) [60]	Assess the physical health status and socioeconomic background of young people in custody	NSW	Youth detention	Non-communicable diseases<br>Physical health status<br>Sexual and reproductive health<br>Health service utilisation	*N* = 242; *n* = 223 male; *n* = 19 female
Butler et al. (2007) [61]	Determine the prevalence of bloodborne viruses and risk factors associated with transmission among people entering prisons	NSW<br>QLD<br>TAS<br>WA	Prison	Substance dependence and substance-related harm<br>Bloodborne viruses and other communicable diseases	*N* = 612; *n* = 542 male

Author (year)	Study aim(s)	Location	Setting	Mapped health domains	Sample
Butler et al. (2000) [62]	Determine the prevalence of herpes simplex virus type 2 among people in prison and risk factors associated with infection	NSW	Prison	Bloodborne viruses and other communicable diseases<br>Sexual and reproductive health	*N* = 789; *n* = 657 male; *n* = 132 female
Butler et al. (2011) [63]	Determine the prevalence and patterns of co-occurring mental and substance disorder among people incarcerated in prisons	NSW	Prison	Mental health status<br>Substance dependence and substance-related harm	*N* = 1478; *n* = 1208 male; *n* = 270 female
Butler et al. (2004) [64]	Incidence of hepatitis C among people in prison	NSW	Prison	Substance dependence and substance-related harm<br>Bloodborne viruses and other communicable diseases	*N* = 90; *n* = 85 male; *n* = 5 female
Butler et al. (2004) [65]	Examine and describe the self-reported health status of people in NSW prisons	NSW	Prison	Non-communicable diseases<br>Physical health status	*N* = 914; *n* = 747 male; *n* = 167 female
Butler and Levy (1999) [66]	Determine the prevalence of Mantoux positivity and identify risk factors associated with tuberculosis among people in prison. Evaluate the risk of tuberculosis transmission within prisons	NSW	Prison	Bloodborne viruses and other communicable diseases	*N* = 789; *n* = 657 male; *n* = 132 female
Butler et al. (2003) [67]	Examine and report histories and patterns of drug use among people in prisons	NSW	Prison	Substance dependence and substance-related harm	*N* = 789; *n* = 657 male; *n* = 132 female
Butler et al. (2013) [68]	Examine the sexual experiences, behaviours, identities, and health of people incarcerated in prisons	NSW<br>QLD	Prison	Bloodborne viruses and other communicable diseases<br>Sexual and reproductive health	*N* = 2351; *n* = 2018 male; *n* = 333 females
Butler et al. (2001) [69]	Measure the prevalence and risk factors associated with syphilis among people in prisons	NSW	Prison	Bloodborne viruses and other communicable diseases<br>Physical health status<br>Sexual and reproductive health	*N* = 789; *n* = 657 male; *n* = 132 female
Butler et al. (1999) [70]	Determine the prevalence of markers for hepatitis B, C, and G and multiple exposures among people in prisons. Compare risk factors associated with hepatitis G infection with known risk factors for hepatitis B and C	NSW	Prison	Substance dependence and substance-related harm<br>Bloodborne viruses and other communicable diseases	*N* = 789; *n* = 657 male; *n* = 132 female
Butler et al. (1997) [71]	Determine the prevalence of hepatitis B and hepatitis C infection among people entering prison and examine the risk factors associated with infection	NSW	Prison	Substance dependence and substance-related harm<br>Bloodborne viruses and other communicable diseases	*N* = 408; 100% male
Butler et al. (2022) [72]	Assess the reliability of self-report and medically verified chronic illnesses among people incarcerated in prisons	NSW	Prison	Bloodborne viruses and other communicable diseases<br>Non-communicable diseases<br>Sexual and reproductive health	*N* = 2114; 100% male
Calais-Ferreira et al. (2022) [73]	Explore the characteristics associated with multimorbidity and health service utilisation among people within two years of release from prison	QLD	Prison<br>Post-release	Mental health status<br>Cognitive disabilities<br>Non-communicable diseases<br>Health service utilisation	*N* = 1046; *n* = 792 male; *n* = 254 female
Calais-Ferreira et al. (2023) [74]	Determine cause-specific mortality estimates due to chronic disease for young people with a history of contact with the justice system and explore the factors associated	QLD	Post-release<br>Youth detention<br>Other	Non-communicable diseases	*N* = 48670; *n* = 36773 male; *n* = 11897 female

Author (year)	Study aim(s)	Location	Setting	Mapped health domains	Sample
Carlton and Segrave (2011) [75]	Capture the lived experiences of survival among women released from prison and a group of support workers	VIC	Post-release<br>Prison	Mental health status<br>Substance dependence and substance-related harm<br>Violence victimisation and injury	History of incarceration: *n* = 14; 100% female
Carlton and Segrave (2014) [76]	Capture women's experiences of survival and death after release from prison	VIC	Post-release<br>Prison	Mental health status	*N* = 14; 100% female
Carrington et al., (2024) [77]	Evaluate a state-wide HIV and HCV testing program	NSW	Prison<br>Other	Substance dependence and substance-related harm<br>Bloodborne viruses and other communicable diseases<br>Heath service utilisation	*N* = 7392 (Prison cohort, *n* = 3310; *n* = 2786 male; *n* = 513 female; *n* = 11 other)
Carroll et al. (2014) [78]	Describe medication use and knowledge among people incarcerated in prisons; determine differences according to Aboriginal status and identify correlates of medication knowledge	QLD	Prison	Mental health status<br>Cognitive disabilities<br>Non-communicable diseases<br>Physical health status<br>Health service utilisation	*N* = 1231; *n* = 971 male; *n* = 167 female
Carroll et al. (2017) [79]	Identify the prevalence of general practitioner attendance among people recently released from prison	QLD	Prison	Mental health status<br>Cognitive disabilities<br>Substance dependence and substance-related harm<br>Bloodborne viruses and other communicable diseases<br>Non-communicable diseases<br>Health service utilisation	*N* = 1190; *n* = 928 male; *n* = 262 female
Carroll et al. (2016) [80]	Explore the validity of self-reported health service and medication use within 6-months after release from prison	QLD	Prison	Mental health status<br>Cognitive disabilities<br>Substance dependence and substance-related harm<br>Bloodborne viruses and other communicable diseases<br>Health service utilisation	*N* = 864; *n* = 667 male; *n* = 197 female
Carson et al. (2022) [81]	Evaluate the incidence of hepatitis C reinfection after engagement in direct-acting antiviral therapy in prisons that do not provide needle and syringe programs	NSW	Prison	Mental health status<br>Substance dependence and substance-related harm<br>Bloodborne viruses and other communicable diseases<br>Health service utilisation	*N* = 388; *n* = 207 male; *n* = 20 female
Carson et al. (2021) [82]	Evaluate the effectiveness of hepatitis C reinfection after treatment	National	Prison	Bloodborne viruses and other communicable diseases<br>Health service utilisation	*N* = 10,843<br>Prison cohort: *n* = 681 (sex/gender not reported)
Cashin et al. (2006) [83]	Assess the prevalence and profile of people with intellectual disability in NSW prisons	NSW	Prison	Cognitive disabilities	*N* = 167 (sex/gender not reported)
Charlson et al. (2021) [84]	Explore patterns and associations between incarceration and psychosis among Indigenous adults	QLD	Community<br>Prison	Mental health status	*N* = 422; *n* = 277 male; *n* = 145 female
Chong et al. (2009) [85]	Determine health-state utilities for people in prisons and examine the impact of sociodemographic characteristics and comorbidity on health-related quality of life	NSW	Prison	Mental health status<br>Substance dependence and substance-related harm<br>Bloodborne viruses and other communicable diseases<br>Non-communicable diseases<br>Physical health status	*N* = 734; *n* = 618 male

Author (year)	Study aim(s)	Location	Setting	Mapped health domains	Sample
Chowdhury et al. (2022) [86]	Identify the association between receiving a first diagnosis of psychosis in prison or in hospital on mental health service utilisation after release from prison	NSW	Post-release<br>Prison<br>Other	Mental health status<br>Substance dependence and substance-related harm<br>Health service utilisation	*N* = 25402; *n* = 13742 male; *n* = 11660 female
Chowdhury et al. (2019) [87]	Identify factors associated with receiving a first diagnosis of psychosis while incarcerated in prison	NSW	Prison	Mental health status<br>Substance dependence and substance-related harm	*N* = 38489<br>Diagnosed in prison: *n* = 659; *n* = 575 male; *n* = 84 female
Chowdhury et al. (2021) [88]	Explore the relationship between psychosis and criminal justice contact	NSW	Prison<br>Other	Mental health status<br>Health service utilisation	*N* = 86461; *n* = 48193 male; *n* = 38268 female
Clark et al. (2023) [89]	Construct a conceptual model that conveys the effect of oppression on incarceration and post-release outcomes for Black American and Indigenous Australians	QLD<br>International	Prison	Substance dependence and substance-related harm<br>Violence victimisation and injury<br>Health service utilisation	*N* = 23 (AUS sample, *n* = 3; 100% trans women)
Clarke et al. (1986) [90]	Report the findings from a prison-based fitness program	SA	Prison	Mental health status<br>Substance dependence and substance-related harm<br>Physical health status	*N* = 89; 100% male
Cockram (2005) [91]	Reports imprisonment rates and profiles of people with intellectual disability in comparison to the general prison population	WA	Prison	Cognitive disabilities	*N* = 285; *n* = 239 male; *n* = 46 female
Coffey et al. (2003) [92]	Estimate mortality among people with a history of contact with the youth justice system	VIC	Post-release<br>Prison<br>Youth detention	Mental health status<br>Substance dependence and substance-related harm<br>Bloodborne viruses and other communicable diseases<br>Non-communicable diseases<br>Physical health status<br>Violence victimisation and injury	*N* = 2849; *n* = 2621 male; *n* = 228 female
Coffey et al. (2004) [93]	Examine predictors of mortality among young people with a history of contact with youth detention centres	VIC	Youth detention	Mental health status<br>Substance dependence and substance-related harm<br>Physical health status<br>Violence victimisation and injury	*N* = 3346; *n* = 2625 male; *n* = 224 female
Coles et al. (2019) [94]	Describe and compare trends of hepatitis B and risk factors associated with transmission among people entering prisons between 2004 and 2013	QLD	Prison	Substance dependence and substance-related harm<br>Bloodborne viruses and other communicable diseases	*N* = 2223 (sex/gender not reported)
Conolly and Potter (1990) [95]	Evaluate the effectiveness of AIDs education in NSW prisons, the prevalence of HIV, and engagement in risk behaviours associated with HIV	NSW	Prison	Substance dependence and substance-related harm<br>Bloodborne viruses and other communicable diseases	*N* = 588; *n* = 443 male; *n* = 145 female
Conway et al., (2023) [96]	Estimate the prevalence of HCV RNA and factors associated with treatment uptake among people enrolled in a testing program in NSW	NSW	Prison<br>Other	Substance dependence and substance-related harm<br>Bloodborne viruses and other communicable diseases	*N* = 5960; 76% male; 23% female; 1% other

Author (year)	Study aim(s)	Location	Setting	Mapped health domains	Sample
Conway et al. (2022) [97]	Determine the effectiveness of a hepatitis C treatment engagement intervention among people who inject drugs	NSW<br>QLD<br>SA<br>WA	Community<br>Prison history	Substance dependence and substance-related harm<br>Bloodborne viruses and other communicable diseases<br>Health service utilisation	*N* = 317 (sex/gender not reported)
Copeland et al. (1998) [98]	Examine HIV-related knowledge, attitudes, and behaviours among adolescents in youth detention with a history of substance use	NSW	Youth detention	Substance dependence and substance-related harm	*N* = 166; *n* = 126 male; *n* = 40 female
Copeland et al. (2003) [99]	Investigate patterns and risk factors associated with substance use among young people in custody	NSW	Youth detention	Mental health status<br>Substance dependence and substance-related harm<br>Physical health status	*N* = 300; 90.4% male
Cossar et al. (2018) [100]	Measure the psychiatric well-being and characteristics associated among men experiencing incarceration in prison with a history of injecting drug use	VIC	Prison	Mental health status<br>Cognitive disabilities<br>Substance dependence and substance-related harm<br>Bloodborne viruses and other communicable diseases	*N* = 317; 100% male
Cossar et al. (2022) [101]	Examine the incidence of emergency department attendance following release from prison among people with a history of injecting drug use	VIC	Prison	Mental health status<br>Substance dependence and substance-related harm<br>Physical health status<br>Health service utilisation	*N* = 393; 100% male
Crissman (2019) [102]	Determine factors associated with people who died during contact with the justice system with serious mental disorders	QLD	Prison	Mental health status<br>Substance dependence and substance-related harm<br>Physical health status<br>Health service utilisation	*N* = 38; *n* = 34 male; *n* = 4 female
Crofts et al. (1997) [103]	Measure the prevalence of hepatitis A exposure among people who inject drugs that are entering prison, compared to a population of blood donors	VIC	Prison	Substance dependence and substance-related harm<br>Bloodborne viruses and other communicable diseases	*N* = 2175; *n* = 2045 male; *n* = 130 female
Crofts et al. (1993) [104]	Describe the epidemiology of hepatitis C virus infection among people who inject drugs in VIC	VIC	Community<br>Prison history	Substance dependence and substance-related harm<br>Bloodborne viruses and other communicable diseases	*N* = 303; *n* = 183 male; *n* = 120 female
Crofts et al. (1995) [105]	Examine the spread of bloodborne viruses among people entering prisons	VIC	Prison	Substance dependence and substance-related harm<br>Bloodborne viruses and other communicable diseases	*N* = 3627; *n* = 3429 male; *n* = 198 female
Crofts et al. (1996) [106]	Determine risk behaviours associated with bloodborne viruses in people incarcerated in prison with a history of injecting drug use	VIC	Prison	Substance dependence and substance-related harm<br>Bloodborne viruses and other communicable diseases	*N* = 51; 100% male
Cumming et al. (2023) [107]	Determine differences in socio-demographics and health needs between people with a history of methamphetamine use, opioid use, or both after release from prison	QLD<br>WA	Prison	Mental health status<br>Bloodborne viruses and other communicable diseases<br>Sexual and reproductive health	*N* = 1380; *n* = 1065 male; *n* = 315 female
Cumming et al. (2024) [108]	Measure the ability of a substance use screening tool in predicting substance use after release from prison	QLD	Post-release<br>Prison	Mental health status<br>Substance dependence and substance-related harm	*N* = 1065; *n* = 838 male; *n* = 227 female
Cumming et al. (2023) [109]	Examine cannabis, methamphetamine, and opioid/heroin-related hospitalisation among people released from prison	QLD<br>WA	Post-release<br>Prison	Mental health status<br>Substance dependence and substance-related harm<br>Physical health status<br>Health service utilisation	*N* = 2585; *n* = 2068 male; *n* = 517 female

Author (year)	Study aim(s)	Location	Setting	Mapped health domains	Sample
Cunningham et al. (2017) [110]	Determine trends of hepatitis C infection and risks associated among people incarcerated in prison with a history of injecting drug use	NSW	Prison	Substance dependence and substance-related harm<br>Bloodborne viruses and other communicable diseases<br>Health service utilisation	*N* = 320 participants (91 females)
Curtis et al. (2018) [111]	Examine attitudes toward take-home naloxone among males experiencing incarceration in prison with a history of injecting drug use	VIC	Prison	Substance dependence and substance-related harm<br>Health service utilisation	*N* = 377; 100% male
Curtis et al. (2023) [112]	Examine the incidence and factors associated with opioid agonist treatment disengagement within two years of release from prison	VIC	Post-release<br>Prison	Substance dependence and substance-related harm<br>Health service utilisation	*N* = 110; 100% male
Curtis et al. (2023) [113]	Report rates of ambulance and emergency department contact and the association with opioid agonist therapy among people within three months of release from prison	VIC	Post-release<br>Prison	Mental health (including self-harm and suicidal behaviour)<br>Substance dependence and substance-related harm<br>Health service utilisation	*N* = 265; 100% male
Curtis et al. (2023) [114]	Examine differences in the prevalence of healthcare and medication use among people who engaged in opioid agonist treatment after release from prison and those who did not	VIC	Prison	Mental health status<br>Substance dependence and substance-related harm<br>Non-communicable diseases<br>Physical health status<br>Health service utilisation	*N* = 255; 100% male
Cutcher et al. (2014) [115]	Examine the relationship between self-reported mental disorder diagnoses and health-related outcomes among people within 6-months of release from prison	QLD	Post-release<br>Prison	Mental health status<br>Substance dependence and substance-related harm<br>Physical health status<br>Health service utilisation	*N* = 1230; *n* = 280 female
D'Antoine et al. (2022) [116]	Determine the relationship between adverse childhood experiences and suicidal behaviours among young men in custody	SA	Youth detention	Mental health status<br>Violence victimisation and injury	*N* = 1726; 100% male
D’Souza et al. (2024) [117]	Explore the experiences of males who did and did not engage with a prison-based drug and alcohol program	NSW	Prison	Substance dependence and substance-related harm<br>Health service utilisation	*N* = 22; 100% male
D'Souza et al. (2005) [118]	Determine the prevalence of diabetes mellitus and cardiovascular disease and compare differences between a 1996 and 2001 prison cohort	NSW	Prison	Substance dependence and substance-related harm<br>Non-communicable diseases<br>Physical health status	*N* = 1805; *n* = 1,405 male; *n* = 400 female
Daley et al. (2014) [119]	Examine the prevalence of Mycoplasma genitalium and macrolide resistance among men incarcerated in Far North Queensland	QLD	Prison	Bloodborne viruses and other communicable diseases<br>Sexual and reproductive health	*N* = 140; 100% male
Dalton (1999) [120]	Review Australia-wide deaths in custody between 1980 and 1998	National	Prison	Mental health status<br>Bloodborne viruses and other communicable diseases<br>Non-communicable diseases<br>Physical health status<br>Violence victimisation and injury	*N* = 754 (sex/gender not reported)
Darke and Kaye (2004) [121]	Examine histories of lifetime and recent suicide attempts among injecting and non-injecting cocaine users in Sydney	NSW	Community<br>Prison history	Mental health status<br>Substance dependence and substance-related harm	*N* = 183; 68% male

Author (year)	Study aim(s)	Location	Setting	Mapped health domains	Sample
Darke et al. (1998) [122]	Determine the prevalence of drug use and risk behaviours among patients of a prison-based methadone maintenance program; explore the impact of antisocial personality disorder on the effectiveness of the program; compare drug use and risk behaviours with a match community sample	NSW	Prison<br>Other	Substance dependence and substance-related harm<br>Health service utilisation	Prison patients: *n* = 100; 53% male
Darke et al. (2000) [123]	Analyse and describe heroin-related deaths in NSW between 1992-1996	NSW	Prison<br>Other	Mental health status<br>Substance dependence and substance-related harm	*N* = 953; *n* = 812 male; *n* = 141 female
David et al. (2013) [124]	Investigate determinants of attrition within a cohort of people released from prison	QLD	Post-release<br>Prison	Cognitive disabilities<br>Substance dependence and substance-related harm	*N* = 921 (sex/gender not reported)
Day and Dolan (2006) [125]	Determine correlates and prevalence of hepatitis C testing among heroin users	NSW	Community<br>Prison history	Substance dependence and substance-related harm<br>Bloodborne viruses and other communicable diseases<br>Health service utilisation	*N* = 395; *n* = 249 male; *n* = 146 female
Day et al. (2003) [126]	Determine characteristics of, and differences between, Aboriginal and non-Aboriginal people who inject drugs	NSW	Community<br>Prison history	Substance dependence and substance-related harm<br>Bloodborne viruses and other communicable diseases	Aboriginal participants: *n* = 1185 (non-Aboriginal sample not described; sex/gender not reported)
de Andrade et al. (2019) [127]	Determine incidence and patterns of emergency health service contact among people released from prison and examine the association with reincarceration	QLD	Prison	Mental health status<br>Substance dependence and substance-related harm<br>Physical health status<br>Violence victimisation and injury<br>Health service utilisation	*N* = 1181 (sex/gender not reported)
Dean and Korobanova (2018) [128]	Explore whether self-reported mental illness diagnosis and treatment is preferred to assessing current symptoms among people entering prison	NSW	Prison	Mental health status	*N* = 707; *n* = 640 male; *n* = 16 female
Dean et al. (2023) [129]	Compare characteristics of justice contact between First Nations and non-First Nations forensic patients	NSW	Post-release<br>Prison	Mental health status<br>Cognitive disabilities<br>Substance dependence and substance-related harm<br>Violence victimisation and injury	*N* = 477; *n* = 411 male; *n* = 64 female)
Dear et al. (2001) [130]	Examine the extent and nature of non-fatal self-harm behaviour among people in prison	WA	Prison	Mental health status<br>Substance dependence and substance-related harm<br>Health service utilisation	*N* = 91; *n* = 79 male; *n* = 12 female
Dear et al. (2001) [131]	Assess characteristics associated with people in prisons who self-harm compared to people who have not	WA	Prison	Mental health status<br>Substance dependence and substance-related harm<br>Violence victimisation and injury	*N* = 71; *n* = 64 male; *n* = 7 female
Degenhardt et al. (2014) [132]	Examine the impact of opioid substitution therapy on opioid dependence during incarceration and mortality after release	NSW	Post-release<br>Prison	Mental health status<br>Substance dependence and substance-related harm<br>Violence victimisation and injury<br>Health service utilisation	*N* = 16453; *n* = 12,945 male

Author (year)	Study aim(s)	Location	Setting	Mapped health domains	Sample
Degenhardt et al. (2015) [133]	Examine the prevalence and characteristics associated with justice contact in a sample of young people; explore associations between substance use, problematic substance use, and psychotic symptoms	VIC	Youth detention<br>Other	Mental health status<br>Substance dependence and substance-related harm<br>Violence victimisation and injury	N = 515; 82.7% male<br>Custodial sample: n = 273
Degenhardt et al. (2014) [134]	Measure the prevalence, period of incarceration, and costs associated with incarceration for people with opioid dependence using data linkage	NSW	Prison<br>Youth detention	Substance dependence and substance-related harm	N = 47196; n = 31623 male; n = 15573 female
Dellar et al. (2023) [135]	Examine the reliability and validity of the YLS/CMI using a sample of justice-involved young people	WA	Youth detention	Cognitive disabilities<br>Substance dependence and substance-related harm	N = 4653<br>Custodial sample: n = 238; n = 212 male; n = 26 female
Denton (1995) [136]	Measure the prevalence of severe mental disorder and substance dependence among women incarcerated in prison	VIC	Prison	Mental health status<br>Substance dependence and substance-related harm	N = 56; 100% female
Denton et al. (2017) [137]	Explore the experiences of mental health and criminal justice systems among people recently released from prison	QLD	Post-release<br>Prison	Mental health status<br>Substance dependence and substance-related harm<br>Health service utilisation	N = 18; 100% male
Dias et al. (2018) [138]	Investigate if employment after release from prison is associated lower risk of reincarceration	QLD	Post-release<br>Prison	Mental health status<br>Substance dependence and substance-related harm	N = 774; n = 600 male; n = 174 female
Dias et al. (2013) [139]	Determine health and health-related characteristics of people incarcerated in prisons with possible intellectual disability	QLD	Prison	Cognitive disabilities<br>Bloodborne viruses and other communicable diseases<br>Non-communicable diseases<br>Physical health status<br>Sexual and reproductive health<br>Health service utilisation	N = 1279; n = 1005 male
Dias et al. (2013) [140]	Examine the prevalence of co-morbid mental disorders for people incarcerated in prison with and without intellectual disability; explore the relationship between mental disorder and intellectual disability	QLD	Prison	Mental health status<br>Cognitive disabilities<br>Substance dependence and substance-related harm<br>Health service utilisation	N = 1279; n = 1005 male
Dixon et al. (2004) [141]	Report current and lifetime psychological disorders among young females in custody and associations between mental health status, experiences of trauma, and sociodemographic characteristics	NSW	Youth detention	Mental health status<br>Cognitive disabilities<br>Violence victimisation and injury	N = 100; 100% female
Dolan et al. (2003) [142]	Determine the impact of methadone maintenance treatment on heroin use, syringe sharing, HIV, or hepatitis C among a prison population	NSW	Prison	Substance dependence and substance-related harm<br>Bloodborne viruses and other communicable diseases<br>Health service utilisation	N = 253; 100% male
Dolan et al. (2005) [143]	Explore the long-term impact of engagement in methadone maintenance treatment on mortality, reincarceration, and hepatitis C infection among males who use heroin	NSW	Prison	Substance dependence and substance-related harm<br>Bloodborne viruses and other communicable diseases<br>Health service utilisation	N = 382; 100% male

Author (year)	Study aim(s)	Location	Setting	Mapped health domains	Sample
Dolan et al. (2015) [144]	Determine the prevalence of substance use problems among Indigenous and non-Indigenous people experiencing incarceration; identify the availability of treatment programs within prisons	National	Prison	Substance dependence and substance-related harm<br>Health service utilisation	Sample size and characteristics not reported
Dolan et al. (2010) [145]	Investigate the incidence and associated predictors of hepatitis C infection among seronegative people who inject drugs	NSW	Prison	Substance dependence and substance-related harm<br>Health service utilisation	*N* = 120; *n* = 98 male
Dominguez D et al. (2022) [146]	Identify the factors associated with traumatic brain injury during the COVID-19 pandemic	VIC	Prison	Violence victimisation and injury<br>Health service utilisation	Prison cohort: *n* = 221 prison cohort; *n* = 211 male; *n* = 10 female
Doolan et al. (2012) [147]	Determine the health needs of Indigenous and non-Indigenous young people entering custody	QLD	Youth detention	Mental health status<br>Substance dependence and substance-related harm<br>Health service utilisation	*N* = 1755; 75% Indigenous male; 25% Indigenous female; 81% non-Indigenous male; 19% non-Indigenous female
Dowell et al. (2018) [148]	Examine determinants of infant mortality among people incarcerated or with a history of incarceration	WA	Prison	Substance dependence and substance-related harm<br>Sexual and reproductive health<br>Health service utilisation	*N* = 546; *n* = 304 male; *n* = 242 female
Dowell et al. (2019) [149]	Explore the effect of maternal imprisonment during pregnancy on low birth weight for infants in WA	WA	Prison	Mental health status<br>Substance dependence and substance-related harm<br>Non-communicable diseases<br>Sexual and reproductive health<br>Health service utilisation	*N* = 22363; 100% female
Doyle et al. (2023) [150]	Evaluate associations between alcohol use, smoking, and illicit drug use among people incarcerated in prisons	NSW	Prison	Substance dependence and substance-related harm	*N* = 1132; *n* = 757 male; *n* = 375 females
Doyle et al. (2015) [151]	Determine levels of prior alcohol and other drug use among Indigenous and non-Indigenous people entering prisons	NSW	Prison	Mental health status<br>Cognitive disabilities<br>Substance dependence and substance-related harm	*N* = 200; 100% male
Doyle et al. (2020) [152]	Examine substance use prior to and during incarceration for Aboriginal men engaging in a prison-based treatment intervention	NSW	Prison	Substance dependence and substance-related harm<br>Health service utilisation	*N* = 14; 100% male
Draper (2023) [153]	Explore the patterns of attempted suicide among older people in NSW between 1870 and 1908	NSW	Prison<br>Other	Mental health status	Prison cohort: *n* = 255; *n* = 214 male; *n* = 41 female
Drew (1961) [154]	Investigate the psychiatric profiles of people who have been charged with alcohol-related offenses	VIC	Prison	Substance dependence and substance-related harm<br>Physical health status (including exercise and obesity)	*N* = 178; 100% male
Dunlop et al. (2022) [155]	Evaluate the safety of depot buprenorphine treatment for opioid dependence among people incarcerated in prison	NSW	Prison	Substance dependence and substance-related harm<br>Bloodborne viruses and other communicable diseases<br>Health service utilisation	*N* = 129; *n* = 108 male; *n* = 21 female

Author (year)	Study aim(s)	Location	Setting	Mapped health domains	Sample
Eckstein et al. (2007) [156]	Examine the health and characteristics of people incarcerated in NSW prisons and trends over time	NSW	Prison	Mental health status<br>Physical health status	2001 cohort: *n* = 914; 32% female
Edwards et al. (2024) [157]	Examine associations between social determinants of health, mental health, substance use, and justice contact among women released from prison with a history of substance use	NSW	Post-release<br>Prison	Mental health status<br>Substance dependence and substance-related harm<br>Violence victimisation and injury<br>Health service utilisation	*N* = 413; 100% female
Egeressy et al. (2009) [158]	Explore the association between post-traumatic stress disorder and personality among people incarcerated in prisons	NSW	Prison	Mental health status<br>Violence victimisation and injury	*N* = 1437; *n* = 1170 male; *n* = 267 female
Ellem (2012) [159]	Examine the lived experience of people with intellectual disability on reintegrating into community after release from prison	QLD	Post-release<br>Prison	Cognitive disabilities	*N* = 10; *n* = 7 male; *n* = 3 female
Ellem et al. (2012) [160]	Describe the lived experiences and needs of people with intellectual disability who have a history of incarceration	QLD	Post-release<br>Prison	Cognitive disabilities<br>Health service utilisation	*N* = 10; *n* = 7 male; *n* = 3 female
Eriksson et al. (2021) [161]	Examine substance use among people one year prior to their homicide offence and compare their sociodemographic, developmental, justice history, and personality characteristics	National	Prison	Substance dependence and substance-related harm<br>Violence victimisation and injury	*N* = 302; 86.4% male; 13.6% female
Eriksson et al. (2023) [162]	Explore victim-offender relationships and gender associations among men who commit homicide	National	Prison	Substance dependence and substance-related harm<br>Violence victimisation and injury	*N* = 247; 100% male
Fairley et al. (1990) [163]	Measure the prevalence of hepatitis C across various groups of people who are at higher risk of exposure	VIC	Prison<br>Other	Bloodborne viruses and other communicable diseases	Prison cohort: *n* = 149 (sex/gender not reported)
Falster et al. (2009) [164]	Explore trends in hepatitis C infection among needle and syringe program participants between 1995 and 2004	National	Community<br>Prison history	Substance dependence and substance-related harm<br>Bloodborne viruses and other communicable diseases	*N* = 12715; *n* = 8358 male; *n* = 4307 female; *n* = 50 transgender
Fasher et al. (1997) [165]	Explore the health profiles of young people entering youth detention	NSW	Youth detention	Mental health status<br>Substance dependence and substance-related harm<br>Bloodborne viruses and other communicable diseases<br>Non-communicable diseases<br>Physical health status<br>Violence victimisation and injury	*N* = 100; *n* = 97 male; *n* = 3 female
Fazel et al. (2011) [166]	Contrast rates of prison suicide in 12 countries between 2003 and 2007	National	Prison	Mental health status	Australian cohort: *n* = 69; 100% male
Field (2018) [167]	Identify correlates and predictors of alcohol misuse among non-Aboriginal people incarcerated in prison	NSW	Prison	Substance dependence and substance-related harm	*N* = 283; 100% male
Field and Archer (2019) [168]	Compare and describe the health status, disability, and service accessibility for younger and older people incarcerated in prisons	NSW	Prison	Non-communicable diseases<br>Physical health status<br>Health service utilisation	*N* = 1132; *n* = 756 male; *n* = 375 female

Author (year)	Study aim(s)	Location	Setting	Mapped health domains	Sample
Field et al. (2020) [169]	Compare the prevalence of substance use, diet, and exercise between Australian-born and immigrants experiencing incarceration in prison and determine predictors of chronic illness	NSW	Prison	Substance dependence and substance-related harm<br>Bloodborne viruses and other communicable diseases<br>Non-communicable diseases<br>Physical health status	*N* = 566; *n* = 370 male; *n* = 196 female
Fleming et al. (2012) [170]	Examine the findings of a prison-based mental health pilot project	WA	Prison	Mental health status<br>Substance dependence and substance-related harm<br>Health service utilisation	*N* = 146; *n* = 91 male; *n* = 55 female
Fleming et al. (2001) [171]	Describe childhood sexual abuse experienced by people in prisons and explore associated mental health outcomes and risk behaviours	NSW	Prison	Mental health status<br>Substance dependence and substance-related harm<br>Sexual and reproductive health<br>Violence victimisation and injury	*N* = 789; *n* = 657 male; *n* = 132 female
Forrest et al. (2009) [172]	Report the findings and cost-effectiveness of chlamydia screening in prisons	NSW	Prison	Sexual and reproductive health	*N* = 3209 (sex/gender not reported)
Forsyth et al. (2023) [173]	Determine the impact of asthma on mortality for people released from prison	QLD	Post-release<br>Prison	Mental health status<br>Cognitive disabilities<br>Physical health status	*N* = 42015; *n* = 37039 male; *n* = 4976 female
Forsyth et al. (2014) [174]	Examine the incidence, timing, and risk factors associated with substance-related deaths, and compare differences within Indigenous status, among people released from prison	QLD	Post-release<br>Prison	Substance dependence and substance-related harm<br>Physical health status	*N* = 42015; *n* = 37039 male
Forsyth et al. (2018) [175]	Determine the incidence and associated risk factors for mortality among people released from prison	QLD	Prison	Mental health status<br>Cognitive disabilities<br>Substance dependence and substance-related harm<br>Bloodborne viruses and other communicable diseases<br>Non-communicable diseases<br>Physical health status	*N* = 1320; *n* = 1041 male
Garner et al. (1997) [176]	Estimate the prevalence of hepatitis C infection and associated risk factors among a group of pregnant women	SA	Prison	Substance dependence and substance-related harm<br>Bloodborne viruses and other communicable diseases	*N* = 1537; 100% female
Gates et al. (2004) [177]	Compare the prevalence of risk factors associated with hepatitis C infection among men in prison	NSW	Prison	Substance dependence and substance-related harm<br>Bloodborne viruses and other communicable diseases	*N* = 121; 100% male
Gaughwin and Ali (1995) [178]	Investigate risk factors associated with HIV infection among people who inject drugs	SA	Community<br>Prison history	Bloodborne viruses and other communicable diseases	*N* = 269 (sex/gender not reported)
Gaughwin et al. (1991) [179]	Report the prevalence of HIV in SA prisons and estimate the prevalence and frequency of risk behaviours associated with HIV transmission	SA	Post-release<br>Prison	Substance dependence and substance-related harm<br>Bloodborne viruses and other communicable diseases	*N* = 373 (sex/gender not reported)
Gibbs et al. (2024) [180]	Determine the feasibility of linking administrative data to assess attendance to supported accommodation and associations with health and justice contact; compare rates of health and justice contact; evaluate impact of program participation	NSW	Post-release<br>Prison	Physical health status<br>Health service utilisation	*N* = 415; 100% male

Author (year)	Study aim(s)	Location	Setting	Mapped health domains	Sample
Gibbs et al. (2021) [181]	Determine the trajectory of hepatitis C testing and treatment among people who inject drugs and identify factors associated with treatment utilisation	NSW<br>QLD<br>VIC<br>TAS<br>SA<br>WA<br>NT	Community<br>Prison history	Substance dependence and substance-related harm<br>Bloodborne viruses and other communicable diseases<br>Health service utilisation	*N* = 1499; *n* = 1008 male; *n* = 490 female
Gidding et al. (2015) [182]	Determine hepatitis B immunity status among people incarcerated in prison compared with the general population	NSW<br>QLD<br>SA<br>TAS<br>WA<br>VIC<br>ACT	Prison	Bloodborne viruses and other communicable diseases<br>Health service utilisation	*N* = 531; 90% male; 10% female
Gilchrist et al. (2022) [183]	Investigate the characteristics associated with health and social service utilisation and accessibility for young people released from prison	NSW	Prison	Mental health status<br>Substance dependence and substance-related harm<br>Physical health status<br>Health service utilisation	*N* = 359; *n* = 279 male; *n* = 80 female
Gilles et al. (2008) [184]	Investigate the response to prison and public health in a regional WA prison	WA	Prison	Substance dependence and substance-related harm<br>Bloodborne viruses and other communicable diseases<br>Non-communicable diseases<br>Sexual and reproductive health<br>Health service utilisation	*N* = 185; 92% male; 8% female
Gisev et al. (2014) [185]	Compare justice involvement and opioid substitution therapy utilisation among Indigenous and non-Indigenous people who are opioid dependent	NSW	Post-release<br>Prison	Substance dependence and substance-related harm	*N* = 34962; *n* = 24794 male; *n* = 10168 female
Glaser (1985) [186]	Examine the characteristics that differentiate between people experiencing incarceration with mental illness and those without	VIC	Prison	Mental health status<br>Cognitive disabilities<br>Substance dependence and substance-related harm	*N* = 50 (sex/gender not reported)
Glaser and Deane (1999) [187]	Explore the characteristics and experiences of people with intellectual disability incarcerated in prison, compared to an institution group	VIC	Prison<br>Other	Cognitive disabilities<br>Substance dependence and substance-related harm<br>Violence victimisation and injury<br>Health service utilisation	Prison cohort: *n* = 109; 100% male
Glaser and Laster (1990) [188]	Examine the impact of the 1986 Mental Health Act (VIC) on people incarcerated in prisons and admitted to mental hospitals	VIC	Prison	Mental health status<br>Health service utilisation	*N* = 226; *n* = 207 male; *n* = 19 female
Gordon (1963) [189]	Historical account of disease and mortality in the Moreton Bay convict settlement between 1824 and 1837	QLD	Prison	Bloodborne viruses and other communicable diseases (excluding STIs)<br>Violence victimisation and injury<br>Health service utilisation	*N* = 2403; *n* = 2259 male; *n* = 144 female
Goulter et al. (2018) [190]	Evaluate the psychometric properties and utility of an antisocial process screening tool among young people in custody	NSW	Youth detention	Mental health status<br>Substance dependence and substance-related harm	*N* = 361; 100% male

Author (year)	Study aim(s)	Location	Setting	Mapped health domains	Sample
Gower et al. (2023) [191]	Use the LS/RNR and VRS risk assessment tools to examine the needs and criminogenic profile of violent Aboriginal offenders and compare to violent non-Aboriginal offenders	WA	Prison	Substance dependence and substance-related harm	*N* = 3695; *n* = 3483 male; *n* = 482 female
Gower et al. (2023) [192]	Use the LS/RNR and VRS risk assessment tools to explore the criminogenic profiles of violent female offenders	WA	Prison	Mental health status<br>Substance dependence and substance-related harm	*N* = 1704; *n* = 1547 male; *n* = 157 female
Graffam and Shinkfield (2012) [193]	Explore the life conditions of people released from prison	Not reported	Post-release<br>Prison	Mental health status<br>Substance dependence and substance-related harm<br>Bloodborne viruses and other communicable diseases<br>Non-communicable diseases	*N* = 36; *n* = 20 male; *n* = 16 female
Graham (2003) [194]	Examine the nature and extent of unnatural death among people released from prison between 1990 and 1999	VIC	Post-release<br>Prison	Substance dependence and substance-related harm<br>Physical health status	*N* = 25469; *n* = 22978 male; *n* = 2490 female
Green et al. (2016) [195]	Report findings from a mental health transitional support program and the characteristics of the people that utilised the service	QLD	Post-release<br>Prison	Mental health status<br>Substance dependence and substance-related harm<br>Health service utilisation	*N* = 63; *n* = 55 male
Gullotta et al. (2023) [196]	Examine the physical health status and compare between people convicted of sexual offences and people without a history of sexual offending	NSW	Prison	Bloodborne viruses and other communicable diseases<br>Non-communicable diseases<br>Physical health status	*N* = 2114; 100% male
Gullotta et al. (2020) [197]	Classify sex offenders among participants in the NSW Inmate Health Survey and compare demographic characteristics, health profiles, and criminal histories to non-sex offenders	NSW	Prison	Mental health status<br>Substance dependence and substance-related harm<br>Non-communicable diseases<br>Physical health status	*N* = 283; 100% male
Gullotta et al. (2021) [198]	Determine the prevalence, predicators, and characteristics associated with suicidal and self-harm behaviours among groups of sex offenders	NSW	Prison	Mental health status<br>Cognitive disabilities<br>Substance dependence and substance-related harm<br>Health service utilisation	*N* = 2114; 100% male
Guthrie et al. (2012) [199]	Describe influenza control measures used during an outbreak in 2009 and 2011	ACT	Prison	Substance dependence and substance-related harm<br>Bloodborne viruses and other infectious diseases<br>Non-communicable diseases<br>Physical health status	Sample size and characteristics not reported
Haber et al. (1999) [200]	Describe cases and transmission of hepatitis C infection in prisons	NSW	Prison	Mental health status<br>Substance dependence and substance-related harm<br>Bloodborne viruses and other communicable diseases<br>Violence victimisation and injury	*N* = 4; 100% male
Hail-Jares et al. (2023) [201]	Identify the relationship between sexuality, suicide and self-harm behaviour, and criminal justice involvement among people incarcerated in prison	QLD<br>WA	Prison	Mental health status<br>Substance dependence and substance-related harm	*N* = 2698; *n* = 2168 male; *n* = 530 female
Hajarizadeh et al. (2024) [202]	Evaluate the incidence of HCV infection and characteristics associated with injecting drug use in the prison setting	NSW	Prison	Substance dependence and substance-related harm<br>Bloodborne viruses and other communicable diseases<br>Health service utilisation	*N* = 3293; *n* = 2669 male; *n* = 622 female

Author (year)	Study aim(s)	Location	Setting	Mapped health domains	Sample
Hajarizadeh et al. (2021) [203]	Assess the effectiveness of hepatitis C treatment on transmission within prisons	NSW	Prison	Bloodborne viruses and other communicable diseases<br>Non-communicable diseases<br>Health service utilisation	*N* = 3691; *n* = 3010 male; *n* = 679 female; *n* = 2 transgender
Halder et al. (2021) [204]	Determine the effectiveness and feasibility of a telehealth model of care for people incarcerated in prison with hepatitis C	WA	Prison	Bloodborne viruses and other communicable diseases<br>Non-communicable diseases<br>Health service utilisation	*N* = 332; *n* = 266 male; *n* = female
Hamilton et al. (2020) [205]	Describe the experiences of assessing fetal alcohol spectrum disorder for young people in custody and consider the impact of assessment on participants	WA	Youth detention	Cognitive disabilities	*N* = 38 (sex/gender not reported)
Hampton et al. (2021) [206]	Evaluate the NSW prison response to COVID-19 by comparing to the response to pandemics that occurred in 1860 and 1919	NSW	Prison	Bloodborne viruses and other communicable diseases	Sample size and characteristics not reported
Hando et al. (1997) [207]	Examine correlations between demographic characteristics, substance use, and offending among youth in detention	NSW	Youth detention	Substance dependence and substance-related harm<br>Health service utilisation	*N* = 279; 95.7% male
Hannan-Jones and Capra (2016) [208]	Assess the prevalence of markers for non-communicable disease among males incarcerated in prison	QLD	Prison	Mental health status<br>Substance dependence and substance-related harm<br>Non-communicable diseases<br>Physical health status	*N* = 121; 100% male
Harman and Maxwell-Stewart (2012) [209]	Review Aboriginal deaths in custody at Cockatoo Island between 1805 and 1860 and describe factors associated with mortality	NSW	Prison<br>Other	Physical health status	*N* = 60; 100% male
Haysom et al. (2020) [210]	Investigate the incidence of neutropenia among young men in custody	NSW	Youth detention	Mental health status<br>Cognitive disabilities<br>Physical health status<br>Health service utilisation	*N* = 6; 100% male
Haysom et al. (2019) [211]	Determine the incidence, characteristics, and risk factors for soft tissue infections among youth in detention	NSW	Youth detention	Bloodborne viruses and other communicable diseases	*N* = 77; *n* = 71 male; *n* = 6 female
Haysom et al. (2015) [212]	Measure the prevalence and risk factors associated with poor oral health outcomes among Aboriginal and non-Aboriginal youth in detention	NSW	Youth detention	Substance dependence and substance-related harm<br>Physical health status<br>Health service utilisation	*N* = 382; *n* = 268 male; *n* = 26 female
Haysom et al. (2014) [213]	Identify the prevalence of possible and borderline intellectual functioning among young people in detention and explore the association between intellectual disability, social disadvantage, and Aboriginal status	NSW	Youth detention	Mental health status<br>Cognitive disabilities	*N* = 361; *n* = 257 male; *n* = 38 female
Haysom et al. (2013) [214]	Measure the prevalence and risk factors associated with overweight, obesity, and perceptions of weight gain among young people in detention and 12-months post-release	NSW	Post-release<br>Youth detention	Mental health status<br>Substance dependence and substance-related harm<br>Physical health status<br>Health service utilisation	*N* = 303; *n* = 264 male

Author (year)	Study aim(s)	Location	Setting	Mapped health domains	Sample
Haysom et al. (2017) [215]	Determine the prevalence of nicotine dependence and attitudes towards nicotine replacement therapy among youth entering detention	NSW	Youth detention	Substance dependence and substance-related harm<br>Health service utilisation	*N* = 252; *n* = 227 male; *n* = 25 female
Heffernan et al. (2012) [216]	Determine the prevalence of mental disorder among Aboriginal and Torres Strait Islander people incarcerated in prisons	QLD	Prison	Mental health status<br>Substance dependence and substance-related harm	*N* = 347; *n* = 276 male; *n* = 72 female
Heffernan et al. (2016) [217]	Determine the prevalence and mental health risk factors associated with substance dependence among Indigenous people experiencing incarceration in prison	QLD	Prison	Mental health status<br>Substance dependence and substance-related harm<br>Health service utilisation	*N* = 396; *n* = 331 male; *n* = 65 female
Heffernan et al. (2015) [218]	Determine demographic, history of trauma, and justice involvement correlates of post-traumatic stress disorder among Indigenous people who are experiencing incarceration	QLD	Prison	Mental health status<br>Substance dependence and substance-related harm<br>Violence victimisation and injury	*N* = 396; *n* = 331 male; *n* = 65 female
Hellard et al. (2004) [219]	Measure the prevalence and risk factors associated with hepatitis C infection among people in prison	VIC	Prison	Substance dependence and substance-related harm<br>Bloodborne viruses and other communicable diseases	*N* = 642; *n* = 518 male; *n* = 124 female
Henderson and Bull (2023) [220]	Examine the sentencing outcomes for people with cognitive disability	QLD	Prison	Mental health status<br>Cognitive disabilities<br>Substance dependence and substance-related harm<br>Violence victimisation and injury	*N* = 34; *n* = 23 male; *n* = 8 female; *n* = 3 sex/gender not reported
Herrman et al. (1991) [221]	Estimate the prevalence of severe mental disorders in a representative sample of people incarcerated in prisons	VIC	Prison	Mental health status<br>Substance dependence and substance-related harm	*N* = 189; *n* = 158 male; *n* = 31 female
Herrman et al. (1994) [222]	Examine contact with psychiatric services prior to incarceration for people not receiving psychiatric care while in prison	VIC	Prison	Mental health status<br>Cognitive disabilities<br>Substance dependence and substance-related harm<br>Health service utilisation	*N* = 189; *n* = 158 male; *n* = 31 female
Hesse et al. (2023) [223]	Examine the outcomes of a bowel and breast cancer screening program for people incarcerated in prison	QLD	Prison	Non-communicable diseases	*N* = 164 (sex/gender not reported)
Hilder et al. (2017) [224]	Explore the pregnancy outcomes of women who experience pregnancy during incarceration	NSW	Prison	Mental health status<br>Substance dependence and substance-related harm<br>Sexual and reproductive health<br>Health service utilisation	*N* = 624; 100% female
Hill et al. (2022) [225]	Determine mortality rates and risk factors associated with death among people who inject drugs	VIC	Community<br>Prison history	Substance dependence and substance-related harm<br>Physical health status	*N* = 1209; *n* = 813 male; *n* = 396 female
Hobbs et al. (2006) [226]	Understand the continuity of health problems and service needs among people released from prison	WA	Post-release<br>Prison	Mental health status<br>Substance dependence and substance-related harm<br>Bloodborne viruses and other communicable diseases<br>Non-communicable diseases<br>Violence victimisation and injury<br>Health service utilisation	*N* = 13667; 88.3% male; 11.7% female

Author (year)	Study aim(s)	Location	Setting	Mapped health domains	Sample
Hobday et al. (2023) [227]	Determine the prevalence and factors associated with current hepatitis C infection and history of treatment among Aboriginal people who inject drugs	NSW<br>QLD<br>SA<br>WA	Community<br>Prison history	Substance dependence and substance-related harm<br>Health service utilisation	*N* = 2395<br>Prison history: *n* = 1624; *n* = 1591 male; *n* = 786 female; *n* = 18 transgender/other
Holland and Persson (2011) [228]	Explore the characteristics of people with intellectual disability who are released from prison in VIC	VIC	Post-release<br>Prison	Mental health status<br>Cognitive disabilities<br>Health service utilisation	*N* = 346; 100% male
Holmwood et al. (2008) [229]	Examine patterns of substance use among prisoners entering SA prisons using the WHO-ASSIST screener	SA	Prison	Substance dependence and substance-related harm	*N* = 518; *n* = 446 male; *n* = 72 female
Howard et al. (2003) [230]	Determine the prevalence and predictors of suicide and self-harm behaviours among young people in custody	NSW	Youth detention	Mental health status<br>Substance dependence and substance-related harm<br>Violence victimisation and injury	*N* = 299; *n* = 270 male; *n* = 29 female
Howard et al. (2020) [231]	Identify individual and facility factors associated with assaults among people incarcerated in prisons	NSW	Prison	Mental health status<br>Violence victimisation and injury	*N* = 10484; *n* = 685 female
Howard and Zibert (1990) [232]	Assess whether patterns of substance use differ between young people in custody and non-justice-involved adolescents	NSW	Youth detention	Substance dependence and substance-related harm	*N* = 292; *n* = 263 male; *n* = 29 female
Hurley (1989) [233]	Report the criminological and psychiatric characteristics of males who committed suicide in a Brisbane prison between 1973 and 1987	QLD	Prison	Mental health status<br>Substance dependence and substance-related harm<br>Non-communicable diseases	*N* = 44; 100% male
Hurley and Dunne (1991) [234]	Measure the prevalence of psychological distress and psychiatric morbidity of women incarcerated in prison	QLD	Prison	Mental health status<br>Substance dependence and substance-related harm<br>Health service utilisation	*N* = 92; 100% female
Indermaur and Upton (1988) [235]	Examine the patterns of alcohol and drug use in people incarcerated in prisons	WA	Prison	Substance dependence and substance-related harm	*N* = 926; *n* = 840 male; *n* = 86 female
Indig and Haysom (2012) [236]	Determine the predictors and prevalence of smoking among youth in detention	NSW	Youth detention	Mental health status<br>Substance dependence and substance-related harm	*N* = 316; *n* = 277 male; *n* = 39 female
Indig et al. (2016) [237]	Determine the prevalence and correlates of incarceration history and recidivism among young people in custody	NSW	Post-release<br>Youth detention	Mental health status<br>Cognitive disabilities<br>Substance dependence and substance-related harm	*N* = 319; 87.2% male
Islam et al. (2013) [238]	Identify and assess patterns of health service utilisation and the associations of demographics and drug use with emergency presentations	National	Community<br>Prison history	Substance dependence and substance-related harm<br>Health service utilisation	*N* = 2395; 67% male; 33% female; <1% transgender
Jama-Alol et al. (2015) [239]	Determine how offence type, history of imprisonment, and sociodemographic characteristics influence mortality between one month and a year post-release	WA	Post-release<br>Prison	Physical health status	*N* = 12677; *n* = 1005 male; *n* = 1672 female

Author (year)	Study aim(s)	Location	Setting	Mapped health domains	Sample
Janca et al. (2023) [240]	Explore sex differences within patterns of health service contact among people released from prison	QLD	Post-release Prison	Mental health status<br>Substance dependence and substance-related harm<br>Bloodborne viruses and other communicable diseases<br>Physical health status<br>Health service utilisation	*N* = 1307; *n* = 1030 males; *n* = 277 female
Jin et al. (2021) [241]	Determine the effectiveness of a motivational interview on smoking cessation after release from smoke-free prisons	NT	Post-release Prison	Substance dependence and substance-related harm	*N* = 557; *n* = 510 male; *n* = 47 female
Johnson (2006) [242]	Explore correlates of illicit substance use among women prior to incarceration	QLD<br>SA<br>NT<br>TAS<br>WA<br>VIC	Prison	Mental health status<br>Substance dependence and substance-related harm<br>Violence victimisation and injury	*N* = 470; 100% female
Jones et al. (1995) [243]	Describe the psychiatric, social, and criminological characteristics of females incarcerated in prison between 1981 and 1990	TAS	Prison	Mental health status<br>Substance dependence and substance-related harm	*N* = 210; 100% female
Jones et al. (2022) [244]	Compare patterns between non-fatal overdose and fatal overdose among people with a history of opioid dependency and explore the association with treatment engagement	NSW	Community Prison history	Substance dependence and substance-related harm<br>Health service utilisation	*N* = 45665; *n* = 30892 male; *n* = 14770 female
Kariminia et al. (2007) [245]	Describe standardised cause-specific mortality rates and trends among people who have experienced incarceration in prison between 1988 and 2002	NSW	Prison	Mental health status<br>Substance dependence and substance-related harm<br>Bloodborne viruses and other communicable diseases<br>Non-communicable diseases<br>Physical health status<br>Violence victimisation and injury	*N* = 87203; *n* = 78383 male; *n* = 8820 female
Kariminia et al. (2007) [246]	Compare Aboriginal and non-Aboriginal people experiencing incarceration's self-reported mental and physical health	NSW	Prison	Mental health status<br>Non-communicable diseases<br>Physical health status<br>Health service utilisation	*N* = 914; *n* = 747 male; *n* = 167 female
Kariminia et al. (2005) [247]	Evaluate the Australian National Death Index's accuracy in determining the mortality of people in NSW prisons	NSW	Prison	Mental health status<br>Substance dependence and substance-related harm<br>Non-communicable diseases<br>Physical health status<br>Violence victimisation and injury	*N* = 7869 (sex/gender not reported)
Kariminia et al. (2012) [248]	Describe cause-specific mortality among Indigenous people who have experienced incarceration in prisons	NSW	Prison	Mental health status<br>Bloodborne viruses and other infectious diseases<br>Non-communicable diseases<br>Physical health status<br>Violence victimisation and injury	*N* = 9353; *n* = 7980 male; *n* = 1373 female
Kariminia et al. (2007) [249]	Determine risk factors that increase mortality among people incarcerated in prisons	NSW	Prison	Mental health status<br>Substance dependence and substance-related harm<br>Physical health status<br>Health service utilisation	*N* = 87203; *n* = 78383 male; *n* = 8820 female

Author (year)	Study aim(s)	Location	Setting	Mapped health domains	Sample
Kariminia et al. (2007) [250]	Determine the risk of suicide and drug overdose among people released from prisons	NSW	Post-release<br>Prison	Mental health status<br>Substance dependence and substance-related harm	N = 87203; n = 76376 male; n = 8820 female
Kasinathan (2015) [251]	Explore characteristics of mental illness among young people released from custody and identify predictors of re-incarceration	NSW	Post-release<br>Youth detention	Mental health status<br>Cognitive disabilities<br>Substance dependence and substance-related harm<br>Violence victimisation and injury<br>Health service utilisation	N = 51; 100% male
Kasinathan et al. (2021) [252]	Determine the effectiveness of collaboration between governing bodies in response to the COVID-19 pandemic on self-harm among young people in custody	NSW	Youth detention	Mental health status	Sample size and characteristics not reported
Kavanagh et al. (2010) [253]	Assess the utility of a computerised psychological assessment for people incarcerated in prisons	NSW	Prison	Mental health status<br>Cognitive disabilities<br>Substance dependence and substance-related harm<br>Bloodborne viruses and other communicable diseases<br>Non-communicable diseases<br>Violence victimisation and injury<br>Health service utilisation	Prison cohort n = 30; 100% male
Kaye et al. (2020) [254]	Measure the prevalence, correlates, and comorbidity relating to crystalline methamphetamine use prior to incarceration among young people entering detention	NSW	Youth detention	Mental health status<br>Cognitive disabilities<br>Substance dependence and substance-related harm	N = 227; 91% male; 9% female
Keen et al. (2020) [255]	Determine agreement between self-reported and medically verified histories of non-fatal overdose among people recently released from prison	QLD	Prison	Mental health status<br>Substance dependence and substance-related harm<br>Physical health status<br>Health service utilisation	N = 1307; n = 1030 male; n = 277 female
Keen et al. (2020) [256]	Measure the incidence, predictors, and characteristics associated with non-fatal overdose among people released from prison	QLD	Post-release<br>Prison	Mental health status<br>Substance dependence and substance-related harm<br>Physical health status<br>Health service utilisation	N = 1307; n = 1030 male; n = 277 female
Kelly et al. (2018) [257]	Evaluate the utility of non-invasive assessment of cirrhosis in a clinical setting	NSW	Prison<br>Other	Bloodborne viruses and other communicable diseases<br>Non-communicable diseases	Prison cohort: n = 572 (sex/gender not reported)
Kendall et al. (2019) [258]	Capture the perceptions and experiences of Aboriginal women experiencing incarceration on health, healthcare, and social and emotional well-being	NSW	Prison	Mental health status<br>Substance dependence and substance-related harm<br>Non-communicable diseases<br>Sexual and reproductive health<br>Violence victimisation and injury<br>Health service utilisation	N = 43; 100% female
Kendall et al. (2020) [259]	Capture the lived experiences of healthcare access among Aboriginal women incarcerated in prison	NSW	Prison	Mental health status<br>Cognitive disabilities<br>Substance dependence and substance-related harm<br>Physical health status<br>Sexual and reproductive health<br>Violence victimisation and injury<br>Health service utilisation	N = 43; 100% female

Author (year)	Study aim(s)	Location	Setting	Mapped health domains	Sample
Kenny and Grant (2007) [260]	Determine the reliability of young people's self-reported physical and mental health and risk behaviours	NSW	Youth detention	Mental health status<br>Physical health status (including exercise and obesity)<br>Violence victimisation and injury	*N* = 242; *n* = 223 male; *n* = 19 female
Kenny and Lennings (2007) [261]	Determine cultural group differences in sociodemographic characteristics, offending behaviour, experiences of trauma, and psychopathology among young people in custody	NSW	Youth detention	Mental health status<br>Substance dependence and substance-related harm<br>Violence victimisation and injury	*N* = 251; *n* = 232 male; *n* = 19 female
Kenny et al. (2008) [262]	Determine risk factors associated with suicidal and self-harm behaviour among youth in custody	NSW	Youth detention	Mental health status<br>Cognitive disabilities<br>Substance dependence and substance-related harm<br>Violence victimisation and injury	*N* = 242; *n* = 223 male; *n* = 19 female
Kerley and Cunneen (1995) [263]	Review and describe the deaths of Aboriginal and Torres Strait Islander women in custody between 1980 and 1989	National	Prison<br>Other	Physical health status	*N* = 11; 100% female
Kerry et al. (2024) [264]	Compare the neuropsychological profiles of adolescents in youth detention	WA	Youth detention	Cognitive Disabilities	*N* = 85; *n* = 78 male; *n* = 6 female; *n* = 1 transgender
Kerslake et al. (2020) [265]	Determine the prevalence and correlates associated with risky alcohol use among people entering prisons	QLD<br>SA<br>NT<br>TAS<br>VIC<br>ACT	Prison	Substance dependence and substance-related harm<br>Bloodborne viruses and other communicable diseases	*N* = 387; *n* = 377 male; *n* = 49 female
Kim et al. (2023) [266]	Identify the prevalence of substance use and characteristics associated among women who enter prison pregnant	NSW	Prison	Substance dependence and substance-related harm<br>Sexual and reproductive health	*N* = 141; 100% female
Kinner (2006) [267]	Explore patterns of health and substance use prior to exiting prison and post-release; identify risk factors for poor health and substance misuse	QLD	Post-release<br>Prison	Mental health status<br>Substance dependence and substance-related harm<br>Physical health status	*N* = 160 (sex/gender not reported)
Kinner (2006) [268]	Examine the patterns of alcohol and drug use, mental health, and socioeconomic status of people released from prison; identify predictors of recidivism	QLD	Post-release<br>Prison	Mental health status<br>Substance dependence and substance-related harm<br>Physical health status	*N* = 160; *n* = 108 male; *n* = 52 female
Kinner and Forsyth (2016) [269]	Assess the feasibility of a national system that monitors the mortality in people released from prisons	QLD	Post-release<br>Prison	Physical health status	*N* = 648; *n* = 605 male; *n* = 43 female
Kinner et al. (2016) [270]	Assess the impact of case management on health service utilisation within 6-months after release from prison	QLD	Prison	Mental health status<br>Substance dependence and substance-related harm<br>Bloodborne viruses and other communicable diseases<br>Health service utilisation	*N* = 1325; *n* = 78.5% male, intervention group; *n* = 79.2% male, control group
Kinner et al. (2014) [271]	Determine the prevalence of risky health behaviours and indicators among young people in custody and serving community orders	VIC	Youth detention<br>Other	Mental health status<br>Substance dependence and substance-related harm<br>Physical health status<br>Violence victimisation and injury	Youth in detention: *n* = 273; 86% male

Author (year)	Study aim(s)	Location	Setting	Mapped health domains	Sample
Kinner et al. (2012) [272]	Determine the prevalence and correlates of alcohol dependence among Indigenous and non-Indigenous people experiencing incarceration in prisons	QLD	Prison	Mental health status<br>Cognitive disabilities<br>Substance dependence and substance-related harm	*N* = 1155; 82.3% non-Indigenous male; 71.9% Indigenous male
Kinner et al. (2012) [273]	Estimate the prevalence of recent injecting drug use among adults incarcerated in prisons and identify correlates of injecting behaviour	QLD	Prison	Mental health status<br>Substance dependence and substance-related harm<br>Bloodborne viruses and other infectious diseases<br>Health service utilisation	*N* = 1322 (sex/gender not reported)
Kinner et al. (2013) [274]	Compare the effect of a service brokerage intervention on health service utilisation, health, and offending among people released from prison	QLD	Post-release<br>Prison	Mental health status<br>Substance dependence and substance-related harm<br>Bloodborne viruses and other communicable diseases<br>Physical health status<br>Health service utilisation	*N* = 1325; 97% male
Kinner et al. (2011) [275]	Estimate mortality rates for people released from prison in the 2007-2008 financial year	WA<br>NSW	Post-release<br>Prison	Substance dependence and substance-related harm<br>Physical health status	*N* = 98812; 88.1% male
Kippin et al. (2021) [276]	Explore the macro- and micro-structure factors associated with oral and written language skills among young people in detention	WA	Youth detention	Cognitive disabilities<br>Physical health status	*N* = 30; *n* = 22 male; *n* = female
Kippin et al. (2018) [277]	Examine the language diversity and impairment among young people in custody	WA	Youth detention	Cognitive disabilities<br>Physical health status<br>Health service utilisation	*N* = 98; *n* = 91 male; *n* = 6 female; *n* = 1 transgender
Kirwan et al. (2019) [278]	Describe the prison and transitional health cohort study methodology and report the baseline characteristics of people prior to release from prison	VIC	Prison	Mental health status<br>Substance dependence and substance-related harm<br>Bloodborne viruses and other communicable diseases<br>Non-communicable diseases<br>Physical health status<br>Health service utilisation	*N* = 400; 100% male
Kirwan et al. (2015) [279]	Examine correlates of self-reported property crime among people recently released from prison who have a history of injecting drugs	VIC	Post-release<br>Prison	Mental health status<br>Substance dependence and substance-related harm<br>Health service utilisation	*N* = 114; *n* = 81 male; *n* = 27 females
Koller and Castanos (1969) [280]	Comparison between people who report a history of suicidal behaviour and a matched cohort with no history	NSW	Prison	Mental health status	*N* = 592; *n* = 520 male; *n* = 72 female
Korobanova et al. (2022) [281]	Compare prevalence rates between self-reported psychiatric history and screening among people incarcerated in prison	NSW	Prison	Mental health status<br>Cognitive disabilities<br>Substance dependence and substance-related harm<br>Health service utilisation	*N* = 1132; *n* = 375 female
Kosky et al. (1996) [282]	Examine the social conditions and prevalence of mental health problems for young people after release from youth detention	SA	Post-release<br>Youth detention	Mental health status<br>Substance dependence and substance-related harm	*N* = 101; *n* = 75 male; *n* = 26 female
Kosky et al. (1990) [283]	Evaluate the prevalence of emotional and behavioural disorders among young people in custody	SA	Youth detention	Mental health status<br>Substance dependence and substance-related harm	*N* = 88; *n* = 55 male; *n* = 23 female
Lafferty et al. (2020) [284]	Capture the experiences and perceptions of hepatitis C treatment among people incarcerated in prisons	NSW	Prison	Substance dependence and substance-related harm<br>Bloodborne viruses and other communicable diseases<br>Health service utilisation	*N* = 23; 100% male

Author (year)	Study aim(s)	Location	Setting	Mapped health domains	Sample
Lafferty et al. (2023) [285]	Evaluate a prison-based hepatitis C testing program from the perspectives of men incarcerated in prison	NSW	Prison	Substance dependence and substance-related harm<br>Bloodborne viruses and other communicable diseases<br>Health service utilisation	*N* = 24; 100% male
Lafferty et al. (2023) [286]	Explore the level of trust and engagement in service providers among people who inject drugs after release from prison	VIC	Post-release<br>Prison	Mental health status<br>Substance dependence and substance-related harm<br>Health service utilisation	*N* = 48; *n* = 33 male; *n* = 15 female
Larney et al. (2014) [287]	Explore mortality in prison among people who are opioid dependent and explore the impact of opioid substitution therapy on mortality risk in prison	NSW	Prison	Mental health status<br>Substance dependence and substance-related harm<br>Physical health status<br>Violence victimisation and injury<br>Health service utilisation	*N* = 16715; *n* = 13199 male
Larney et al. (2023) [288]	Examine mortality risk among older adults who have received opioid agonist treatment	NSW	Community<br>Prison history	Substance dependence and substance-related harm<br>Health service utilisation	*N* = 37764; *n* = 26018 male; *n* = 11744 female<br>Prison history: *n* = 17041
Larney et al. (2016) [289]	Describe the findings and impact of a prison-based opioid substitution therapy program and retention rates among people after release from prison	NSW	Prison	Substance dependence and substance-related harm<br>Health service utilisation	*N* = 8577; *n* = 6,948 male
Larney et al. (2012) [290]	Determine the prevalence of suicide ideation and attempts among people incarcerated in prisons	NSW	Prison	Mental health status	*N* = 996; *n* = 797 male; *n* = 199 females
Larney et al. (2011) [291]	Evaluate the effect of prison-based opioid substitution treatment during imprisonment and post-release, and determine the effect of treatment utilisation on reincarceration	NSW	Prison	Substance dependence and substance-related harm<br>Health service utilisation	*N* = 374; 100% male
Lawlor and Kosky (1992) [292]	Determine the incidence and nature of serious suicide attempts among young people on remand in Perth	WA	Youth detention	Mental health status<br>Substance dependence and substance-related harm<br>Health service utilisation	*N* = 42; 84% male; 16% female
Le Gautier et al. (2023) [293]	Identify patterns of end-of-life and palliative care among people incarcerated in prisons	VIC	Prison	Physical health status<br>Health service utilisation	*N* = 15; 100% male
Leckning et al. (2023) [294]	Identify patterns of youth justice involvement and child protection history and associations with the prevalence and incidence of hospitalisations due to mental illness; compare between Aboriginal and non-Aboriginal adolescents	NT	Youth detention<br>Other	Mental health status<br>Health service utilisation	*N* = 14972; 52% male; 48% female
Lennings and Pritchard (1999) [295]	Determine the prevalence of substance use prior to entering youth detention	QLD	Youth detention	Substance dependence and substance-related harm	*N* = 118; 90% male
Levy et al. (2007) [296]	Examine changes in the prevalence of Mantoux positivity between 1996 and 2001 prison cohorts and determine the annual risk of tuberculosis infection	NSW	Prison	Bloodborne viruses and other communicable diseases	*N* = 1442; *n* = 1190 male; *n* = 252 female
Levy et al. (2003) [297]	Describe a varicella outbreak that occurred within NSW prisons	NSW	Prison	Bloodborne viruses and other communicable diseases	*N* = 1800; *n* = 1550 male; *n* = 250 female

Author (year)	Study aim(s)	Location	Setting	Mapped health domains	Sample
Levy et al. (2003) [298]	Describe an investigation into an outbreak of tonsillopharyngitis in a rural NSW prison	NSW	Prison	Bloodborne viruses and other communicable diseases	*N* = 87; 100% male
Lewis and Hayes (1997) [299]	Describe the health of females after release from prison, compared with a community sample	NSW	Post-release<br>Prison	Mental health status<br>Substance dependence and substance-related harm<br>Physical health status	Prison history: *n* = 30; 100% female
Li et al. (2020) [300]	Examine the incidence and predictors of hepatitis B transmission and vaccination uptake among people incarcerated in prison	NSW	Prison	Substance dependence and substance-related harm<br>Bloodborne viruses and other communicable diseases<br>Violence victimisation and injury<br>Health service utilisation	*N* = 140; *n* = 102 male
Liang et al. (2023) [301]	Examine evidence-based alcohol relapse prevention treatments provided within NSW Prisons	NSW	Prison	Mental health status<br>Cognitive disabilities<br>Substance dependence and substance-related harm<br>Bloodborne viruses and other communicable diseases<br>Non-communicable diseases<br>Health service utilisation	*N* = 50; *n* = 38 male; *n* = 12 female
Liao et al. (2023) [302]	Determine the factors associated with acute care and emergency department presentation for epilepsy among people with intellectual disability	NSW	Prison<br>Other	Cognitive disabilities<br>Non-communicable diseases<br>Health service utilisation	*N* = 3293; *n* = 1880 male
Lloyd et al. (2013) [303]	Evaluate the safety and utility of nurse-led care for people with chronic hepatitis C in prisons	NSW	Prison	Mental health status<br>Substance dependence and substance-related harm<br>Bloodborne viruses and other communicable diseases<br>Physical health status<br>Health service utilisation	*N* = 391; *n* = 385 male; *n* = 6 female
Lloyd et al. (2017) [304]	Identify risk of hospitalisation for Aboriginal and non-Aboriginal people after release from custody	NSW	Prison	Mental health status<br>Bloodborne viruses and other communicable diseases<br>Substance dependence and substance-related harm<br>Health service utilisation	*N* = 1075 (sex/gender not reported)
Love et al. (2017) [305]	Investigate the relationship between area disadvantage and rates of hospitalisation after release from prison	QLD	Post-release<br>Prison	Mental health status<br>Substance dependence and substance-related harm<br>Non-communicable diseases<br>Physical health status<br>Health service utilisation	*N* = 1267; *n* = 992 male; *n* = 275 female
Luciani et al. (2014) [306]	Determine the incidence of hepatitis C infection among a prospective cohort of people incarcerated in prisons who inject drugs	NSW	Prison	Substance dependence and substance-related harm<br>Bloodborne viruses and other communicable diseases<br>Violence victimisation and injury	*N* = 210; *n* = 155 male; *n* = 55 female
Lynch et al. (2007) [307]	Explore the physical health, mental health, substance use, intellectual functioning, and social circumstances among youth in custody and and community orders	NSW	Youth detention	Mental health status<br>Cognitive disabilities<br>Substance dependence and substance-related harm<br>Bloodborne viruses and other communicable diseases<br>Non-communicable diseases<br>Physical health status<br>Sexual and reproductive health	*N* = 1044 participants<br>Youth detention cohort: *n* = 242 (sex/gender not reported)

Author (year)	Study aim(s)	Location	Setting	Mapped health domains	Sample
Macdonald et al. (2016) [308]	Capture the lived experience of Indigenous men who have experienced incarceration in prison	NSW	Post-release<br>Prison	Mental health status<br>Substance dependence and substance-related harm<br>Bloodborne viruses and other communicable diseases<br>Non-communicable diseases<br>Physical health status<br>Violence victimisation and injury	*N* = 8; 100% male
Macdonald et al. (2024) [309]	Identify the factors that influence mental health diversion in local courts	NSW	Prison<br>Other	Mental health status<br>Substance dependence and substance-related harm	*N* = 7546; *n* = 5509 male; *n* = 2037 female
MacIntyre et al. (1999) [310]	Identify the prevalence, incidence, and risk factors associated with tuberculosis amongst a prison outbreak	VIC	Prison	Bloodborne viruses and other communicable diseases<br>Health service utilisation	*N* = 136 (sex/gender not reported)
MacLachlan et al. (2020) [311]	Report the prevalence of hepatitis B and C among people incarcerated in VIC prisons	VIC	Prison	Bloodborne viruses and other communicable diseases	*N* = 8149 (sex/gender not reported)
Malacova et al. (2011) [312]	Compare the knowledge of STIs between prison and general population	NSW<br>QLD	Prison	Sexual and reproductive Health	Prison cohort: *n* = 2289; *n* = 1960 male; *n* = 329 female
Malacova et al. (2012) [313]	Explore self-reported experiences of sexual coercion among male and female people in prisons prior to sentencing	NSW<br>QLD	Prison	Violence victimisation and injury	*N* = 2335; *n* = 2006 male; *n* = 329 female
Malvaso et al. (2022) [314]	Examine the rate of adverse childhood experiences among young people in contact with the justice system; explore associations with trauma, substance use, and social and emotional behaviours	SA	Youth detention<br>Other	Mental health status<br>Substance dependence and substance-related harm<br>Violence victimisation and injury	*N* = 184<br>Young people in detention: *n* = 80; 84% male; 16% female
Malvaso et al. (2017) [315]	Investigate the health needs among young men in custody and the relationship between maltreatment, traumatic experiences, and mental health	SA	Youth detention	Mental health status<br>Violence victimisation and injury	*N* = 28; 100% male
Marr et al. (2023) [316]	Compare differences in prevalence of mental illness, substance use, childhood trauma, and suicidal and self-harm behaviour among youth in detention between years 2003, 2009, and 2015	NSW	Youth detention	Mental health status<br>Substance dependence and substance-related harm<br>Violence victimisation and injury	*N* = 800; *n* = 720 male; *n* = 79 female
Marshall et al. (2023) [317]	Capture the perspectives and experiences of injecting drug use and opioid agonist therapy from people recently released from prisons	VIC	Post-release<br>Prison	Substance dependence and substance-related harm<br>Health service utilisation	*N* = 48; *n* = 33 male
McDonald et al. (1999) [318]	Measure the extent and impact of HIV testing among people entering prison between 1991 and 1997	National	Prison	Bloodborne viruses and other communicable diseases	*N* = 222925 (sex/gender not reported)
McDonald and Thomson (1993) [319]	Assess the cause of death for people in prison or police custody in Australia and compare differences between Aboriginal and non-Aboriginal deaths in custody	National	Prison<br>Other	Mental health status<br>Substance dependence and substance-related harm<br>Non-communicable diseases<br>Physical health status<br>Violence victimisation and injury	*N* = 456; *n* = 429 male; *n* = 27 female

Author (year)	Study aim(s)	Location	Setting	Mapped health domains	Sample
McGillivray et al. (2016) [320]	Examine substance use prior to incarceration among people with and without intellectual disability and explore the association between substance use and offending	VIC	Prison	Cognitive disabilities<br>Substance dependence and substance-related harm<br>Health service utilisation	Total sample size not reported; *n* = 183 male; *n* = 17 female
McGregor et al. (2002) [321]	Identify the circumstances and characteristics associated with accidental heroin-related deaths in SA between 1994 and 1997	SA	Prison<br>Other	Substance dependence and substance-related harm	*N* = 101; *n* = 71 male; *n* = 30 female
Merone et al. (2022) [322]	Explore events that influenced an increase in the prevalence of hepatitis C infection in a QLD prison	QLD	Prison	Substance dependence and substance-related harm<br>Bloodborne viruses and other communicable diseases	*N* = 250; 100% male
Miller et al. (2006) [323]	Estimate the prevalence of hepatitis C among people incarcerated in SA prisons	SA	Prison	Bloodborne viruses and other communicable diseases	*N* = 1347; *n* = 1254 male; *n* = 93 female
Miller et al. (2009) [324]	Measure the seroprevalence and seroconversion to hepatitis C virus among people entering prisons and identify risk factors associated with exposure	SA	Prison	Substance dependence and substance-related harm<br>Bloodborne viruses and other communicable diseases	*N* = 662; *n* = 596 male; *n* = 66 female
Miller et al. (2009) [325]	Determine risk factors associated with hepatitis C, hepatitis B, and HIV infection among people who inject drugs	VIC	Community<br>Prison history<br>Youth detention history	Substance dependence and substance-related harm<br>Bloodborne viruses and other communicable diseases	*N* = 380; *n* = 252 male; *n* = 128 female
Mills et al. (2008) [326]	Explore experiences of trauma and the development of post-traumatic stress disorder among a prospective cohort of people who use heroin	NSW	Community<br>Prison history	Mental health status<br>Substance dependence and substance-related harm	*N* = 309; 62% male
Miner and Gorta (1987) [327]	Understand the number, characteristics, and needs of females experiencing incarceration who use heroin in NSW	NSW	Prison	Substance dependence and substance-related harm<br>Health service utilisation	*N* = 90; 100% female
Moore et al. (2013) [328]	Determine the prevalence of childhood maltreatment and post-traumatic stress disorder among youth in custody; assess the relationship between childhood maltreatment and post-traumatic stress disorder, and the psychosocial predictors associated	NSW	Youth detention	Mental health status<br>Cognitive disabilities<br>Substance dependence and substance-related harm<br>Violence victimisation and injury	*N* = 291; *n* = 253 male; *n* = 38 female
Moore et al. (2015) [329]	Estimate the prevalence of suicide and self-harm behaviour among youth in detention	NSW	Youth detention	Mental health status<br>Cognitive disabilities<br>Substance dependence and substance-related harm	*N* = 313; *n* = 274 male; *n* = 39 female
Moore et al. (2014) [330]	Determine the prevalence of traumatic brain injury and the association with substance misuse and offending among youth in custody	NSW	Youth detention	Mental health status<br>Cognitive disabilities<br>Substance dependence and substance-related harm<br>Violence victimisation and injury	*N* = 316; *n* = 277 male; *n* = 39 female
Moore et al. (2016) [331]	Determine the prevalence of adult attention-deficit/hyperactivity disorder among people incarcerated in prisons and explore the characteristics associated with substance use and co-morbidity	NSW	Prison	Mental health status<br>Cognitive disabilities<br>Substance dependence and substance-related harm	*N* = 88; 76% male; 24% female

Author (year)	Study aim(s)	Location	Setting	Mapped health domains	Sample
Moore et al. (2013) [332]	Measure the prevalence and correlates of lifetime history of non-fatal overdose among people incarcerated in prisons	NSW<br>QLD	Prison	Mental health status<br>Substance dependence and substance-related harm<br>Bloodborne viruses and other communicable diseases<br>Health service utilisation	*N* = 2288 (sex/gender not reported)
Morrison (1996) [333]	Analysis of suicides in custody and compare between populations incarcerated in different settings	National	Prison	Mental health status<br>Physical health status<br>Violence victimisation and injury	*N* = 480 (sex/gender not reported
Moyo et al. (2018) [334]	Explore tuberculosis notifications in prisons to assess whether targeted interventions are needed	VIC	Prison	Bloodborne viruses and other communicable diseases<br>Health service utilisation	*N* = 26; *n* = 24 male; *n* = 2 female
Murray et al. (2004) [335]	Determine the hearing health of people in NSW prisons	NSW	Prison	Substance dependence and substance-related harm<br>Bloodborne viruses and other communicable diseases<br>Non-communicable diseases	*N* = 497; *n* = 413 male; *n* = 84 female
Newnham et al. (2024) [336]	Review and describe characteristics and risk factors among people in prison treated for cancer	VIC	Prison	Mental health status<br>Substance dependence and substance-related harm<br>Bloodborne viruses and other communicable diseases<br>Non-communicable diseases	*N* = 200; *n* = 185 male; *n* = 15 female
Nielssen et al. (2018) [337]	Identify the characteristics of people in contact with mental health services provided to the homeless	NSW	Community<br>Prison history	Mental health status<br>Cognitive disabilities<br>Substance dependence and substance-related harm	Prison history: *n* = 675; 95% male
Nielssen and Misrachi (2005) [338]	Determine prevalence estimates of psychotic illnesses among men entering prisons in NSW	NSW	Prison	Mental health status	*N* = 728; 100% male
Nielssen et al. (2019) [339]	Examine reincarceration among people released from custody with psychotic and cognitive disorders	NSW	Prison	Mental health status<br>Cognitive disabilities	*N* = 661 (sex/gender not reported)
O'Driscoll et al. (2012) [340]	Investigate how co-occurring substance dependence, personality disorder, and mental illness impacts re-offending rates	NSW	Prison	Mental health status<br>Substance dependence and substance-related harm	*N* = 1264; *n* = 1029 male; *n* = 235 female
O'Driscoll et al. (2007) [341]	Review the extent and nature of suicides in NSW prisons between 1995 and 2005	NSW	Prison	Mental health status<br>Substance dependence and substance-related harm	*N* = 224 (sex/gender not reported)
O'Sullivan et al. (2003) [342]	Investigate the transmission of HIV, hepatitis B, and hepatitis C after exposure through needle and syringe sharing in prisons	NSW	Prison	Bloodborne viruses and other communicable diseases	*N* = 104 (sex/gender not reported)
Ober et al. (2013) [343]	Assess the utility of the Indigenous Risk Impact Screen tool among Indigenous people incarcerated in prisons	QLD	Prison	Mental health status<br>Substance dependence and substance-related harm	*N* = 395; 83.4% male
Ogilvie et al. (1999) [344]	Determine patterns and risks associated with hepatitis A, B, and C infection among young males in detention	VIC	Youth detention	Substance dependence and substance-related harm<br>Bloodborne viruses and other communicable diseases<br>Physical health status	*N* = 90; 100% male
Ogloff et al. (2017) [345]	Determine the social and emotional wellbeing and mental illness among Aboriginal people experiencing incarceration in prison	VIC	Prison	Mental health status<br>Cognitive disabilities<br>Substance dependence and substance-related harm<br>Health service utilisation	*N* = 122; *n* = 107 male; *n* = 15 female
Osborn et al. (2003) [346]	Examine oral health status and risk factors associated with oral health among people incarcerated in prisons	NSW	Prison	Physical health status	*N* = 789; *n* = 657 male; *n* = 132 female

Author (year)	Study aim(s)	Location	Setting	Mapped health domains	Sample
Overton et al. (2019) [347]	Determine the efficacy of a nurse-led program for the treatment and assessment of hepatitis C among people entering prisons	NSW	Prison	Mental health status<br>Bloodborne viruses and other communicable diseases<br>Non-communicable diseases<br>Physical health status<br>Health service utilisation	*N* = 698; *n* = 633 male; *n* = 65 female
Panozzo et al. (2021) [348]	Examine the end-of-life care provided to people incarcerated in prison and compare to with a non-incarcerated matched cohort	VIC	Prison	Mental health status<br>Physical health status<br>Health service utilisation	*N* = 155; Prison cohort: *n* = 55; *n* = 52 male; *n* = female
Papalia et al. (2022) [349]	Identify the rates of physical, sexual, emotional abuse, and neglect experienced by young people in custody	VIC	Youth detention	Mental health status<br>Substance dependence and substance-related harm<br>Violence victimisation and injury<br>Health service utilisation	*N* = 215; 82% male
Papalia et al. (2024) [350]	Assess the discriminative and predictive validity of risk assessment measures among a sample of women incarcerated for violent offences	VIC	Prison	Substance dependence and substance-related harm	*N* = 79; 100% female
Papaluca et al. (2022) [351]	Evaluate the effect of transitional programs on hepatitis C treatment engagement after release from prison	VIC	Prison	Mental health status<br>Substance dependence and substance-related harm<br>Bloodborne viruses and other communicable diseases<br>Health service utilisation	*N* = 46; *n* = 27 male
Papaluca et al. (2019) [352]	Measure the effectiveness of a nurse-led treatment program for people incarcerated in prisons with hepatitis C	VIC	Prison	Mental health status<br>Substance dependence and substance-related harm<br>Bloodborne viruses and other communicable diseases<br>Non-communicable diseases<br>Physical health status<br>Health service utilisation	*N* = 416; *n* = 376 male
Perkes et al. (2011) [353]	Compare the prevalence of past-reported traumatic brain injury between people incarcerated in prisons and a control group	NSW	Prison	Mental health status<br>Cognitive disabilities<br>Substance dependence and substance-related harm<br>Violence victimisation and injury	*N* = 400; 100% male
Petschel and Gall (2000) [354]	Review all records of deaths in custody in Victoria between 1991 and 1996	VIC	Prison<br>Other	Mental health status<br>Substance dependence and substance-related harm<br>Physical health status<br>Violence victimisation and injury	*N* = 96 (sex/gender not reported)
Pham et al. (2010) [355]	Examine the presence of multiple hepatitis C infection episodes among seronegative people incarcerated in prisons	NSW	Prison	Bloodborne viruses and other communicable diseases	*N* = 488; 65% male
Pounder (1986) [356]	Review the cause, location, and circumstances related to deaths in custody between 1972 and 1983	SA	Prison<br>Other	Mental health status<br>Substance dependence and substance-related harm<br>Bloodborne viruses and other communicable diseases<br>Non-communicable diseases<br>Physical health status (including exercise and obesity)	*N* = 31; 100% male
Puljević et al. (2019) [357]	Explore factors associated with maintained smoking abstinence after release from smoke-free prisons	QLD	Post-release<br>Prison	Substance dependence and substance-related harm	*N* = 21; *n* = 16 male; *n* = 5 female

Author (year)	Study aim(s)	Location	Setting	Mapped health domains	Sample
Puljević et al. (2018)[358]	Identify the uptake of smoking cessation pharmacotherapy among people released from prison	QLD	Post-release<br>Prison	Mental health status<br>Substance dependence and substance-related harm<br>Non-communicable diseases<br>Physical health status<br>Health service utilisation	*N* = 971; *n* = 221 female
Puljević et al. (2018)[359]	Explore relapse to smoking and correlates associated with reduced consumption among people recently released from smoke-free prisons	QLD	Post-release<br>Prison	Mental health status<br>Substance dependence and substance-related harm<br>Physical health status	*N* = 114; *n* = 98 male
Putniņš (1995)[360]	Examine patterns of psychoactive substance use among young people in detention	SA	Youth detention	Mental health status<br>Substance dependence and substance-related harm	*N* = 216; 85.1% male
Putniņš (1992)[361]	Evaluate the usefulness of the Adolescent Alcohol Involvement Scale as a clinical screening tool in a sample of young people in custody	SA	Youth detention	Substance dependence and substance-related harm	*N* = 197; *n* = 172 male; *n* = 25 female
Putniņš (2005)[362]	Examine correlates and predictors of suicidal behaviours among young people in custody	SA	Youth detention	Mental health status	*N* = 900; 90% male; 10% female
Putniņš and Harvey (1992)[363]	Investigate patterns of alcohol use among young people in custody and assess the behaviours, circumstances, and consequences associated with alcohol abuse	SA	Youth detention	Substance dependence and substance-related harm	*N* = 197; 87.3% male
Quinn and Rance (2009)[364]	Measure the extent of hearing impairment among Indigenous people experiencing incarceration in prisons	VIC	Prison	Physical health status	*N* = 109; *n* = 96 male; *n* = 13 female
Rasmussen et al. (2018)[365]	Examine the impact of an Aboriginal art program on suicidal and self-harm behaviours among Aboriginal people incarcerated in prison	QLD	Prison	Mental health status<br>Substance dependence and substance-related harm	*N* = 335; 100% male
Reekie et al. (2014)[366]	Measure the prevalence of markers for hepatitis B, C, and HIV infection among people entering prisons	NSW<br>QLD<br>WA<br>TAS	Prison	Substance dependence and substance-related harm<br>Bloodborne viruses and other communicable diseases	*N* = 1742; *n* = 1567 male
Reser (1989)[367]	Assess suicides in custody from 1980 to 1988 and compare the difference between Aboriginal and non-Aboriginal people's suicides in custody	National	Prison<br>Other	Mental health status	*N* = 462 (sex/gender not reported)
Reutens et al. (2023)[368]	Examine and compare the legal outcomes between older and younger people who have committed offences	National	Prison<br>Other	Mental health status<br>Cognitive disabilities<br>Substance dependence and substance-related harm	*N* = 41; *n* = 40 male; *n* = 1 sex/gender not reported
Rice et al. (2021)[369]	Determine the prevalence of bladder and bowel dysfunction among women experiencing incarceration in prison and explore effects on quality of life	WA	Prison	Substance dependence and substance-related harm<br>Physical health status	*N* = 29; 100% female
Riches et al. (2006)[370]	Report the prevalence and needs of people with intellectual disabilities who experience incarceration in NSW	NSW	Prison	Cognitive disabilities	Sample size and characteristics not reported

Author (year)	Study aim(s)	Location	Setting	Mapped health domains	Sample
Richmond et al. (2013) [371]	Explore characteristics of smoking, drug and alcohol use, sociodemographic circumstances, and general health of people incarcerated in prisons; compare differences between Aboriginal and non-Aboriginal people incarcerated in prisons	NSW	Prison	Mental health status<br>Substance dependence and substance-related harm<br>Physical health status	$N = 425$; 100% male
Richmond et al. (2013) [372]	Determine the efficacy of a smoking cessation intervention among people incarcerated in prison	NSW<br>QLD	Prison	Substance dependence and substance-related harm	$N = 425$; 100% male
Richmond et al. (2011) [373]	Compare the cardiovascular risk factors associated with smoking between males incarcerated in prison and a matched community sample	NSW<br>QLD	Prison	Mental health status<br>Substance dependence and substance-related harm<br>Physical health status	$N = 425$; 100% male
Richters et al. (2012) [374]	Explore experiences of sexual coercion and consensual sex among men incarcerated in prisons	QLD<br>NSW	Prison	Violence victimisation and injury	$N = 2018$; 100% male
Riddell et al. (2006) [375]	Identify associations between amphetamine use, psychiatric disorder, and offending behaviour among people on remand in NSW prisons	NSW	Prison	Mental health status<br>Substance dependence and substance-related harm<br>Health service utilisation	$N = 888$ (sex/gender not reported)
Riley et al. (2019) [376]	Examine the effectiveness of a group therapy program designed to increase mindfulness and acceptance among women experiencing incarceration with mental health problems	SA	Prison	Mental health status<br>Substance dependence and substance-related harm<br>Health service utilisation	$N = 59$; 100% male
Rogerson et al. (2016) [377]	Examine cannabis use, dependence, and withdrawal among Indigenous males incarcerated in prisons	QLD	Prison	Substance dependence and substance-related harm	$N = 101$; 100% male
Rose et al. (2020) [378]	Assess the psychological distress and symptoms of mental illness among people incarcerated in prisons and compare differences between cultural groups	VIC	Prison	Mental health status	$N = 194$; 100% male
Rose et al. (2019) [379]	Determine the prevalence of well-being, distress, and coping among Indigenous and culturally diverse people incarcerated in prisons	VIC	Prison	Mental health status<br>Health service utilisation	$N = 477$; 100% male
Ross et al. (2019) [380]	Identify the prevalence and predictors of poor physical health among people incarcerated in prison	NSW	Prison	Mental health status<br>Substance dependence and substance-related harm<br>Bloodborne viruses and other communicable diseases<br>Non-communicable diseases<br>Physical health status<br>Health service utilisation	$N = 1098$; $n = 746$ male; $n = 352$ female
Russell et al. (2023) [381]	Report the characteristics, assessment processes, and recommendations related to justice-involved young people that have been referred to a clinical service for fetal alcohol spectrum disorder assessment	WA<br>NT	Youth detention<br>Other	Mental health status<br>Cognitive disabilities	$N = 64$; $n = 52$ male

Author (year)	Study aim(s)	Location	Setting	Mapped health domains	Sample
Ryan et al. (2019) [382]	Explore the impact of social experiences before and during prison, and differences between Indigenous and non-Indigenous people's risk of reincarceration	QLD	Post-release<br>Prison	Mental health status<br>Substance dependence and substance-related harm	*N* = 1238; 79% male; 21% female
Ryan et al. (2020) [383]	Explore the impact of prisoner visitation on risk of reincarceration between Indigenous and non-Indigenous people	QLD	Post-release<br>Prison	Substance dependence and substance-related harm	*N* = 1238; 79% male; 21% female
Sapkota et al. (2022) [384]	Capture the experiences and in-prison and post-release needs of pregnant women and new mothers who are incarcerated in prison	QLD	Prison	Sexual and reproductive health	*N* = 75; 100% female
Sawyer et al. (2010) [385]	Compare the characteristics and prevalence of mental health problems, suicidal ideation and behaviours, and quality of life between young people on remand and young people in the general community	SA	Youth detention	Mental health status	*N* = 159; 83% male; 17% female
Sawyer et al. (2010) [386]	Explore changes in the prevalence of mental health problems among young people on remand between years 1989, 2008, and 2009	SA	Youth detention	Mental health status	2008/09 cohort: *n* = 197; 82% male; 18% female
Sazzad et al. (2020) [387]	Explore the risk of hepatitis C transmission due to violence within the prison setting	NSW	Prison	Substance dependence and substance-related harm<br>Bloodborne viruses and other communicable diseases<br>Violence victimisation and injury	*N* = 23; *n* = 15 male; *n* = 8 female
Schilders and Ogloff (2014) [388]	Examine outcomes of reception screening for people entering prisons during 2009	VIC	Prison	Mental health status<br>Health service utilisation	*N* = 4229; 100% male
Schneider et al. (2011) [389]	Examine the relationship between history of experiencing sexual coercion, physical or sexual assault, and psychological distress among people incarcerated in prisons	QLD<br>NSW	Prison	Mental health status<br>Physical health status<br>Violence victimisation and injury	*N* = 2351; *n* = 2018 male; *n* = 333 female
Schofield et al. (2006a) [390]	Explore the relationship between traumatic brain injury and demographic, neuropsychiatric, and criminological characteristics among people entering custody	NSW	Prison<br>Other	Mental health status<br>Substance dependence and substance-related harm<br>Cognitive disabilities	*N* = 200; 100% male
Schofield et al. (2006b) [391]	Determine the prevalence, extent, recurrence, and sequalae of traumatic brain injury among people entering prisons	NSW	Prison	Mental health status<br>Cognitive disabilities<br>Substance dependence and substance-related harm<br>Physical health status<br>Violence victimisation and injury<br>Health service utilisation	*N* = 200; 100% male
Schofield et al. (2011) [392]	Assess the reliability of people incarcerated in prison's self-reported histories of traumatic brain injury associated with hospitalisation by linking data with medical records	NSW	Prison	Cognitive disabilities<br>Health service utilisation	*N* = 200 (sex/gender not reported)

Author (year)	Study aim(s)	Location	Setting	Mapped health domains	Sample
Seamark et al. (1997) [393]	Determine the prevalence of HIV in SA prisons between 1989 and 1994	SA	Prison	Substance dependence and substance-related harm<br>Bloodborne viruses and other communicable diseases	Prevalence study: *N* = 39; 100% male<br>Case-control: *N* = 119; 100% male
Selvey et al. (1996) [394]	Investigate notifications of hepatitis C cases in 1994 and establish the risk factors associated with infection	QLD<br>NT<br>ACT	Prison<br>Other	Bloodborne viruses and other communicable diseases	*N* = 1144; 60% male
Sheehan et al. (2023) [395]	Assess the effectiveness of a hepatitis C testing intervention on treatment engagement among people entering prisons	NSW	Prison	Bloodborne viruses and other communicable diseases<br>Health service utilisation	*N* = 540; 100% male
Shepherd et al. (2018) [396]	Identify predictors of psychological distress among Indigenous people experiencing incarceration in prison	VIC	Prison	Mental health status<br>Substance dependence and substance-related harm	*N* = 87; *n* = 75 male; *n* = 12 female
Shepherd et al. (2016) [397]	Determine the health needs of Indigenous adults incarcerated in prisons	VIC	Prison	Mental health status<br>Substance dependence and substance-related harm<br>Physical health status	*N* = 122; *n* = 107 male; *n* = 15 female
Shepherd et al. (2017) [398]	Explore the association between cognitive impairment, mental health, and cultural needs among Aboriginal and Torres Strait Islander people incarcerated in prison	VIC	Prison	Mental health status<br>Cognitive disabilities	*N* = 122; *n* = 107 male; *n* = 15 female
Shepherd et al. (2017) [399]	Determine the prevalence of cognitive disability and dual diagnosis among Aboriginal people experiencing incarceration in prison	VIC	Prison	Mental health status<br>Cognitive disabilities<br>Substance dependence and substance-related harm	*N* = 122; *n* = 107 male; *n* = 15 female
Shepherd et al. (2018) [400]	Measure the prevalence and correlates of suicidal ideation and behaviours among Indigenous males experiencing incarceration in prison	VIC	Prison	Mental health status	*N* = 107; 100% male
Shepherd et al. (2018) [401]	Investigate the prevalence of self-harm and suicidal behaviours among young people in custody	VIC	Youth detention	Mental health status	*N* = 215; *n* = 177 male; *n* = 38 female
Shinkfield and Graffam (2009) [402]	Examine the support provided to people exiting prisons and the impact on community reintegration	VIC	Prison	Mental health status<br>Substance dependence and substance-related harm<br>Physical health status	*N* = 79; *n* = 54 male; *n* = 25 female
Shinkfield and Graffam (2010) [403]	Examine the association between emotional state and successful reintegration for people released from prison	VIC<br>QLD	Post-release<br>Prison	Mental health status	*N* = 101; *n* = 72 male; *n* = 29 female
Silva et al. (2014) [404]	Investigate contact with the juvenile justice system among young people with and without attention-deficit/hyperactivity disorder	WA	Youth detention<br>Other	Cognitive disabilities	Custody history: *n* = 256; *n* = 240 male; *n* = 16 female
Sindicich et al. (2014) [405]	Examine the clinical and criminal profiles of men experiencing incarceration in prisons with symptoms of substance use disorder and post-traumatic stress disorder	NSW	Prison	Mental health status<br>Substance dependence and substance-related harm<br>Violence victimisation and injury<br>Health service utilisation	*N* = 30; 100% male

Author (year)	Study aim(s)	Location	Setting	Mapped health domains	Sample
Skov et al. (1997)[406]	Evaluate the effectiveness of a urine test intervention for diagnosing and testing gonorrhoea and chlamydia for men living in remote Aboriginal communities. Estimate the prevalence of infection within these communities	NT	Prison<br>Other	Sexual and reproductive health	Prison cohort *n* = 33; 100% male
Smirnov et al. (2016)[407]	Identify factors associated with substance dependence among Indigenous and non-Indigenous people	QLD	Community<br>Prison history	Mental health status<br>Substance dependence and substance-related harm	*N* = 566 (sex/gender not reported)
Smirnov et al. (2018)[408]	Examine the relationship between drug dependency and exposure to hepatitis C infection among Indigenous and non-Indigenous people who inject drugs	QLD	Community<br>Prison history	Substance dependence and substance-related harm<br>Bloodborne viruses and other communicable diseases	*N* = 470; 61.2% Indigenous male; 38.8% Indigenous female; 69.2% non-Indigenous male; 30.8% non-Indigenous female)
Snow et al. (2016)[409]	Identify the language, mental health, and emotion recognition among youth in detention	NSW	Youth detention	Mental health status<br>Cognitive disabilities	*N* = 100; *n* = 85 male; *n* = 15 female
Snow et al. (2017)[410]	Compare prevalence estimates for hepatitis C virus among people incarcerated in prisons	QLD	Prison	Substance dependence and substance-related harm<br>Bloodborne viruses and other communicable diseases	*N* = 1315 participants (78.9% male)
Snow et al. (2014)[411]	Examine the prevalence, incidence, and correlates of hepatitis C seropositivity among people incarcerated in prisons with a history of injecting drugs	QLD	Prison	Substance dependence and substance-related harm<br>Bloodborne viruses and other communicable diseases	*N* = 735; 77% male
Snow and Powell (2011)[412]	Determine the extent and nature of oral language impairment among young people in detention	VIC	Youth detention	Cognitive disabilities<br>Physical health status	*N* = 100; 100% male
Snow et al. (2022)[413]	Estimate the cost associated with primary care, emergency, and in-patient health service utilisation among people released from prison; examine the effect of recent mental health, substance use, or dual diagnosis on healthcare costs, criminal justice costs, and reincarceration	QLD	Prison	Mental health status<br>Substance dependence and substance-related harm	*N* = 1303; *n* = 277 female
Sodhi-Berry et al. (2015)[414]	Explore levels of mental health service utilisation prior to incarceration and how this effects the probability of utilisation after release from prison	WA	Prison	Mental health status<br>Substance dependence and substance-related harm<br>Health service utilisation	*N* = 1853; 80% male; 20% female
Sodhi-Berry et al. (2015)[415]	Identify how psychiatric treatment history effects post-release mortality outcomes	WA	Post-release<br>Prison	Mental health status<br>Substance dependence and substance-related harm<br>Non-communicable diseases<br>Physical health status<br>Health service utilisation	*N* = 25537; 88.1% male; 11.9% female
Sodhi-Berry et al. (2015)[416]	Determine predictors of mental health service utilisation up to five years after release from prison	WA	Post-release<br>Prison	Mental health status<br>Health service utilisation	*N* = 23661; *n* = 18426 male

Author (year)	Study aim(s)	Location	Setting	Mapped health domains	Sample
Spittal et al. (2019) [417]	Evaluate modifiable risk factors associated with external cause and cause-specific death of people released from prisons compared to matched controls	QLD	Post-release<br>Prison	Mental health status<br>Substance dependence and substance-related harm<br>Bloodborne viruses and other communicable diseases<br>Violence victimisation and injury<br>Health service utilisation	*N* = 572; *n* = 98 male; *n* = 474 female
Spittal et al. (2014) [418]	Compare the risk of opioid overdose mortality with risk of suicide among people released from prison over a 14-year period	QLD	Post-release<br>Prison	Mental health status<br>Substance dependence and substance-related harm	*N* = 41970; *n* = 36994 male
Spivak et al. (2020) [419]	Measure the lifetime prevalence and correlates of crystalline methamphetamine use prior to incarceration in youth detention; explore the relationship between use and future offending behaviours	VIC	Youth detention	Mental health status<br>Substance dependence and substance-related harm	*N* = 202; *n* = 164 male; *n* = 38 female
Stathis et al. (2012) [420]	Use the Westerman Aboriginal Symptoms Checklist – Youth to screen for mental health problems among Indigenous young people in custody	QLD	Youth detention	Mental health status<br>Substance dependence and substance-related harm	*N* = 47; *n* = 37 male; *n* = 10 female
Stathis et al. (2007) [421]	Describe the impact and implementation of Indigenous Health Workers in a service for mental health and substance use for youth in custody	QLD	Youth detention	Mental health status<br>Substance dependence and substance-related harm<br>Health service utilisation	*N* = 527 (sex/gender not reported)
Stathis et al. (2008) [422]	Determine the prevalence of mental health problems and needs among youth in detention	QLD	Youth detention	Mental health status<br>Substance dependence and substance-related harm	*N* = 164; *n* = 124 male; *n* = 40 female
Steele et al. (2021) [423]	Identify help-seeking behaviours associated with substance use among young people with contact with the justice system	QLD<br>WA	Youth detention<br>Other	Substance dependence and substance-related harm<br>Health service utilisation	*N* = 465; *n* = 293 male; *n* = 172 female
Stewart et al. (2021) [424]	Evaluate the prevalence of substance use, physical and mental health, and sociodemographic and criminogenic characteristics of men released from prison with a history of injecting drug use	VIC	Post-release<br>Prison	Mental health status<br>Substance dependence and substance-related harm<br>Bloodborne viruses and other communicable diseases<br>Non-communicable diseases<br>Physical health status<br>Health service utilisation	*N* = 336; 100% male
Stewart et al. (2021) [425]	Identify the relationship between incarceration and mental health diagnoses among people with history of contact with the justice system	QLD	Prison<br>Youth detention<br>Other	Mental health status<br>Cognitive disabilities<br>Substance dependence and substance-related harm<br>Health service utilisation	*N* = 44952; 45.8% male
Stewart et al. (2021) [426]	Describe and report characteristics associated with attrition in the prison and transition health cohort	VIC	Post-release<br>Prison	Mental health status<br>Substance dependence and substance-related harm<br>Bloodborne viruses and other communicable diseases	*N* = 336; 100% male
Stewart et al. (2022) [427]	Explore changes in psychiatric wellbeing after release from prison among people with a history of injecting drug use	VIC	Post-release<br>Prison	Mental health status<br>Substance dependence and substance-related harm<br>Physical health status<br>Violence victimisation and injury<br>Health service utilisation	*N* = 326; 100% male

Author (year)	Study aim(s)	Location	Setting	Mapped health domains	Sample
Stewart et al. (2004) [428]	Compare the risk of death among people released from prison to the general WA population	WA	Post-release<br>Prison	Mental health status<br>Substance dependence and substance-related harm<br>Non-communicable diseases<br>Physical health status	*N* = 9351; *n* = 8199 male; *n* = 1152 female
Stewart et al. (2018) [429]	Determine the prevalence of non-suicidal self-injury and suicidal behaviour among men experiencing incarceration with a history of injecting drugs	VIC	Prison	Mental health status<br>Cognitive disabilities<br>Substance dependence and substance-related harm<br>Physical health status<br>Health service utilisation	*N* = 364; 100% male
Stoové et al. (2008) [430]	Examine the mortality of people who inject drugs and characteristics that are associated with mortality	VIC	Community<br>Prison history	Substance dependence and substance-related harm	*N* = 220; *n* = 124 male; *n* = 96 female
Strand et al. (2016) [431]	Investigate the relationship between psychopathology, behaviour, and emotional functioning among young females in detention	VIC	Youth detention	Mental health status<br>Cognitive disabilities<br>Violence victimisation and injury	*N* = 40; 100% female
Sturman and Saiepour (2017) [432]	Identify eligibility for and attitudes towards opioid substitution therapy among people recently released from prison	QLD	Post-release	Substance dependence and substance-related harm<br>Health service utilisation	*N* = 140; 100% male
Sullivan et al. (2021) [433]	Report the prevalence of hepatitis B and C infection and testing among people incarcerated in NT prisons between 2003 and 2017	NT	Prison	Bloodborne viruses and other communicable diseases	Sample size and characteristics not reported
Sullivan et al. (2024) [434]	Evaluate the impact of the 'Connections' program on recidivism and health outcomes for people with a history of opioid use after release from prison	NSW	Post-release<br>Prison	Substance dependence and substance-related harm<br>Physical health status<br>Health service utilisation	*N* = 5549; *n* = 4609 male; *n* = 940 female
Sullivan et al. (2019) [435]	Determine the social, emotional, and physical well-being of Aboriginal mothers experiencing incarceration in prison	NSW<br>WA	Prison	Mental health status<br>Substance dependence and substance-related harm<br>Physical health status<br>Health service utilisation	*N* = 161; 100% female
Taflan et al. (2023) [436]	Understanding the sexual health needs of Aboriginal and non-Aboriginal adolescents in contact with the justice system	QLD<br>WA	Youth detention	Substance dependence and substance-related harm<br>Sexual and reproductive health<br>Violence victimisation and injury<br>Health service utilisation	*N* = 463; *n* = 292 male; *n* = 171 female
Tambakis et al. (2023) [437]	Examine the management of people presenting to hospital for foreign body ingestion	VIC	Prison	Mental health status<br>Substance dependence and substance-related harm<br>Health service utilisation	*N* = 63<br>Prison cohort: *n* = 36; *n* = 29 male; *n* = 7 female
Tan et al. (2023) [438]	Examine offending behaviour and characteristics associated among young people with foetal alcohol spectrum disorder	WA	Youth detention<br>Other	Cognitive disabilities	*N* = 100; 82% male; 18% female

Author (year)	Study aim(s)	Location	Setting	Mapped health domains	Sample
Tatkovis et al. (2023) [439]	Explore emergency department presentation rates and compare based on remoteness	National	Prison<br>Other	Mental health status<br>Substance dependence and substance-related harm<br>Violence victimisation and injury<br>Health service utilisation	*N* = 8352192<br>Police/correctional service vehicles sample: 55189; sex/gender not reported
Taylor et al. (2018) [440]	Model the costs of replacing prisoner transportation with a telehealth service	QLD	Prison	Health service utilisation	Sample size and characteristics not reported
Templeton et al. (2010) [441]	Determine the prevalence and risk factors associated with undiagnosed and asymptomatic sexually transmitted infections and bloodborne viruses among youth in detention	NSW	Youth detention	Substance dependence and substance-related harm<br>Bloodborne viruses and other communicable diseases<br>Sexual and reproductive health<br>Health service utilisation	*N* = 101; 100% male
Teutsch et al. (2010) [442]	Measure the incidence and risk factors associated with hepatitis C infection among people incarcerated in prisons and inject drugs	NSW	Prison	Substance dependence and substance-related harm<br>Bloodborne viruses and other communicable diseases<br>Violence victimisation and injury<br>Health service utilisation	*N* = 488; *n* = 318 male
Thein et al. (2006) [443]	Investigated the impact of hepatitis C infection on the health-related quality of life among people in prisons	NSW	Prison	Mental health status<br>Substance dependence and substance-related harm<br>Bloodborne viruses and other communicable diseases<br>Non-communicable diseases<br>Health service utilisation	*N* = 690 (sex/gender not reported)
Thomas et al. (2016) [444]	Identify trends in psychological distress levels after release from prison and examine the influence of mental health service utilisation on psychological distress levels	QLD	Post-release<br>Prison	Mental health status<br>Substance dependence and substance-related harm<br>Health service utilisation	*N* = 1247; *n* = 261 females)
Thomas et al. (2015) [445]	Identify health-related factors that predict recidivism among people with a history of incarceration	QLD	Post-release<br>Prison	Mental health status<br>Substance dependence and substance-related harm<br>Non-communicable diseases<br>Physical health status<br>Health service utilisation	*N* = 1319; *n* = 1040 male; *n* = 249 female
Thomas et al. (2022) [446]	Identify the association between accessing mental health and substance use services and risk of reincarceration	QLD	Prison	Mental health status<br>Substance dependence and substance-related harm<br>Health service utilisation	*N* = 1115; 20.5% female
Thomas et al. (2014) [447]	Determine the predictive validity of the Alcohol Use Disorders Identification Test among adults released from prison and explore post-release predictors of alcohol misuse	QLD	Post-release<br>Prison	Mental health status<br>Substance dependence and substance-related harm	*N* = 1296; 78.9% male
Thompson et al. (1998) [448]	Identify the vaccination status for hepatitis B for young people in custody. Determine the risk of hepatitis B infection and predictors of vaccine uptake	VIC	Youth detention	Substance dependence and substance-related harm<br>Bloodborne viruses and other communicable diseases<br>Health service utilisation	*N* = 90; 100% male
Thompson et al. (1998) [449]	Assess the serostatus for various vaccine-preventable diseases among youth in custody	VIC	Youth detention	Substance dependence and substance-related harm<br>Bloodborne viruses and other communicable diseases<br>Health service utilisation	*N* = 90; 100% male

Author (year)	Study aim(s)	Location	Setting	Mapped health domains	Sample
Treloar et al. (2023) [450]	Explore substance use and justice involvement among people with histories of child sexual abuse, and their experience of trauma-informed care	National	Prison<br>Other	Mental health status<br>Cognitive disabilities<br>Substance dependence and substance-related harm<br>Violence victimisation and injury	*N* = 172; *n* = 118 male; *n* = 53 female
Treloar et al. (2016) [451]	Explore Indigenous Australian's experience and behaviours related to hepatitis C care and treatment	NSW	Community<br>Prison history	Bloodborne viruses and other communicable diseases<br>Health service utilisation	*N* = 39; *n* = 23 male; *n* = 15 female
Trofimovs and Dowse (2014) [452]	Determine patterns of justice involvement among Indigenous males	NSW	Prison	Mental health status<br>Cognitive disabilities<br>Substance dependence and substance-related harm<br>Health service utilisation	*N* = 131; 100% male
Trofimovs et al. (2023) [453]	Evaluate the effect of post-release disability and community support services on reincarceration for people with intellectual disability and mental health diagnoses	NSW	Post-release<br>Prison	Mental health status<br>Cognitive disabilities<br>Health service utilisation	*N* = 484; *n* = 391 male; *n* = 93 females
Trofimovs et al. (2021) [454]	Estimate the prevalence and patterns of intellectual disability among people incarcerated in prisons	NSW	Prison	Cognitive disabilities	*N* = 457; *n* = 439 male
Trofimovs et al. (2022) [455]	Explore the association between disability support and reincarceration among people with intellectual disability who are incarcerated in prison	NSW	Prison	Cognitive disabilities<br>Health service utilisation	*N* = 1129; *n* = 1101 male
Trotter and Baidawi (2015) [456]	Describe the challenges and needs among older people incarcerated in prisons	NSW<br>VIC	Prison	Mental health status<br>Substance dependence and substance-related harm<br>Physical health status<br>Health service utilisation	*N* = 233; 100% male
Trotter et al. (2012) [457]	Evaluate the effective characteristics of prison-based and post-release support and rehabilitation services for women who experience incarceration	VIC	Post-release<br>Prison	Mental health status<br>Substance dependence and substance-related harm<br>Health service utilisation	*N* = 58; 100% female
Tye and Mullen (2006) [458]	Compare rates of mental disorder among women in prisons to that of the general community	VIC	Prison	Mental health status<br>Substance dependence and substance-related harm	*N* = 103; 100% female
Valerio et al. (2022) [459]	Identify the change in prevalence and treatment engagement for hepatitis C between 2018-2019 and 2019-2021 among people who inject drugs	NSW<br>QLD<br>WA<br>SA	Community<br>Prison history	Substance dependence and substance-related harm<br>Bloodborne viruses and other communicable diseases<br>Health service utilisation	*N* = 2395; *n* = 1591 male; *n* = 786 female; *n* = 18 other gender
Valerio et al. (2023) [460]	Explore the factors influencing HCT treatment uptake among females who inject drugs	WA	Prison<br>Other	Substance dependence and substance-related harm<br>Bloodborne viruses and other communicable diseases<br>Health service utilisation	*N* = 6563; 100% female
Valerio et al. (2021) [461]	Evaluate the prevalence of hepatitis C infection and treatment utilisation among people who inject drugs	NSW<br>QLD<br>SA<br>WA	Community<br>Prison history	Substance dependence and substance-related harm<br>Bloodborne viruses and other communicable diseases<br>Health service utilisation	*N* = 1443; *n* = 932 male; *n* = 508 female; *n* = 3 transgender
van Beek et al. (1998) [462]	Estimate the incidence of hepatitis C and HIV and risk factors associated with infection among people who inject drugs	NSW	Community<br>Prison history	Bloodborne viruses and other communicable diseases<br>Health service utilisation	*N* = 563; *n* = 244 male; *n* = 319 female

Author (year)	Study aim(s)	Location	Setting	Mapped health domains	Sample
van der Poorten et al. (2008) [463]	Examine the prevalence, risk factors, and knowledge of hepatitis C transmission among young people in custody and compare difference between Aboriginal and non-Aboriginal young people	NSW	Youth detention	Bloodborne viruses and other communicable diseases	*N* = 709; *n* = 618 male
van Dooren et al. (2011) [464]	Explore and understand the health of people released from prison for two or more years	QLD	Post-release<br>Prison	Substance dependence and substance-related harm	*N* = 100; *n* = 70 male; *n* = 30 female
van Dooren et al. (2010) [465]	Compare the health and health-related needs of people incarcerated in prisons between age groups (18-24 years, 25-40 years, and >40 years)	NSW	Prison	Mental health status<br>Substance dependence and substance-related harm<br>Bloodborne viruses and other communicable diseases<br>Non-communicable diseases<br>Sexual and reproductive health	*N* = 914; 18.3% female
van Dooren et al. (2013) [466]	Explore differences in prevalence and mortality between people aged <25 years and >25 years after release from prison	QLD	Post-release<br>Prison	Mental health status<br>Substance dependence and substance-related harm<br>Bloodborne viruses and other communicable diseases<br>Non-communicable diseases<br>Violence victimisation and injury<br>Health service utilisation	*N* = 42015; *n* = 37039 male; *n* = 4976 female
van Dooren et al. (2014) [467]	Examine risk behaviours and predictors of exposure to hepatitis C and compare differences between <25-year and >25-year groups of people incarcerated in prisons	QLD	Prison	Substance dependence and substance-related harm<br>Bloodborne viruses and other communicable diseases	*N* = 677; 76.3% male
Walker (2002) [468]	Compare levels of dissociation between young people in custody and a matched community cohort	NSW	Youth detention<br>Other	Mental health status	Custody cohort: *n* = 29; 100% male
Walker et al. (2014) [469]	Investigate the maternal and perinatal outcomes of women incarcerated in prisons compared to community controls	NSW	Prison	Mental health status<br>Substance dependence and substance-related harm<br>Non-communicable diseases<br>Sexual and reproductive health<br>Health service utilisation	prison cohort: *n* = 302; 100% female
Walker et al. (2016) [470]	Explore the genotype distribution and multiple hepatitis C infection among people experiencing incarceration who inject drugs	NSW	Prison	Substance dependence and substance-related harm<br>Bloodborne viruses and other communicable diseases	*N* = 123; *n* = 79 male
Walker et al. (2018) [471]	Examine life trajectories after release from prison for young males with a history of injecting drug use	VIC	Post-release<br>Prison	Mental health status<br>Substance dependence and substance-related harm<br>Violence victimisation and injury<br>Health service utilisation	*N* = 28; 100% male
Walker et al. (2020) [472]	Evaluate prison drug policy and capture the lived experiences of young people incarcerated in prisons who inject drugs	VIC	Prison	Substance dependence and substance-related harm<br>Bloodborne viruses and other communicable diseases<br>Health service utilisation	*N* = 28; 100% male
Wallis et al. (2023) [473]	Evaluate point-of-care RNA testing for hepatitis C within Australian prisons	QLD	Prison	Substance dependence and substance-related harm<br>Bloodborne viruses and other communicable diseases<br>Health service utilisation	*N* = 174; 100% female
Walsh (2022) [474]	Describe the health and demographic profiles of women who have died in custody between 1991 and 2020	National	Prison<br>Other	Mental health status<br>Substance dependence and substance-related harm<br>Non-communicable diseases	*N* = 34; 100% female

Author (year)	Study aim(s)	Location	Setting	Mapped health domains	Sample
Walsh and Counter (2019) [475]	Identify the demographic and criminal justice characteristics of people who died in custody between 1991 and 2016 and describe cause of death	National	Prison<br>Youth detention<br>Other	Mental health status<br>Substance dependence and substance-related harm<br>Physical health status<br>Violence victimisation and injury	*n* = 291 deaths in prison and youth detention; 95% male; 5% female
Wand et al. (2012) [476]	Assess the development and validation of a scoring tool to assess risk of hepatitis C infection for people who inject drugs	National	Community<br>Prison history	Bloodborne viruses and other infectious diseases	*N* = 10662 (sex/gender not reported)
Wand et al. (2020) [477]	Evaluate the factors that impact smoking cessation among people incarcerated in prison	NSW<br>QLD	Prison	Mental health status<br>Substance dependence and substance-related harm<br>Physical health status	*N* = 425; 100% male
Watkins et al. (2009) [478]	Evaluate the effectiveness of testing for sexually transmitted infections and bloodborne viruses among people entering correctional facilities	WA	Prison<br>Youth detention	Bloodborne viruses and other communicable diseases<br>Sexual and reproductive health	*N* = 956; *n* = 410 adult male; *n* = 302 adult female; *n* = 122 juvenile male; *n* = 122 juvenile female
Watson et al. (1973) [479]	Determine the incidence of Australia antigen and antibody in men incarcerated in prison	NSW	Prison	Substance dependence and substance-related harm<br>Bloodborne viruses and other communicable diseases	*N* = 1394; 100% male
White et al. (2012) [480]	Examine hepatitis B susceptibility and infection among people who inject drugs	NSW	Community<br>Prison history	Substance dependence and substance-related harm<br>Bloodborne viruses and other infectious diseases<br>Health service utilisation	*N* = 227; *n* = 172 male; *n* = 55 female
White and Chant (2006) [481]	Measure the psychometric properties of a psychosis screening tool in a prison setting	QLD	Prison	Mental health status	*N* = 567; 100% male
White et al. (2006) [482]	Determine the prevalence and characteristics of people experiencing incarceration with psychotic disorder compared to a community sample	QLD	Prison	Mental health status<br>Substance dependence and substance-related harm<br>Health service utilisation	*n* = 744 prison cohort; 100% male
White and Boyer (1985) [483]	Explore the relationship between alcohol use and crime	TAS	Prison	Substance dependence and substance-related harm	*N* = 462; *n* = 440 male; *n* = 22 female
Whiteman et al. (1998) [484]	Estimate the prevalence and determinants of hepatitis A infection among men entering prison	QLD	Prison	Substance dependence and substance-related harm<br>Bloodborne viruses and other communicable diseases<br>Physical health status	*N* = 254; 100% male
Willoughby et al. (2021) [485]	Determine the incidence and predictors of violence-related deaths among people released from prison	QLD	Post-release<br>Prison	Mental health status<br>Substance dependence and substance-related harm<br>Physical health status<br>Violence victimisation and injury<br>Health service utilisation	*N* = 1,238; *n* = 977 male; *n* = 261 female
Willoughby et al. (2021) [486]	Determine violence-related mortality rates among people released from prisons and compare between age groups and Indigenous status	QLD	Post-release<br>Prison	Physical health status<br>Violence victimisation and injury	*N* = 41970; *n* = 36996 male; *n* = 4974 female
Willoughby et al. (2023) [487]	Identify violence-related mortality among young people with a history of contact with the justice system	QLD	Post-release<br>Youth detention	Physical health status<br>Violence victimisation and injury	*N* = 48647; *n* = 36753 male; *n* = 11894 female

Author (year)	Study aim(s)	Location	Setting	Mapped health domains	Sample
Willoughby et al. (2021) [488]	Explore the characteristics and toxicology of violence-related deaths among youth with a history of contact with the justice system	NSW	Youth detention	Physical health status<br>Violence victimisation and injury	*N* = 48670; *n* = 36773 male; *n* = 11897 female
Winter et al. (2013) [489]	Determine the incidence and prevalence of hepatitis B among people who inject drugs; explore correlates of vaccination uptake	VIC	Community<br>Prison history	Substance dependence and substance-related harm<br>Bloodborne viruses and other communicable diseases<br>Health service utilisation	*N* = 344; *n* = 266 male
Winter et al. (2019) [490]	Evaluate the effect of injecting drug use relapse, sociodemographic and criminogenic characteristics, health, and behaviour on reincarceration after release from prison for people with a history of injecting drugs	QLD	Post-release<br>Prison	Mental health status<br>Substance dependence and substance-related harm<br>Health service utilisation	*N* = 512; *n* = 390 male
Winter et al. (2015) [491]	Obtain incidence estimates for self-reported non-fatal overdose across three time points among people released from prisons who inject drugs	QLD	Prison	Mental health status<br>Substance dependence and substance-related harm	*N* = 1,051; *n* = 829 male
Winter et al. (2016) [492]	Assess the effectiveness of a nurse-led bloodborne virus and sexually transmitted infection testing and vaccination program for prison entrants	VIC	Prison	Bloodborne viruses and other communicable diseases<br>Health service utilisation	*N* = 565; *n* = 377 male; *n* = 188 female
Winter et al. (2016) [493]	Describe the prevalence of injecting drug use following release from prison and identify risk factors associated with resumption	QLD	Post-release<br>Prison	Mental health status<br>Substance dependence and substance-related harm	*N* = 533; *n* = 125 female
Wolk et al. (1990) [494]	Investigate the relationships between HIV status, risk taking behaviour, and demographic characteristics of Sydney-based people who use intravenous drugs	NSW	Community<br>Prison history	Substance dependence and substance-related harm<br>Bloodborne viruses and other communicable diseases	*N* = 181; 64% male; 32% female; 4% transgender
Wu et al. (2023) [495]	Determine the obligations of providers and problems associated with emergency healthcare provision in prisons through reviewing coronial cases	VIC<br>NSW<br>QLD	Prison	Physical health status<br>Health service utilisation	Sample size and characteristics not reported
Yap et al. (2007) [496]	Explore the effect of condom distribution on sexual assault in prisons	NSW	Prison	Violence victimisation and injury	*N* = 1046; *n* = 747 male; *n* = 299 female
Yap et al. (2013) [497]	Measure the prevalence and factors associated with penile implants among people incarcerated in prisons	NSW<br>QLD	Prison	Bloodborne viruses and other communicable diseases<br>Sexual and reproductive health	*N* = 2,018; 100% male
Yap et al. (2011) [498]	Describe changes in the occurrence of sexual assaults in male prisons	NSW	Post-release<br>Prison	Substance dependence and substance-related harm<br>Violence victimisation and injury	*N* = 40; *n* = 33 male; *n* = 7 transgender
Yee et al. (2022) [499]	Identify the clinical stages of psychosis among people incarcerated in prison and referred to mental health services	NSW	Prison	Mental health status<br>Cognitive disabilities<br>Substance dependence and substance-related harm<br>Health service utilisation	*N* = 105; 100% male

Author (year)	Study aim(s)	Location	Setting	Mapped health domains	Sample
Young et al. (2015) [500]	Explore the relationship between contact with primary care health providers within one-month post-release and level of health service engagement 6-months post-release	QLD	Post-release<br>Prison	Mental health status<br>Cognitive disabilities<br>Bloodborne viruses and other communicable diseases<br>Non-communicable diseases<br>Health service utilisation	*N* = 847; *n* = 658 male; *n* = 189 female
Young et al. (2020) [501]	Examine the prevalence and predictors of contact with mental health services after hospital discharge due to self-harm among people released from prison	QLD	Prison	Mental health status<br>Cognitive disabilities<br>Substance dependence and substance-related harm<br>Health service utilisation	*N* = 217; *n* = 52 female
Young et al. (2020) [502]	Measure the incidence of hospital contact due to injury and compare between age groups for people released from prison	QLD	Prison	Mental health status<br>Cognitive disabilities<br>Substance dependence and substance-related harm<br>Physical health status<br>Violence victimisation and injury<br>Health service utilisation	*N* = 1307; *n* = 1030 male; *n* = 277 female
Young et al. (2017) [503]	Assess the relationship between intellectual disability and patient activation among people recently released from prison	QLD	Prison	Mental health status<br>Cognitive disabilities<br>Substance dependence and substance-related harm<br>Bloodborne viruses and other communicable diseases	*N* = 936; *n* = 731 male; *n* = 205 female
Young et al. (2018) [504]	Explore the association between dual diagnosis, mental illness, and substance use among people recently released from custody	QLD	Prison	Mental health status<br>Cognitive disabilities<br>Substance dependence and substance-related harm<br>Health service utilisation	*N* = 1307; *n* = 1030 male; *n* = 277 female
Young et al. (2015) [505]	Estimate the reliability of the Hayes Ability Screening Index among a sample of people incarcerated in prisons	WA	Prison	Cognitive disabilities	*N* = 190; *n* = 170 male; *n* = 20 female
Young et al. (2005) [506]	Describe an outbreak of influenza in an NSW prison	NSW	Prison	Bloodborne viruses and other communicable diseases	*N* = 37 (sex/gender not reported)
Young et al. (2005) [507]	Explore the health of women in Queensland prisons compared to the general community	QLD	Prison	Mental health status<br>Substance dependence and substance-related harm<br>Non-communicable diseases<br>Physical health status<br>Sexual and reproductive health	*N* = 212; 100% female
Yousafzai et al. (2022) [508]	Assess the trajectory of hepatitis C care and the factors that affect treatment utilisation and compare between 2011-2015 and 2016-2018	NSW	Community<br>Prison history	Substance dependence and substance-related harm<br>Bloodborne viruses and other communicable diseases	*N* = 166,276; *n* = 62% male
Zilkens et al. (2018) [509]	Explore the demographic and injury characteristics among men who have experienced sexual assault	WA	Prison	Violence victimisation and injury	*N* = 103; 100% male

Supplementary Table 6: Number of included studies by sample setting and location

Australian State or Territory	Prison n (%)	Youth Detention n (%)	Post-Release n (%)	All Settings n (%)
New South Wales	158 (53%)	30 (41%)	37 (32%)	224 (46%)
Queensland	74 (25%)	9 (12%)	52 (45%)	134 (28%)
Victoria	56 (19%)	11 (15%)	25 (22%)	92 (19%)
Western Australia	25 (8%)	13 (18%)	13 (11%)	50 (10%)
South Australia	17 (6%)	10 (14%)	7 (6%)	34 (7%)
Northern Territory	7 (2%)	2 (3%)	2 (2%)	11 (2%)
Tasmania	8 (3%)	1 (1%)	1 (1%)	10 (2%)
Australian Capital Territory	6 (2%)	0 (0%)	0 (0%)	6 (1%)
**Geographical Distribution [a]**	**298 (62%)**	**73 (15%)**	**116 (24%)**	**484 (100%)**
National	18 (6%)	1 (1%)	4 (3%)	22 (4%)
Not Reported	1 (<1%)	0 (0%)	1 (1%)	2 (<1%)
**Total Setting Distribution [b]**	**317 (63%)**	**74 (15%)**	**121 (24%)**	**508 (100%)**

[a] Percentages sum to greater than 100% because 39 included studies involved cohorts from multiple jurisdictions (prison, n=28; youth detention, n=3; post-release, n=8).
[b] Percentages sum to greater than 100% because 4 included studies identified people incarcerated or previously incarcerated in both prison and youth detention.

Supplementary Table 7: Proportion of data reported across health domains for distinct cohorts—prison (n=431)

Project name, author(s), and year	Total studies (n=431)	Substance dependence and substance-related harm (n=301)	Mental health status (n=231)	Health service utilisation (n=180)	Bloodborne viruses and other communicable diseases (n=150)	Physical health status (n=104)	Non-communicable diseases (n=67)	Violence victimisation and injury (n=61)	Cognitive disabilities (n=58)	Sexual and reproductive health (n=29)
	**n (%)**	**n (%)**	**n (%)**	**n (%)**	**n (%)**	**n (%)**	**n (%)**	**n (%)**	**n (%)**	**n (%)**
Passports Study[274]	49 (11%)	41 (14%)	43 (19%)	31 (17%)	16 (11%)	14 (13%)	9 (13%)	3 (5%)	15 (26%)	3 (10%)
2001 NSW Inmate Health Survey[510]	20 (5%)	12 (4%)	14 (6%)	2 (1%)	8 (5%)	6 (6%)	7 (10%)	1 (2%)	2 (3%)	3 (10%)
PATH cohort study[278]	13 (3%)	13 (4%)	10 (4%)	11 (6%)	5 (3%)	6 (6%)	3 (4%)	2 (3%)	2 (3%)	0 (0%)
HITS-p study[177]	10 (2%)	9 (3%)	0 (0%)	3 (2%)	10 (7%)	0 (0%)	0 (0%)	5 (8%)	0 (0%)	0 (0%)
1996 NSW Inmate Health Survey[511]	10 (2%)	5 (2%)	3 (1%)	2 (1%)	7 (5%)	4 (4%)	4 (6%)	0 (0%)	1 (2%)	2 (7%)
SHAAP study[68]	6 (1%)	1 (<1%)	1 (<1%)	0 (0%)	2 (1%)	1 (1%)	0 (0%)	3 (5%)	0 (0%)	3 (10%)
2009 NSW Inmate Health Survey[512]	6 (1%)	4 (1%)	3 (1%)	1 (1%)	3 (2%)	3 (3%)	4 (6%)	0 (0%)	2 (3%)	1 (3%)
TBI among Australian prisoners[392]	5 (1%)	4 (1%)	4 (2%)	2 (1%)	0 (0%)	1 (1%)	0 (0%)	2 (3%)	5 (9%)	0 (0%)
Australian prisons and Indigenous people's needs[397]	5 (1%)	3 (1%)	5 (2%)	1 (1%)	0 (0%)	1 (1%)	0 (0%)	0 (0%)	3 (5%)	0 (0%)
**Total**	**111 (26%)**	**92 (31%)**	**83 (36%)**	**53 (29%)**	**51 (34%)**	**36 (35%)**	**27 (40%)**	**16 (26%)**	**30 (52%)**	**12 (41%)**

Note: Total studies amount to less due to 9 studies that used data from more than one listed cohort study: 1996 and 2001 NSW Inmate Health Survey (n=2); 1996, 2001 and 2009 NSW Inmate Health Survey (n=4); 2001 and 2009 NSW Inmate Health Surveys (n=2); Passports Study and 2001 NSW Inmate Health Survey (n=1).

Supplementary Table 8: Proportion of data reported across domains for distinct cohorts—youth detention (n=83)

Project name, author(s), and year	Total studies (n=83)	Substance dependence and substance-related harm (n=52)	Mental health status (n=51)	Cognitive disabilities (n=24)	Violence victimisation and injury (n=23)	Health service utilisation (n=19)	Physical health status (n=18)	Bloodborne viruses and other communicable diseases (n=12)	Non-communicable diseases (n=5)	Sexual and reproductive health (n=4)
	n (%)	n (%)	n (%)	n (%)	n (%)	n (%)	n (%)	n (%)	n (%)	n (%)
2009 NSW Young People in Custody Survey[513]	6 (7%)	5 (10%)	6 (13%)	3 (13%)	2 (9%)	1 (5%)	1 (6%)	0 (0%)	0 (0%)	0 (0%)
Foetal alcohol spectrum disorder prevalence study[46]	6 (7%)	0 (0%)	0 (0%)	6 (25%)	0 (0%)	1 (5%)	2 (11%)	0 (0%)	0 (0%)	0 (0%)
Juvenile offenders and hepatitis B risk[449]	3 (4%)	3 (6%)	0 (0%)	0 (0%)	0 (0%)	2 (11%)	1 (6%)	3 (25%)	0 (0%)	0 (0%)
Adolescents in custody: Hidden psychological morbidity? [283]	2 (2%)	1 (2%)	2 (4%)	0 (0%)	0 (0%)	0 (0%)	0 (0%)	0 (0%)	0 (0%)	0 (0%)
Juvenile offender sexual health survey[423]	2 (2%)	2 (4%)	0 (0%)	0 (0%)	1 (4%)	2 (11%)	0 (0%)	0 (0%)	0 (0%)	1 (25%)
2003 NSW young people in custody survey[514]	2 (2%)	1 (2%)	2 (4%)	0 (0%)	2 (9%)	0 (0%)	1 (6%)	0 (0%)	0 (0%)	0 (0%)
Alcohol abuse among young offenders[363]	2 (2%)	2 (4%)	0 (0%)	0 (0%)	0 (0%)	0 (0%)	0 (0%)	0 (0%)	0 (0%)	0 (0%)
Mortality in young offenders[92]	2 (2%)	2 (4%)	2 (4%)	0 (0%)	2 (9%)	0 (0%)	2 (11%)	1 (8%)	1 (20%)	0 (0%)
Complex health needs in the youth justice system[271]	2 (2%)	2 (4%)	2 (4%)	0 (0%)	2 (9%)	0 (0%)	1 (6%)	0 (0%)	0 (0%)	0 (0%)
Correlates of self-harm and suicide attempts[396]	2 (2%)	1 (2%)	2 (4%)	0 (0%)	1 (4%)	1 (5%)	0 (0%)	0 (0%)	0 (0%)	0 (0%)
**Total**	**28 (34%)**	**19 (37%)**	**16 (31%)**	**9 (38%)**	**10 (43%)**	**7 (37%)**	**8 (44%)**	**4 (33%)**	**1 (20%)**	**1 (25%)**

Note. Total studies amount to less due to one study that used data from both the 2003 and 2009 NSW young people in custody survey.

References

1. Carter A, Butler A, Willoughby M, Janca E, Kinner SA, Southalan L, et al. Interventions to reduce suicidal thoughts and behaviours among people in contact with the criminal justice system: A global systematic review. eClinicalMedicine. 2022;44:Article 101266.
2. Abbott P, Davison J, Magin PJ, Hu W. 'If they're your doctor, they should care about you': Women on release from prison and general practitioners. Aust Fam Physician. 2016;45(10):728-32.
3. Abbott P, Magin P, Wendy H. Healthcare delivery for women in prison: A medical record review. Aust J Prim Health. 2016;22(6):523-9.
4. Abbott P, Magin P, Davison J, Hu W. Medical homelessness and candidacy: Women transiting between prison and community health care. Int J Equity Health. 2017;16:1-10.
5. Abbott P, Magin P, Lujic S, Hu W. Supporting continuity of care between prison and the community for women in prison: A medical record review. Aust Health Rev. 2017;41(3):268-76.
6. Abbott P, Watt K, Magin P, Davison J, Hu WCY. Welcomeness for people with substance use disorders to general practice: A qualitative study. Fam Pract. 2022;39(2):257-63.
7. Adams J, Ellis A, Brown A, Owens D, Halsey R. A prison mental health screening unit: A first for New South Wales. Australas Psychiatry. 2009;17(2):90-6.
8. Aitken C, Delalande C, Stanton K. Pumping iron, risking infection? Exposure to hepatitis C, hepatitis B and HIV among anabolic-androgenic steroid injectors in Victoria, Australia. Drug Alcohol Depend. 2002;65(3):303-8.
9. Alan J, Burmas M, Preen D, Pfaff J. Inpatient hospital use in the first year after release from prison: A Western Australian population-based record linkage study. Aust N Z J Public Health. 2011;35(3):264-9.
10. Allnutt S, Wedgwood L, Wilhelm K, Butler T. Temperament, substance use and psychopathology in a prisoner population: Implications for treatment. Aust N Z J Psychiatry. 2008;42(11):969-75.
11. Amarasena N, Kapellas K, Skilton MR, Maple-Brown LJ, Brown A, O'Dea K, et al. Associations with dental caries experience among a convenience sample of Aboriginal Australian adults. Aust Dent J. 2015;60(4):471-8.
12. Andrews JY, Forsyth S, Wade J, Kinner SA. Sensitivity of a national coronial database for monitoring unnatural deaths among ex-prisoners in Australia. BMC Res Notes. 2011;4:450.
13. Andrews JY, Kinner SA. Understanding drug-related mortality in released prisoners: A review of national coronial records. BMC Public Health. 2012;12(1):270.
14. Ashdown LR, Kilvert GT. Granuloma inguinale in Northern Queensland. Med J Aust. 1979;1(5):146-8.
15. Aung P, Goutzamanis S, Douglass C, Stoove M, Hellard M, Dietze P, et al. Exploring opportunities for hepatitis C treatment uptake among people who inject drugs in Australia: A qualitative study. J Subst Use. 2023.
16. Austin AE, van den Heuvel C, Byard RW. Prison suicides in South Australia: 1996-2010. J Forensic Sci. 2014;59(5):1260-2.
17. Awofeso N, Fennell M, Waliuzzaman Z, O'Connor C, Pittam D, Boonwaat L, et al. Influenza outbreak in a correctional facility. Aust N Z J Public Health. 2001;25(5):443-6.
18. Awofeso N, Levy M, Harper S, Jones M, Hayes M, Douglas J, et al. Response to HBV vaccine in relation to vaccine dose and anti-HCV positivity: A New South Wales correctional facilities' study. Vaccine. 2001;19(30):4245-8.
19. Baidawi S. Older prisoners: Psychological distress and associations with mental health history, cognitive functioning, socio-demographic, and criminal justice factors. Int Psychogeriatr. 2016;28(3):385-95.
20. Baidawi S, Trotter C. Psychological distress among older prisoners. J Correct Health Care. 2016;22(4):354-66.
21. Baidawi S, Trotter C, Flynn C. Prison experiences and psychological distress among older inmates. J Gerontol Soc Work. 2016;59(3):252-70.
22. Baidawi S, Trotter C, O'Connor DW. An integrated exploration of factors associated with psychological distress among older prisoners. J Forens Psychiatry Psychol. 2016;27(6):815-34.
23. Bajis S, Grebely J, Cooper L, Smith J, Owen G, Chudleigh A, et al. Hepatitis C virus testing, liver disease assessment and direct-acting antiviral treatment uptake and outcomes in a service for people who are homeless in Sydney, Australia: The LiveRLife homelessness study. J Viral Hepat. 2019;26(8):969-79.
24. Baldry E, Clarence M, Dowse L, Trollor J. Reducing vulnerability to harm in adults with cognitive disabilities in the Australian criminal justice system. J Policy Pract Intellect Disabil. 2013;10(3):222-9.
25. Barling J, Halpin R, Levy M. Capturing perceptions: Prisoners assess their health services - Australia, 2001 and 2004. Int J Prison Health. 2005;1(2):183-98.

26. Barrett EL, Indig D, Sunjic S, Sannibale C, Sindicich N, Rosenfeld J, et al. Treating comorbid substance use and traumatic stress among male prisoners: A pilot study of the acceptability, feasibility, and preliminary efficacy of seeking safety. Int J Forensic Ment Health. 2015;14(1):45.
27. Bartholomew AA, Brain LA, Douglas AS, Reynolds WS. A medico-psychiatric diagnostic review of remanded (without a request for a psychiatric report) male minor offenders. Med J Aust. 1967;1(6):267-9.
28. Bartlett SR, Jacka B, Bull RA, Luciani F, Matthews GV, Lamoury FMJ, et al. HIV infection and hepatitis C virus genotype 1a are associated with phylogenetic clustering among people with recently acquired hepatitis C virus infection. Infect Genet Evol. 2016;37:252-8.
29. Barton J, Cumming SR, Samuels A, Meade T. Common and distinguishing historical, criminal and current environmental and psychological characteristics in male inmates with a history of suicidal and/or non-suicidal self-injury. J Crim Psychol. 2017;7(4):229-43.
30. Barton JJ, Meade T, Cumming S, Samuels A. Predictors of self-harm in male inmates. J Crim Psychol. 2014;4(1):2.
31. Bate JP, Colman AJ, Frost PJ, Shaw DR, Harley HAJ. High prevalence of late relapse and reinfection in prisoners treated for chronic hepatitis C. J Gastroenterol Hepatol. 2010;25(7):1276-80.
32. Batey RG, Jones T, McAllister C. Prisons and HCV: A review and a report on an experience in New South Wales Australia. Int J Prison Health. 2008;4(3):156-63.
33. Belcher JM, Butler T, Richmond RL, Wodak AD, Wilhelm K. Smoking and its correlates in an Australian prisoner population. Drug Alcohol Rev. 2006;25(4):343-8.
34. Bell MF, Kelty E, Segal L, Dennison S, Kinner SA, Dawe S, et al. Neonatal abstinence syndrome and other neonatal outcomes for the infants of women experiencing incarceration: A retrospective cohort study. Aust J Soc Issues. 2023.
35. Bhandari A, van Dooren K, Eastgate G, Lennox N, Kinner SA. Comparison of social circumstances, substance use and substance-related harm in soon-to-be-released prisoners with and without intellectual disability. J Intellect Disabil Res. 2015;59(6):571-9.
36. Bickel R, Campbell A. Mental health of adolescents in custody: The use of the "Adolescent Psychopathology Scale" in a Tasmanian context. Aust N Z J Psychiatry. 2002;36(5):603-9.
37. Biles D. Deaths in private and public prisons in Australia: A comparative analysis. Australian and New Zealand Journal of Criminology. 2001;34(3):293-301.
38. Binswanger IA, Blatchford PJ, Forsyth SJ, Stern MF, Kinner SA. Epidemiology of infectious disease-related death after release from prison, Washington State, United States, and Queensland, Australia: A cohort study. Public Health Rep. 2016;131(4):574-82.
39. Boonwaat L, Haber PS, Levy MH, Lloyd AR. Establishment of a successful assessment and treatment service for Australian prison inmates with chronic hepatitis C. Med J Aust. 2010;192(9):496-500.
40. Borschmann R, Coffey C, Moran P, Hearps S, Degenhardt L, Kinner SA, et al. Self-harm in young offenders. Suicide Life Threat Behav. 2014;44(6):641-52.
41. Borschmann R, de Andrade D, Kinner SA. Health and welfare outcomes for adolescents following release from prison in Queensland, Australia: A prospective cohort study. Adolescents. 2021;1(2):175.
42. Borschmann R, dos Santos MM, Young JT, Andreoli SB, Love A, er D, et al. Health, social and criminal justice factors associated with dual diagnosis among incarcerated adults in Brazil and Australia: A cross-national comparison. Soc Psychiatry Psychiatr Epidemiol. 2020;55(10):1355-62.
43. Borschmann R, Thomas E, Moran P, Carroll M, Heffernan E, Spittal MJ, et al. Self-harm following release from prison: A prospective data linkage study. Aust N Z J Psychiatry. 2017;51(3):250-9.
44. Borschmann R, Young JT, Moran P, Spittal MJ, Heffernan E, Mok K, et al. Ambulance attendances resulting from self-harm after release from prison: A prospective data linkage study. Soc Psychiatry Psychiatr Epidemiol. 2017;52(10):1295-305.
45. Borschmann R, Young JT, Moran P, Spittal MJ, Snow K, Mok K, et al. Accuracy and predictive value of incarcerated adults' accounts of their self-harm histories: Findings froman Australian prospective data linkage study. CMAJ Open. 2017;5(3):E694-E701.
46. Bower C, Watkins RE, Mutch RC, Marriott R, Freeman J, Kippin NR, et al. Fetal alcohol spectrum disorder and youth justice: A prevalence study among young people sentenced to detention in Western Australia. BMJ Open. 2018;8(2):Article e019605.
47. Bretaña NA, Boelen L, Bull R, Teutsch S, White PA, Lloyd AR, et al. Transmission of hepatitis C virus among prisoners, Australia, 2005-2012. Emerg Infect Dis. 2015;21(5):765-74.
48. Brömdal A, Sanders T, Stanners M, du Plessis C, Gildersleeve J, Mullens AB, et al. Where do incarcerated trans women prefer to be housed and why? Adding nuanced understandings to a complex debate through the voices of formerly incarcerated trans women in Australia and the United States. Int J Transgend Health. 2023.

49. Brothers TD, Lewer D, Jones N, Colledge-Frisby S, Bonn M, Wheeler A, et al. Effect of incarceration and opioid agonist treatment transitions on risk of hospitalisation with injection drug use-associated bacterial infections: A self-controlled case series in New South Wales, Australia. Int J Drug Policy. 2023;122:Article 104218.
50. Brown S, Day A. The role of loneliness in prison suicide prevention and management. J Offender Rehabil. 2008;47(4):433-49.
51. Browne CC, Korobanova D, Chemjong P, Harris AWF, Glozier N, Basson J, et al. Continuity of mental health care during the transition from prison to the community following brief periods of imprisonment. Front Psychiatry. 2022;13:934837.
52. Browne CC, Korobanova D, Yee N, Spencer S-J, Ma T, Butler T, et al. The prevalence of self-reported mental illness among those imprisoned in New South Wales across three health surveys, from 2001 to 2015. Aust N Z J Psychiatry. 2023;57(4):550-61.
53. Burrage L, Zimmerman H, Higgins S, Param K, Orme C, Mitchell J, et al. Performance of simple serum-based tests to exclude cirrhosis prior to hepatitis C treatment in non-hospital settings in Australia. Intern Med J. 2021;51(4):533-9.
54. Butler A, Love AD, Young JT, Kinner SA. Frequent attendance to the emergency department after release from prison: A prospective data linkage study. J Behav Health Serv Res. 2020;47(4):544-59.
55. Butler A, Young JT, Kinner SA, Borschmann R. Self-harm and suicidal behaviour among incarcerated adults in the Australian Capital Territory. Health Justice. 2018;6(13).
56. Butler T, Allnutt S, Cain D, Owens D, Muller C. Mental disorder in the New South Wales prisoner population. Australian and New Zealand Journal of Psychiatry. 2005;39(5):407-13.
57. Butler T, Allnutt S, Kariminia A, Cain D. Mental health status of Aboriginal and non-Aboriginal Australian prisoners. Australian and New Zealand Journal of Psychiatry. 2007;41(5):429-35.
58. Butler T, Allnutt S, Yang B. Mentally ill prisoners in Australia have poor physical health. Int J Prison Health. 2007;3(2):99-110.
59. Butler T, Andrews G, Allnutt S, Sakashita C, Smith NE, Basson J. Mental disorders in Australian prisoners: A comparison with a community sample. Australian and New Zealand Journal of Psychiatry. 2006;40(3):272-6.
60. Butler T, Belcher JM, Champion U, Kenny D, Allerton M, Fasher M. The physical health status of young Australian offenders. Aust N Z J Public Health. 2008;32(1):73-80.
61. Butler T, Boonwaat L, Hailstone S, Falconer T, Lems P, Ginley T, et al. The 2004 Australian prison entrants' blood-borne virus and risk behaviour survey. Aust N Z J Public Health. 2007;31(1):44-50.
62. Butler T, Donovan B, Taylor J, Cunningham AL, Mindel A, Levy M, et al. Herpes simplex virus type 2 in prisoners, New South Wales, Australia. Int J STD AIDS. 2000;11(11):743-7.
63. Butler T, Indig D, Allnutt S, Mamoon H. Co-occurring mental illness and substance use disorder among Australian prisoners. Drug Alcohol Rev. 2011;30(2):188-94.
64. Butler T, Kariminia A, Levy M, Kaldor J. Prisoners are at risk for hepatitis C transmission. Eur J Epidemiol. 2004;19(12):1119-22.
65. Butler T, Kariminia A, Levy M, Murphy M. The self-reported health status of prisoners in New South Wales. Aust N Z J Public Health. 2004;28(4):344-50.
66. Butler T, Levy M. Mantoux positivity among prison inmates--New South Wales, 1996. Aust N Z J Public Health. 1999;23(2):185-8.
67. Butler T, Levy M, Dolan K, Kaldor J. Drug use and its correlates in an Australian prisoner population. Addict Res Theory. 2003;11(2):89-101.
68. Butler T, Malacova E, Richters J, Yap L, Grant L, Richards A, et al. Sexual behaviour and sexual health of Australian prisoners. Sex Health. 2013;10(1):64-73.
69. Butler T, Robertson P, Kaldor J, Donovan B. Syphilis in New South Wales (Australia) prisons. Int J STD AIDS. 2001;12(6):376-9.
70. Butler T, Spencer J, Cui J, Vickery K, Zou J, Kaldor J. Seroprevalence of markers for hepatitis B, C and G in male and female prisoners--NSW, 1996. Aust N Z J Public Health. 1999;23(4):377-84.
71. Butler TG, Dolan KA, Ferson MJ, McGuinness LM, Brown PR, Robertson PW. Hepatitis B and C in New South Wales prisons: Prevalence and risk factors. Med J Aust. 1997;166(3):127-30.
72. Butler TG, Gullotta M, Greenberg D. Reliability of prisoners' survey responses: Comparison of self-reported health and biomedical data from an Australian prisoner cohort. BMC Public Health. 2022;22(64).
73. Calais-Ferreira L, Butler A, Dent S, Preen DB, Young JT, Kinner SA. Multimorbidity and quality of primary care after release from prison: A prospective data-linkage cohort study. BMC Health Serv Res. 2022;22(1):876.
74. Calais-Ferreira L, Young JT, Francis K, Willoughby M, Pearce L, Clough A, et al. Non-communicable disease mortality in young people with a history of contact with the youth justice system in

Queensland, Australia: A retrospective, population-based cohort study. Lancet Public Health. 2023;8(8):e600-e9.
75. Carlton B, Segrave M. Women's survival post-imprisonment: Connecting imprisonment with pains past and present. Punishm Soc. 2011;13(5):551-70.
76. Carlton B, Segrave M. 'They died of a broken heart': Connecting women's experiences of trauma and criminalisation to survival and death post-imprisonment. Howard J Crim Justice. 2014;53(3):270.
77. Carrington N, Conway A, Grebely J, Starr M, Catlett B, Stevens A, et al. Testing, diagnosis, and treatment following the implementation of a program to provide dried blood spot testing for HIV and hepatitis C infections: The NSW DBS Pilot. BMC Infect Dis. 2024;24:1-11.
78. Carroll M, Kinner SA, Heffernan EB. Medication use and knowledge in a sample of Indigenous and non-Indigenous prisoners. Aust N Z J Public Health. 2014;38(2):142-6.
79. Carroll M, Spittal MJ, Kemp-Casey AR, Lennox NG, Preen DB, Sutherland G, et al. High rates of general practice attendance by former prisoners: A prospective cohort study. Med J Aust. 2017;207(2):75-80.
80. Carroll M, Sutherland G, Kemp-Casey A, Kinner SA. Agreement between self-reported healthcare service use and administrative records in a longitudinal study of adults recently released from prison. Health Justice. 2016;4:11.
81. Carson JM, Dore GJ, Lloyd AR, Grebely J, Byrne M, Cunningham E, et al. Hepatitis C virus reinfection following direct-acting antiviral treatment in the prison setting: The SToP-C study. Clin Infect Dis. 2022;75(10):1809-19.
82. Carson JM, Hajarizadeh B, Hanson J, O'Beirne J, Iser D, Read P, et al. Effectiveness of treatment for hepatitis C virus reinfection following direct acting antiviral therapy in the REACH-C cohort. Int J Drug Policy. 2021;96.
83. Cashin A, Butler T, Levy M, Potter E. Intellectual disability in the New South Wales inmate population. Int J Prison Health. 2006;2(2):115-20.
84. Charlson F, Gynther B, Obrecht K, Heffernan E, David M, Young JT, et al. Incarceration among adults living with psychosis in Indigenous populations in Cape York and the Torres Strait. Aust N Z J Psychiatry. 2021;55(7):678-86.
85. Chong CAKY, Li S, Nguyen GC, Sutton A, Levy MH, Butler T, et al. Health-state utilities in a prisoner population: A cross-sectional survey. Health Qual Life Outcomes. 2009;7:78.
86. Chowdhury NZ, Wand H, Albalawi O, Adily A, Kariminia A, Allnutt S, et al. Mental health service contact following release from prison or hospital discharge in those with psychosis. Front Psychiatry. 2022;13:1034917.
87. Chowdhury NZ, Albalawi O, Wand H, Adily A, Kariminia A, Allnutt S, et al. First diagnosis of psychosis in the prison: Results from a data-linkage study. BJPsych Open. 2019;5(6):e89.
88. Chowdhury NZ, Albalawi O, Wand H, Allnutt S, Adily A, Azar K, et al. Psychosis and criminal offending: A population-based data-linkage study. Crim Justice Behav. 2021;48(2):157-74.
89. Clark KA, Bromdal A, Phillips T, Sanders T, Mullens AB, Hughto JM. Developing the "oppression-to-incarceration cycle" of Black American and First Nations Australian trans women: Applying the intersectionality research for transgender health justice framework. J Correct Health Care. 2023;29(1):27-38.
90. Clarke A, Haag K, Owen N. Fitness programs for prison inmates. ACHPER Natl J. 1986;112:11-4.
91. Cockram J. People with an intellectual disability in the prisons. Psychiatr Psychol Law. 2005;12(1):163-73.
92. Coffey C, Veit F, Wolfe R, Cini E, Patton GC. Mortality in young offenders: Retrospective cohort study. Br Med J. 2003;326(7398):1064-6.
93. Coffey C, Wolfe R, Lovett AW, Moran P, Cini E, Patton GC. Predicting death in young offenders: A retrospective cohort study. Med J Aust. 2004;181(9):473-7.
94. Coles T, Simpson P, Saulo D, Kaldor J, Richards A, Levy M, et al. Trends in hepatitis B prevalence and associated risk factors among Indigenous and non-Indigenous prison entrants in Australia, 2004 to 2013. Aust N Z J Public Health. 2019;43(3):236-40.
95. Conolly L, Potter F. Aids education in New South Wales prisons. Aust N Z J Criminol. 1990;23(3):158.
96. Conway A, Stevens A, Murray C, Prain B, Power C, McNulty A, et al. Hepatitis C treatment uptake following dried blood spot testing for hepatitis C RNA in New South Wales, Australia: The NSW DBS pilot study. Open Forum Infect Dis. 2023;10(11).
97. Conway A, Valerio H, Alavi M, Silk D, Treloar C, Hajarizadeh B, et al. A testing campaign intervention consisting of peer-facilitated engagement, point-of-care HCV RNA testing, and linkage to nursing support to enhance hepatitis C treatment uptake among people who inject drugs: The ETHOS Engage study. Viruses. 2022;14(7).

98. Copeland J, Howard J, Fleischmann S. Gender, HIV knowledge and risk taking behaviour among substance using adolescents in custody in New South Wales. J Subst Misuse. 1998;3(4):206-12.
99. Copeland J, Howard J, Keogh T, Seidler K. Patterns and correlates of substance use amongst juvenile detainees in New South Wales 1989-99. Drug Alcohol Rev. 2003;22(1):15-20.
100. Cossar R, Stoove M, Kinner SA, Dietze P, Aitken C, Curtis M, et al. The associations of poor psychiatric well-being among incarcerated men with injecting drug use histories in Victoria, Australia. Health Justice. 2018;6(1).
101. Cossar RD, Stewart AC, Wilkinson AL, Dietze P, Ogloff JRP, Aitken C, et al. Emergency department presentations in the first weeks following release from prison among men with a history of injecting drug use in Victoria, Australia: A prospective cohort study. Int J Drug Policy. 2022;101:103532.
102. Crissman B. Deaths of people with serious mental disorder: An exploration of deaths in custody and fatal police contacts. Aust J Soc Issues. 2019;54(3):245-66.
103. Crofts N, Cooper G, Stewart T, Kiely P, Coghlan P, Hearne P, et al. Exposure to hepatitis A virus among blood donors, injecting drug users and prison entrants in Victoria. J Viral Hepat. 1997;4(5):333-8.
104. Crofts N, Hopper JL, Bowden DS, Breschkin AM, Milner R, Locarnini SA. Hepatitis C virus infection among a cohort of Victorian injecting drug users. Med J Aust. 1993;159(4):237-41.
105. Crofts N, Stewart T, Hearne P, Ping XY, Breschkin AM, Locarnini SA. Spread of bloodborne viruses among Australian prison entrants. Br Med J. 1995;310(6975):285-8.
106. Crofts N, Thompson S. Risk behaviours for blood-borne viruses In a Victorian prison. Aust N Z J Criminol. 1996;29(1):20.
107. Cumming C, Kinner SA, McKetin R, Li I, Preen DB. The health needs of people leaving prison with a history of methamphetamine and/or opioid use. Drug Alcohol Rev. 2023;42(4):778-84.
108. Cumming C, Kinner SA, McKetin R, Young JT, Li I, Preen DB. Using the Alcohol, Smoking and Substance Involvement Screening Test to predict substance-related hospitalisation after release from prison: A cohort study. Addiction. 2024;119(2):236-47.
109. Cumming C, Kinner SA, McKetin R, Young JT, Li I, Preen DB. The predictive validity of the Alcohol, Smoking and Substance Involvement Screening Test (ASSIST) for moderate- to high-risk cannabis, methamphetamine and opioid use after release from prison. Addiction. 2023;118(6):1107-15.
110. Cunningham EB, Hajarizadeh B, Bretana NA, Amin J, Betz-Stablein B, Dore GJ, et al. Ongoing incident hepatitis C virus infection among people with a history of injecting drug use in an Australian prison setting, 2005-2014: The HITS-p study. J Viral Hepat. 2017;24(9):733-41.
111. Curtis M, Dietze P, Aitken C, Kirwan A, Kinner SA, Butler T, et al. Acceptability of prison-based take-home naloxone programmes among a cohort of incarcerated men with a history of regular injecting drug use. Harm Reduct J. 2018;15(1):48.
112. Curtis M, Dietze P, Wilkinson AL, Agius PA, Stewart AC, Cossar RD, et al. Discontinuation of opioid agonist treatment following release from prison in a cohort of men who injected drugs prior to imprisonment in Victoria, Australia: A discrete-time survival analysis. Drug Alcohol Depend. 2023;242:N.PAG-N.PAG.
113. Curtis M, Wilkinson AL, Dietze P, Stewart AC, Kinner SA, Cossar RD, et al. Prospective study of retention in opioid agonist treatment and contact with emergency healthcare following release from prisons in Victoria, Australia. Emerg Med J. 2023;40(5):347-54.
114. Curtis M, Wilkinson AL, Dietze P, Stewart AC, Kinner SA, Winter RJ, et al. Is use of opioid agonist treatment associated with broader primary healthcare use among men with recent injecting drug use histories following release from prison? A prospective cohort study. Harm Reduct J. 2023;20(1):42.
115. Cutcher Z, Degenhardt L, Alati R, Kinner SA. Poor health and social outcomes for ex-prisoners with a history of mental disorder: A longitudinal study. Aust N Z J Public Health. 2014;38(5):424-9.
116. D'Antoine M, Malvaso C, Delfabbro P, O'Connor J. Suicidal behaviour in Aboriginal and non-Aboriginal young men under custodial youth justice supervision: Understanding the role of adverse childhood experiences. Psychiatr Psychol Law. 2022;29(6):953-75.
117. D'Souza B, Butler T, Shakeshaft A, Calder I, Conigrave K, Doyle M. Learnings from a prison-based drug treatment program on planning for release: A qualitative study. Drug Alcohol Rev. 2024;43(1):245-56.
118. D'Souza RM, Butler T, Petrovsky N. Assessment of cardiovascular disease risk factors and diabetes mellitus in Australian prisons: Is the prisoner population unhealthier than the rest of the Australian population? Aust N Z J Public Health. 2005;29(4):318-23.
119. Daley GM, Russell DB, Tabrizi SN, Twin J, McBride WJH. Mycoplasma genitalium and its resistance to azithromycin in incarcerated men from Far North Queensland. Sex Health. 2014;11(6):587-9.
120. Dalton V. Death and dying in prison in Australia: National overview, 1980-1998. J Law Med Ethics. 1999;27(3):269-10.

121. Darke S, Kaye S. Attempted suicide among injecting and noninjecting cocaine users in Sydney, Australia. J Urban Health. 2004;81(3):505-15.
122. Darke S, Kaye S, Finlay-Jones R. Drug use and injection risk-taking among prison methadone maintenance patients. Addiction (Abingdon, England). 1998;93(8):1169-75.
123. Darke S, Ross J, Zador D, Sunjic S. Heroin-related deaths in New South Wales, Australia, 1992-1996. Drug Alcohol Depend. 2000;60(2):141-50.
124. David MC, Alati R, Ware RS, Kinner SA. Attrition in a longitudinal study with hard-to-reach participants was reduced by ongoing contact. J Clin Epidemiol. 2013;66(5):575-81.
125. Day C, Dolan K. Correlates of hepatitis C testing among heroin injectors in Sydney. Health Promot J Austr. 2006;17(1):70-2.
126. Day C, Ross J, Dolan K. Characteristics of Aboriginal injecting drug users in Sydney, Australia: Prison history, hepatitis C status and drug treatment experiences. J Ethn Subst Abuse. 2003;2(3):51-8.
127. de Andrade DF, Spittal MJ, Snow KJ, Taxman FS, Crilly JL, Kinner SA. Emergency health service contact and reincarceration after release from prison: A prospective cohort study. Crim Behav Ment Health. 2019;29(2):85-93.
128. Dean K, Korobanova D. Brief mental health screening of prison entrants: Psychiatric history versus symptom screening for the prediction of in-prison outcomes. J Forens Psychiatry Psychol. 2018;29(3):455-66.
129. Dean K, Lyons G, Johnson A, McEntyre E. First Nations peoples in the forensic mental health system in New South Wales: Characteristics and rates of criminal charges post-release. Aust N Z J Psychiatry. 2023;57(6):904-13.
130. Dear GE. Non-fatal self-harm in Western Australian prisons: Who, where, when and why. Australian and New Zealand Journal of Criminology. 2001;34(1):47-66.
131. Dear GE. Self-harm in Western Australian prisons: Differences between prisoners who have self-harmed and those who have not. Australian and New Zealand Journal of Criminology. 2001;34(3):277-92.
132. Degenhardt L, Larney S, Gisev N, Trevena J, Burns L, Kimber J, et al. Imprisonment of opioid-dependent people in New South Wales, Australia, 2000-2012: A retrospective linkage study. Aust N Z J Public Health. 2014;38(2):165-70.
133. Degenhardt L, Coffey C, Hearps S, Kinner SA, Borschmann R, Moran P, et al. Associations between psychotic symptoms and substance use in young offenders. Drug Alcohol Rev. 2015;34(6):673-82.
134. Degenhardt L, Larney S, Kimber J, Gisev N, Farrell M, Dobbins T, et al. The impact of opioid substitution therapy on mortality post-release from prison: Retrospective data linkage study. Addiction (Abingdon, England). 2014;109(8):1306-17.
135. Dellar K, Roberts L, Bullen J, Downe K, Kane R. Validation of the YLS/CMI on an Australian juvenile offending population. Int J Offender Ther Comp Criminol. 2023;67(8):861-83.
136. Denton B. Psychiatric morbidity and substance dependence among women prisoners: An Australian study. Psychiatry Psychology and Law. 1995;2(2):173-7.
137. Denton M, Foster M, Bl, Robert. How the prison-to-community transition risk environment influences the experience of men with co-occurring mental health and substance use disorder. Aust N Z J Criminol. 2017;50(1):39.
138. Dias S, Kinner SA, Heffernan E, Waghorn G, Ware R. Identifying rehabilitation priorities among ex-prisoners vulnerable to mental illnesses and substance abuse. J Rehabil. 2018;84(3):46-56.
139. Dias S, Ware RS, Kinner SA, Lennox NG. Physical health outcomes in prisoners with intellectual disability: a cross-sectional study. J Intellect Disabil Res. 2013;57(12):1191-6.
140. Dias S, Ware RS, Kinner SA, Lennox NG. Co-occurring mental disorder and intellectual disability in a large sample of Australian prisoners. Aust N Z J Psychiatry. 2013;47(10):938-44.
141. Dixon A, Howie P, Starling J. Psychopathology in female juvenile offenders. J Child Psychol Psychiatry. 2004;45(6):1150-8.
142. Dolan KA, Shearer J, MacDonald M, Mattick RP, Hall W, Wodak AD. A randomised controlled trial of methadone maintenance treatment versus wait list control in an Australian prison system. Drug Alcohol Depend. 2003;72(1):59-65.
143. Dolan KA, Shearer J, White B, Zhou J, Kaldor J, Wodak AD. Four-year follow-up of imprisoned male heroin users and methadone treatment: mortality, re-incarceration and hepatitis C infection. Addiction (Abingdon, England). 2005;100(6):820-8.
144. Dolan K, Rodas A, Bode A. Drug and alcohol use and treatment for Australian Indigenous and non-Indigenous prisoners: demand reduction strategies. Int J Prison Health. 2015;11(1):30-8.
145. Dolan K, Teutsch S, Scheuer N, Levy M, Rawlinson W, Kaldor J, et al. Incidence and risk for acute hepatitis C infection during imprisonment in Australia. Eur J Epidemiol. 2010;25(2):143-8.

146. Dominguez D JF, Truong J, Burnett J, Satyen L, Akhlaghi H, Stella J, et al. Effects of the response to the COVID-19 pandemic on assault-related head injury in Melbourne: A retrospective study. Int J Environ Res Public Health. 2022;20(1).
147. Doolan I, Najman JM, Cherney A. Health needs of Australian Indigenous young people entering detention. J Paediatr Child Health. 2012;48(10):896-901.
148. Dowell CM, Mejia GC, Preen DB, Segal L. Determinants of infant mortality for children of women prisoners: A longitudinal linked data study. BMC Pregnancy Childbirth. 2018;18(202).
149. Dowell CM, Mejia GC, Preen DB, Segal L. Low birth weight and maternal incarceration in pregnancy: A longitudinal linked data study of Western Australian infants. SSM Popul Health. 2019;7:100324.
150. Doyle MF, Al-Ansari F, Kaye S, Williams M, Conigrave K, Bowman J. Alcohol and other drug use before custody among Aboriginal and non-Aboriginal people in New South Wales, Australia. Aust N Z J Public Health. 2023;47(3):100052.
151. Doyle MF, Butler TG, Shakeshaft A, Guthrie J, Reekie J, Schofield PW. Alcohol and other drug use among Aboriginal and Torres Strait Islander and non-Aboriginal and Torres Strait Islander men entering prison in New South Wales. Health Justice. 2015;3(1):1.
152. Doyle MF, Guthrie J, Butler T, Shakeshaft A, Conigrave K, Williams M. Onset and trajectory of alcohol and other drug use among Aboriginal men entering a prison treatment program: A qualitative study. Drug Alcohol Rev. 2020;39(6):704-12.
153. Draper B. Attempted suicide in older people in New South Wales, Australia, 1870-1908. Hist Psychiatry. 2023:957154X231168956.
154. Drew LR. Alcoholic offenders in a Victorian prison. Med J Aust. 1961;48:575-8.
155. Dunlop AJ, White B, Roberts J, Cretikos M, Attalla D, Ling R, et al. Treatment of opioid dependence with depot buprenorphine (CAM2038) in custodial settings. Addiction. 2022;117(2):382-91.
156. Eckstein G, Levy M, Butler T. Can health inequalities be addressed? An assessment of Prisoner Health Services in New South Wales, Australia. Int J Prison Health. 2007;3(1):69-76.
157. Edwards LM, Chang S, Zeki R, Jamieson SK, Bowman J, Cooper C, et al. The associations between social determinants of health, mental health, substance-use and recidivism: A ten-year retrospective cohort analysis of women who completed the connections programme in Australia. Harm Reduct J. 2024;21(1).
158. Egeressy A, Butler T, Hunter M. 'Traumatisers or traumatised': Trauma experiences and personality characteristics of Australian prisoners. Int J Prison Health. 2009;5(4):212-22.
159. Ellem K. Experiences of leaving prison for people with intellectual disability. J Learn Disabil Offending Behav. 2012;3(3):127.
160. Ellem K, Wilson J, Chui WH. Effective responses to offenders with intellectual disabilities: Generalist and specialist services working together. Aust Soc Work. 2012;65(3):398-412.
161. Eriksson L, Bryant S, McPhedran S, Mazerolle P, Wortley R. Alcohol and drug problems among Australian homicide offenders. Addiction. 2021;116(3):618-31.
162. Eriksson L, McPhedran S, Mazerolle P, Wortley R. Gendered entitlement or generally violent? Sociodemographic, developmental, and gender-based attitudinal characteristics of men who commit homicide. Homicide Stud. 2023;27(3):384-402.
163. Fairley CK, Leslie DE, Nicholson S, Gust ID. Epidemiology and hepatitis C virus in Victoria. Med J Aust. 1990;153(5):271-3.
164. Falster K, Kaldor JM, Maher L. Hepatitis C virus acquisition among injecting drug users: A cohort analysis of a national repeated cross-sectional survey of needle and syringe program attendees in Australia, 1995-2004. J Urban Health. 2009;86(1):106-18.
165. Fasher AM, Dunbar N, Rothenbury BA, Bebb DK, Young SJ. The health of a group of young Australians in a New South Wales juvenile justice detention centre: A pilot study. J Paediatr Child Health. 1997;33(5):426-9.
166. Fazel S, Grann M, Kling B, Hawton K. Prison suicide in 12 countries: An ecological study of 861 suicides during 2003-2007. Soc Psychiatry Psychiatr Epidemiol. 2011;46(3):191-5.
167. Field C. Hazardous alcohol consumption in non-Aboriginal male inmates in New South Wales. Int J Prison Health. 2018;14(1):46-55.
168. Field C, Archer V. Comparing health status, disability, and access to care in older and younger inmates in the New South Wales corrections system. Int J Prison Health. 2019;15(2):153-61.
169. Field C, Zovko A, Bowman J. An examination of chronic ill-health and lifestyle factors among inmates: Searching for the healthy immigrant effect in New South Wales Prisons. Int J Prison Health. 2020;16(2):207-19.
170. Fleming J, Gately N, Kraemer S. Creating HoPE: Mental health in Western Australian maximum security prisons. Psychiatr Psychol Law. 2012;19(1):60-74.

171. Fleming J, Butler T, Donovan B, Levy M, Kaldor J. Childhood sexual abuse among Australian prisoners. Venereology. 2001;14(3):109-15.
172. Forrest G, Boonwaat L, Douglas J, Awofeso N. Enhanced chlamydia surveillance in New South Wales (Australia) prisons, 2005-2007. Int J Prison Health. 2009;5(4):233-40.
173. Forsyth S, Alati R, Kinner SA. Asthma-related mortality after release from prison: A retrospective data linkage study. J Asthma. 2023;60(1):167-73.
174. Forsyth SJ, Alati R, Ober C, Williams GM, Kinner SA. Striking subgroup differences in substance-related mortality after release from prison. Addiction (Abingdon, England). 2014;109(10):1676-83.
175. Forsyth SJ, Carroll M, Lennox N, Kinner SA. Incidence and risk factors for mortality after release from prison in Australia: A prospective cohort study. Addiction. 2018;113(5):937-45.
176. Garner JJ, Gaughwin M, Dodding J, Wilson K. Prevalence of hepatitis C infection in pregnant women in South Australia. Med J Aust. 1997;167(9):470-2.
177. Gates JA, Post JJ, Kaldor JM, Pan Y, Haber PS, Lloyd AR, et al. Risk factors for hepatitis C infection and perception of antibody status among male prison inmates in the hepatitis C incidence and transmission in prisons study cohort, Australia. J Urban Health. 2004;81(3):448-52.
178. Gaughwin MD, Ali R. HIV infection among injecting drug users in the South Australian methadone program. Med J Aust. 1995;162(5):242-4.
179. Gaughwin MD, Douglas RM, Liew C. HIV prevalence and risk behaviours for HIV transmission in South Australian prisons. AIDS. 1991;5(7):845-51.
180. Gibbs D, Colledge-Frisby S, Farnbach S, Doyle M, Shakeshaft A, Larney S. Associations between supported accommodation and health and re-offending outcomes: A retrospective data linkage study. J Urban Health. 2024.
181. Gibbs D, Price O, Grebely J, Larney S, Sutherl, R., et al. Hepatitis C virus cascade of care among people who inject drugs in Australia: Factors associated with testing and treatment in a universal healthcare system. Drug Alcohol Depend. 2021;228.
182. Gidding HF, Mahajan D, Reekie J, Lloyd AR, Dwyer DE, Butler T. Hepatitis B immunity in Australia: A comparison of national and prisoner population serosurveys. Epidemiol Infect. 2015;143(13):2813-21.
183. Gilchrist L, Jamieson SK, Zeki R, Ward S, Chang S, Sullivan E. Understanding health and social service accessibility for young people with problematic substance use exiting prison in Australia. Health Soc Care Community. 2022;30(6):e4735-e44.
184. Gilles M, Swingler E, Craven C, Larson A. Prison health and public health responses at a regional prison in Western Australia. Aust N Z J Public Health. 2008;32(6):549-53.
185. Gisev N, Gibson A, Larney S, Kimber J, Williams M, Clifford A, et al. Offending, custody and opioid substitution therapy treatment utilisation among opioid-dependent people in contact with the criminal justice system: Comparison of indigenous and non-Indigenous Australians. BMC Public Health. 2014;14(920).
186. Glaser WF. Admissions to a prison psychiatric unit. Aust N Z J Psychiatry. 1985;19(1):45-52.
187. Glaser W, Deane K. Normalisation in an abnormal world: A study of prisoners with an intellectual disability. Int J Offender Ther Comp Criminol. 1999;43(3):338-56.
188. Glaser W, Laster K. Are the Mentally Ill being Criminalised?: Admission of Prisoners to Psychiatric Hospitals before and after the 1986 Mental Health Act (Vic). Aust N Z J Criminol. 1990;23(4):230.
189. Gordon D. Sickness and death at the moreton bay convict settlement. Med J Aust. 1963;17:473-80.
190. Goulter N, Kimonis ER, Heller E. Antisocial process screening device subscales predict recidivism in an Australian juvenile offender sample. J Psychopathol Behav Assess. 2018;40(2):159-68.
191. Gower M, Morgan F, Saunders J. Aboriginality and violence: Gender and cultural differences on the Level of Service/Risk, Need, Responsivity (LS/RNR) and Violence Risk Scale (VRS). Psychiatr Psychol Law. 2023;30(3):249-70.
192. Gower M, Spiranovic C, Morgan F, Saunders J. The criminogenic profile of violent female offenders incarcerated in Western Australian prisons as per the Level of Service/Risk, Need, Responsivity (LS/RNR) and Violence Risk Scale (VRS). Psychiatr Psychol Law. 2023;30(2):192-210.
193. Graffam J, Shinkfield AJ. The life conditions of Australian ex-prisoners: An analysis of intrapersonal, subsistence, and support conditions. Int J Offender Ther Comp Criminol. 2012;56(6):897-916.
194. Graham A. Post-prison mortality: Unnatural death among people released from victorian prisons between January 1990 and December 1999. Australian and New Zealand Journal of Criminology. 2003;36(1):94-108.
195. Green B, Denton M, Heffernan E, Russell B, Stapleton L, Waterson E. From custody to community: Outcomes of community-based support for mentally ill prisoners. Psychiatr Psychol Law. 2016;23(5):798-808.

196. Gullotta M, Greenberg D, Adily A, Albalawi O, Karminia A, Knight L, et al. Physical health status of individuals convicted of sexual offences: Results from an Australian prisoner cohort. J Forensic Leg Med. 2023;100.
197. Gullotta M, Greenberg D, Adily A, Cale J, Butler TG. Implications of sex offender classification on reporting demographic characteristics, health, and criminal careers: Results from an Australian jurisdiction. BMC Med Res Methodol. 2020;20(1):97.
198. Gullotta M, Greenberg D, Albalawi O, Adily A, Karminia A, Knight L, et al. Self-harm and suicidality among three subgroups of male sex offenders: Results from an Australian prisoner cohort. Health Justice. 2021;9(19).
199. Guthrie JA, Lokuge KM, Levy MH. Influenza control can be achieved in a custodial setting: Pandemic (H1N1) 2009 and 2011 in an Australian prison. Public Health. 2012;126(12):1032-7.
200. Haber PS, Parsons SJ, Harper SE, White PA, Rawlinson WD, Lloyd AR. Transmission of hepatitis C within Australian prisons. Med J Aust. 1999;171(1):31-3.
201. Hail-Jares K, Cumming C, Young JT, Borschmann R, Lennox N, Kinner SA. Self-harm and suicide attempts among incarcerated lesbian, gay and bisexual people in Australia. Aust N Z J Psychiatry. 2023;57(4):562-71.
202. Hajarizadeh B, Carson JM, Byrne M, Grebely J, Cunningham E, Amin J, et al. Incidence of Hepatitis C virus infection in the prison setting: The SToP-C study. J Viral Hepat. 2023;31(1):21-34.
203. Hajarizadeh B, Grebely J, Byrne M, Marks P, Amin J, McManus H, et al. Evaluation of hepatitis C treatment-as-prevention within Australian prisons (SToP-C): A prospective cohort study. Lancet Gastroenterol Hepatol. 2021;6(7):533-46.
204. Halder A, Li V, Sebastian M, Nazareth S, Tuma R, Cheng W, et al. Use of telehealth to increase treatment access for prisoners with chronic hepatitis C. Intern Med J. 2021;51(8):1344-7.
205. Hamilton S, Reibel T, Maslen S, Watkins R, Jacinta F, Passmore H, et al. Disability “in-justice”: The benefits and challenges of “yarning” with young people undergoing diagnostic assessment for fetal alcohol spectrum disorder in a youth detention center. Qual Health Res. 2020;30(2):314-27.
206. Hampton S, Weston KM, McCarthy LR, Mackinnon T. Pandemics in New South Wales prisons: The more things change. Commun Dis Intell. 2021;45.
207. Hando J, Howard J, Zibert E. Risky drug practices and treatment needs of youth detained in New South Wales Juvenile Justice Centres. Drug Alcohol Rev. 1997;16(2):137-45.
208. Hannan-Jones M, Capra S. Prevalence of diet-related risk factors for chronic disease in male prisoners in a high secure prison. Eur J Clin Nutr. 2016;70(2):212-6.
209. Harman K, Maxwell-Stewart H. Aboriginal Deaths in Custody in Colonial Australia, 1805-1860. J Colon Colon Hist. 2012;13(2):N_A.
210. Haysom L, Canessa J, Kasinathan J, Blomgren D. Neutropenia in incarcerated adolescents secondary to intranasal quetiapine misuse. J Child Adolesc Psychopharmacol. 2020;30(10):617-9.
211. Haysom L, Cross M, Anastasas R, Hampton S, Harris M, Sneddon K. Methicillin-resistant Staphylococcus aureus skin and soft tissue infections in young people in custody in New South Wales. J Paediatr Child Health. 2019;55(2):224-8.
212. Haysom L, Indig D, Byun R, Moore E, Van Den Dolder P. Oral health and risk factors for dental disease of Australian young people in custody. J Paediatr Child Health. 2015;51(5):545-51.
213. Haysom L, Indig D, Moore E, Gaskin C. Intellectual disability in young people in custody in New South Wales, Australia - prevalence and markers. J Intellect Disabil Res. 2014;58(11):1004-14.
214. Haysom L, Indig D, Moore E, Hardy LL, Dolder PAvd. Prevalence and perceptions of overweight and obesity in Aboriginal and non-Aboriginal young people in custody. Med J Aust. 2013;199(4):266-70.
215. Haysom L, Lawrence D, Mellish D, Burns P, Khale P, Arulampalam A, et al. Use of nicotine replacement therapy in young people entering custody in New South Wales, Australia. J Paediatr Child Health. 2017;53(7):675-9.
216. Heffernan EB, Andersen KC, Dev A, Kinner S. Prevalence of mental illness among Aboriginal and Torres Strait Islander people in Queensland prisons. Aus Indig Health Bull. 2012;12(4):unpaginated.
217. Heffernan E, Davidson F, Andersen K, Kinner S. Substance use disorders among Aboriginal and Torres Strait Islander people in custody: A public health opportunity. Health Justice. 2016;4(12).
218. Heffernan E, Andersen K, Davidson F, Kinner SA. PTSD among Aboriginal and Torres Strait Islander people in custody in Australia: Prevalence and correlates. J Trauma Stress. 2015;28(6):523-30.
219. Hellard ME, Hocking JS, Crofts N. The prevalence and the risk behaviours associated with the transmission of hepatitis C virus in Australian correctional facilities. Epidemiol Infect. 2004;132(3):409-15.
220. Henderson CAF, Bull M. Sentencing and the over-representation of people with cognitive disability in the Australian criminal justice system. Curr Issues Crim Justice. 2023;36(1):81-98.

221. Herrman H, McGorry P, Mills J, Singh B. Hidden severe psychiatric morbidity in sentenced prisoners: An Australian study. Am J Psychiatry. 1991;148(2):236-9.
222. Herrman H, Mills J, Doidge G, McGorry P, Singh B. The use of psychiatric services before imprisonment: A survey and case register linkage of sentenced prisoners in Melbourne. Psychol Med. 1994;24(1):63-8.
223. Hesse S, Williamson K, Bonney D, Finley M, Meehan T. Cancer screening in prisons: Lessons for health providers. Aust J Prim Health. 2023;29(1):16-9.
224. Hilder L, Walker JR, Levy MH, Sullivan EA. Preparing linked population data for research: Cohort study of prisoner perinatal health outcomes. BMC Med Res Methodol. 2016;16:72.
225. Hill PL, Stoové M, Agius PA, Maher L, Hickman M, Crawford S, et al. Mortality in the SuperMIX cohort of people who inject drugs in Melbourne, Australia: A prospective observational study. Addiction. 2022;117(12):3091-8.
226. Hobbs M, Krazlan K, Ridout S, Mai B, Knuiman M, Chapman R. Mortality and morbidity in prisoners after release from prison in Western Australia 1995-2003. Trends Issues Crim Crim Justice. 2006;320:1-6.
227. Hobday S, Valerio H, Combo T, Monaghan R, Clarke S, Silk D, et al. Evaluating the prevalence of current hepatitis C infection and treatment among Aboriginal and Torres Strait Islander peoples who inject drugs in Australia: The ETHOS engage study. Drug Alcohol Rev. 2023;42(7):1617-32.
228. Holland S, Persson P. Intellectual disability in the Victorian prison system: Characteristics of prisoners with an intellectual disability released from prison in 2003-2006. Psychol Crime Law. 2011;17(1):25.
229. Holmwood C, Marriott M, Humeniuk R. Substance use patterns in newly admitted male and female South Australian prisoners using the WHO-ASSIST (Alcohol, Smoking and Substance Involvement Screening Test). Int J Prison Health. 2008;4(4):198-207.
230. Howard J, Lennings CJ, Copel, J. Suicidal behavior in a young offender population. Crisis. 2003;24(3):98-104.
231. Howard MVA, Corben SP, Raudino A, Galouzis JJ. Maintaining safety in the prison environment: A multilevel analysis of inmate victimisation in assaults. Int J Offender Ther Comp Criminol. 2020;64(10):1091-113.
232. Howard J, Zibert E. Curious, bored and wanting to feel good: The drug use of detained young offenders. Drug Alcohol Rev. 1990;9(3):225-31.
233. Hurley W. Suicides by prisoners. Med J Aust. 1989;151(4):188-90.
234. Hurley W, Dunne MP. Psychological distress and psychiatric morbidity in women prisoners. Aust N Z J Psychiatry. 1991;25(4):461-70.
235. Indermaur D, Upton K. Alcohol and drug use patterns of prisoners in Perth. Aust N Z J Criminol. 1988;21(3):144-67.
236. Indig D, Haysom L. Smoking behaviours among young people in custody in New South Wales, Australia. Drug Alcohol Rev. 2012;31(5):631-7.
237. Indig D, Frewen A, Moore E. Predictors and correlates of re-incarceration among Australian young people in custody. Aust N Z J Criminol. 2016;49(1):73.
238. Islam MM, Topp L, Iversen J, Day C, Conigrave KM, Maher L. Healthcare utilisation and disclosure of injecting drug use among clients of Australia's needle and syringe programs. Aust N Z J Public Health. 2013;37(2):148-54.
239. Jama-Alol KA, Malacova E, Ferrante A, Alan J, Stewart L, Preen D. Influence of offence type and prior imprisonment on risk of death following release from prison: A whole-population linked data study. Int J Prison Health. 2015;11(2):108-18.
240. Janca E, Keen C, Willoughby M, Young JT, Kinner SA. Sex differences in acute health service contact after release from prison in Australia: A data linkage study. Public Health. 2023;223:240-8.
241. Jin X, Kinner SA, Hopkins R, Stockings E, Courtney RJ, Shakeshaft A, et al. A randomised controlled trial of motivational interview for relapse prevention after release from smoke-free prisons in Australia. Int J Prison Health. 2021;17(4):462-76.
242. Johnson H. Drug use by incarcerated women offenders. Drug Alcohol Rev. 2006;25(5):433-7.
243. Jones IH, Marris B, Hornsby H. Psychiatric characteristics of female prisoners in Tasmania. Aust N Z J Psychiatry. 1995;29(4):671-7.
244. Jones NR, Hickman M, Nielsen S, Larney S, Dobbins T, Ali R, et al. The impact of opioid agonist treatment on fatal and non-fatal drug overdose among people with a history of opioid dependence in NSW, Australia, 2001-2018: Findings from the OATS retrospective linkage study. Drug Alcohol Depend. 2022;236:109464.
245. Kariminia A, Butler TG, Corben SP, Levy MH, Grant L, Kaldor JM, et al. Extreme cause-specific mortality in a cohort of adult prisoners - 1988 to 2002: A data-linkage study. Int J Epidemiol. 2007;36(2):310-6.

246. Kariminia A, Butler T, Levy M. Aboriginal and non-Aboriginal health differentials in Australian prisoners. Aust N Z J Public Health. 2007;31(4):366-71.
247. Kariminia A, Butler T, Corben S, Kaldor J, Levy M, Law M. Mortality among prisoners: How accurate is the Australian National Death Index? Aust N Z J Public Health. 2005;29(6):572-5.
248. Kariminia A, Butler T, Jones J, Law M. Increased mortality among Indigenous persons during and after release from prison in New South Wales. Aust N Z J Public Health. 2012;36(3):274-80.
249. Kariminia A, Law MG, Butler TG, Corben SP, Levy MH, Kaldor JM, et al. Factors associated with mortality in a cohort of Australian prisoners. Eur J Epidemiol. 2007;22(7):417-28.
250. Kariminia A, Law MG, Butler TG, Levy MH, Corben SP, Kaldor JM, et al. Suicide risk among recently released prisoners in New South Wales, Australia. Med J Aust. 2007;187(7):387-90.
251. Kasinathan J. Predictors of rapid reincarceration in mentally ill young offenders. Australas Psychiatry. 2015;23(5):550-5.
252. Kasinathan J, Haysom L, Andriotis H, Wheaton M, Lloyd T, Langstaff R, et al. Keeping COVID out: A collaborative approach to COVID-19 is associated with a significant reduction in self-harm in young people in custody. Australas Psychiatry. 2021;29(4):412-6.
253. Kavanagh L, Rowe D, Hersch J, Barnett KJ, Reznik R. Neurocognitive deficits and psychiatric disorders in a NSW prison population. Int J Law Psychiatry. 2010;33(1):20-6.
254. Kaye S, Lewandowski A, Bowman J, Doyle MF. Crystal methamphetamine use among young people entering custody: Prevalence, correlates and comorbidity. Drug Alcohol Rev. 2020;40(7):1266-74.
255. Keen C, Kinner SA, Borschmann R, Young JT. Comparing the predictive capability of self-report and medically-verified non-fatal overdose in adults released from prison: A prospective data linkage study. Drug Alcohol Depend. 2020;206:107742.
256. Keen C, Young JT, Borschmann R, Kinner SA. Non-fatal drug overdose after release from prison: A prospective data linkage study. Drug Alcohol Depend. 2020;206:107707.
257. Kelly ML, Riordan SM, Bopage R, Lloyd AR, Post JJ. Capacity of non-invasive hepatic fibrosis algorithms to replace transient elastography to exclude cirrhosis in people with hepatitis C virus infection: A multi-centre observational study. PLoS One. 2018;13(2):e0192763.
258. Kendall S, Lighton S, Sherwood J, Baldry E, Sullivan E. Holistic conceptualizations of health by incarcerated Aboriginal women in New South Wales, Australia. Qual Health Res. 2019;29(11):1549-65.
259. Kendall S, Lighton S, Sherwood J, Baldry E, Sullivan EA. Incarcerated Aboriginal women's experiences of accessing healthcare and the limitations of the 'equal treatment' principle. Int J Equity Health. 2020;19(1):Article 48.
260. Kenny DT, Grant J. Reliability of self-report of health in juvenile offenders. Vulnerable Child Youth Stud. 2007;2(2):127-41.
261. Kenny DT, Lennings CJ. Cultural group differences in social disadvantage, offence characteristics, and experience of childhood trauma and psychopathology in incarcerated juvenile offenders in NSW, Australia: Implications for service delivery. Psychiatr Psychol Law. 2007;14(2):294-305.
262. Kenny DT, Lennings CJ, Munn OA. Risk factors for self-harm and suicide in incarcerated young offenders: Implications for policy and practice. J Forensic Psychol Pract. 2008;8(4):358-82.
263. Kerley K, Cunneen C. Deaths in custody in Australia: The untold story of aboriginal and Torres Strait Islander women. Can J Women Law. 1995;8(2):531.
264. Kerry J, Tan GKY, Panton KR, Mutch R, Freeman J, Passmore H, et al. Neuropsychological profiles of adolescents sentenced to detention in Western Australia with and without prenatal alcohol exposure. Crim Behav Ment Health. 2024.
265. Kerslake M, Simpson M, Richmond R, Albany H, Butler T. Risky alcohol consumption prior to incarceration: A cross-sectional study of drinking patterns among Australian prison entrants. Drug Alcohol Rev. 2020;39(6):694-703.
266. Kim SB, White B, Roberts J, Day CA. Substance use among pregnant women in NSW prisons. Int J Drug Policy. 2023;122:Article 104256
267. Kinner S. The post-release experience of prisoners in Queensland. Trends Issues Crim Crim Justice. 2006;325:1-6.
268. Kinner SA. Continuity of health impairment and substance misuse among adult prisoners in Queensland, Australia. Int J Prison Health. 2006;2(2):101-13.
269. Kinner SA, Forsyth SJ. Development and validation of a national system for routine monitoring of mortality in people recently released from prison. PLoS One. 2016;11(6):e0157328.
270. Kinner SA, Alati R, Longo M, Spittal MJ, Boyle FM, Williams GM, et al. Low-intensity case management increases contact with primary care in recently released prisoners: A single-blinded, multisite, randomised controlled trial. J Epidemiol Community Health. 2016;70(7):683-8.

271. Kinner SA, Degenhardt L, Coffey C, Sawyer S, Hearps S, Patton G. Complex health needs in the youth justice system: A survey of community-based and custodial offenders. J Adolesc Health. 2014;54(5):521-6.
272. Kinner SA, Dietze PM, Gouillou M, Alati R. Prevalence and correlates of alcohol dependence in adult prisoners vary according to Indigenous status. Aus Indig Health Bull. 2012;12(4):unpaginated.
273. Kinner SA, Jenkinson R, Gouillou M, Milloy MJ. High-risk drug-use practices among a large sample of Australian prisoners. Drug Alcohol Depend. 2012;126(1):156-60.
274. Kinner SA, Lennox N, Williams GM, Carroll M, Quinn B, Boyle FM, et al. Randomised controlled trial of a service brokerage intervention for ex-prisoners in Australia. Contemp Clin Trials. 2013;36(1):198-206.
275. Kinner SA, Preen DB, Kariminia A, Butler T, Andrews JY, Stoove M, et al. Counting the cost: Estimating the number of deaths among recently released prisoners in Australia. Med J Aust. 2011;195(2):64-8.
276. Kippin NR, Leitao S, Finlay-Jones A, Baker J, Watkins R. The oral and written narrative language skills of adolescent students in youth detention and the impact of language disorder. J Commun Disord. 2021;90:N.PAG-N.PAG.
277. Kippin NR, Leitao S, Watkins R, Finlay-Jones A, Condon C, Marriott R, et al. Language diversity, language disorder, and fetal alcohol spectrum disorder among youth sentenced to detention in Western Australia. Int J Law Psychiatry. 2018;61:40-9.
278. Kirwan A, Curtis M, Dietze P, Aitken C, Woods E, Walker S, et al. The prison and transition health (PATH) cohort study: Study protocol and baseline characteristics of a cohort of men with a history of injecting drug use leaving prison in Australia. J Urban Health. 2019;96(3):400-10.
279. Kirwan A, Quinn B, Winter R, Kinner SA, Dietze P, Stoove M. Correlates of property crime in a cohort of recently released prisoners with a history of injecting drug use. Harm Reduct J. 2015;12:23.
280. Koller KM, Castanos JN. Parental deprivation and attempted suicide in prison populations. Med J Aust. 1969;1(17):858-61.
281. Korobanova D, Spencer S-J, Dean K. Prevalence of mental health problems in men and women in an Australian prison sample: Comparing psychiatric history taking and symptom screening approaches. Int J Forensic Ment Health. 2022;21(1):89-105.
282. Kosky RJ, Sawyer MG, Fotheringham M. The mental health status of adolescents released from custody: A preliminary study. Aust N Z J Psychiatry. 1996;30(3):326-31.
283. Kosky RJ, Sawyer MG, Gowl, C. J. Adolescents in custody: Hidden psychological morbidity? Med J Aust. 1990;153(1):24-7.
284. Lafferty L, Rance J, Grebely J, Dore GJ, Lloyd AR, Treloar C. Perceptions and concerns of hepatitis C reinfection following prison-wide treatment scale-up: Counterpublic health amid hepatitis C treatment as prevention efforts in the prison setting. Int J Drug Policy. 2020;77.
285. Lafferty L, Schroeder S, Marshall AD, Drysdale K, Higgs P, Stoove M, et al. Trust and service engagement among people who inject drugs after release from prison. Int J Drug Policy. 2023;111:103925.
286. Lafferty L, Sheehan Y, Cochrane A, Grebely J, Lloyd AR, Treloar C. Reducing barriers to the hepatitis C care cascade in prison via point-of-care RNA testing: A qualitative exploration of men in prison using an integrated framework. Addiction. 2023;118(6):1153-60.
287. Larney S, Gisev N, Farrell M, Dobbins T, Burns L, Gibson A, et al. Opioid substitution therapy as a strategy to reduce deaths in prison: Retrospective cohort study. BMJ Open. 2014;4(4):e004666.
288. Larney S, Jones NR, Hickman M, Nielsen S, Ali R, Degenhardt L. Does opioid agonist treatment reduce overdose mortality risk in people who are older or have physical comorbidities? Cohort study using linked administrative health data in New South Wales, Australia, 2002–17. Addiction. 2023;118(8):1527-39.
289. Larney S, Lai W, Dolan K, Zador D. Monitoring a prison opioid treatment program over a period of change to clinical governance arrangements, 2007-2013. J Subst Abuse Treat. 2016;70:58-63.
290. Larney S, Topp L, Indig D, O'Driscoll C, Greenberg D. A cross-sectional survey of prevalence and correlates of suicidal ideation and suicide attempts among prisoners in New South Wales, Australia. BMC Public Health. 2012;12(14).
291. Larney S, Toson B, Burns L, Dolan K. Effect of prison-based opioid substitution treatment and post-release retention in treatment on risk of re-incarceration. Addiction (Abingdon, England). 2011;107(2):372-80.
292. Lawlor D, Kosky R. Serious suicide attempts among adolescents in custody. Aust N Z J Psychiatry. 1992;26(3):474-8.

293. Le Gautier R, Panozzo S, Bryan T, Lethborg C, Philip J. A thematic analysis of hospital medical records of patients with advanced illness experiencing incarceration in the last 3 months of life. Palliat Med. 2023;37(4):638-45.
294. Leckning B, Condon JR, Das SK, He V, Hirvonen T, Guthridge S. Mental health-related hospitalisations associated with patterns of child protection and youth justice involvement during adolescence: A retrospective cohort study using linked administrative data from the Northern Territory of Australia. Child Youth Serv Rev. 2023;145:N.PAG-N.PAG.
295. Lennings C, Pritchard M. Prevalence of drug use prior to detention among residents of youth detention centres in Queensland. Drug Alcohol Rev. 1999;18(2):145-52.
296. Levy MH, Butler TG, Zhou J. Prevalence of Mantoux positivity and annual risk of infection for tuberculosis in New South Wales prisoners, 1996 and 2001. N S W Public Bull. 2007;18(7):119-24.
297. Levy MH, Quilty S, Young LC, Hunt W, Matthews R, Robertson PW. Pox in the docks: Varicella outbreak in an Australian prison system. Public Health. 2003;117(6):446-51.
298. Levy M, Johnson CG, Kraa E. Tonsillopharyngitis caused by foodborne group A streptococcus: A prison-based outbreak. Clin Infect Dis. 2003;36(2):175-82.
299. Lewis K, Hayes S. Health of women ex-prisoners. Psychiatry Psychology and Law. 1997;4(1):55-64.
300. Li H, Cameron B, Douglas D, Stapleton S, Cheguelman G, Butler T, et al. Incident hepatitis B virus infection and immunisation uptake in Australian prison inmates. Vaccine. 2020;38(16):3255-60.
301. Liang Y, Roberts J, Conigrave K, Kim S, Doyle MF. Alcohol relapse prevention health care after alcohol withdrawal in New South Wales prisons, Australia: A patient file review. Drug Alcohol Rev. 2023;42(7):1733-43.
302. Liao P, Trollor J, Reppermund S, Cvejic RC, Srasuebkul P, Vajdic CM. Factors associated with acute care service use after epilepsy hospitalisation in people with intellectual disability. J Intellect Disabil Res. 2023;67(12):1317-35.
303. Lloyd AR, Clegg J, Lange J, Stevenson A, Post JJ, Lloyd D, et al. Safety and effectiveness of a nurse-led outreach program for assessment and treatment of chronic hepatitis C in the custodial setting. Clin Infect Dis. 2013;56(8):1078-84.
304. Lloyd JE, McEntyre E, Baldry E, Trofimovos J, Indig D, Abbott P, et al. Aboriginal and non-aboriginal Australian former prisoners' patterns of morbidity and risk of hospitalisation. Int J Equity Health. 2017;16:1-8.
305. Love AD, Kinner SA, Young JT. Social environment and hospitalisation after release from prison: A prospective cohort study. Int J Environ Res Public Health. 2017;14(11):Article 1406.
306. Luciani F, Bretana NA, Teutsch S, Amin J, Topp L, Dore GJ, et al. A prospective study of hepatitis C incidence in Australian prisoners. Addiction. 2014;109(10):1695-706.
307. Lynch C, Matthews R, Rosina R. Health as a mediator of change in the trajectory of young people in contact with the criminal justice system. Int J Adolesc Med Health. 2007;19(3):269-76.
308. Macdonald J, Scholes T, Powell K. Listening to Australian Indigenous men: Stories of incarceration and hope. Prim Health Care Res Dev. 2016;17(6):568-77.
309. Macdonald C, Weatherburn D, Butler T, Albalawi O, Greenberg D, Farrell M. Who gets diverted into treatment? A study of defendants with psychosis. Psychiatr Psychol Law. 2024.
310. MacIntyre CR, Carnie J, all M. Risk of transmission of tuberculosis among inmates of an Australian prison. Epidemiol Infect. 1999;123(3):445-50.
311. MacLachlan JH, Romero N, Higgins N, Coutts R, Chan R, Stephens N, et al. Epidemiology of chronic hepatitis B and C in Victoria, Australia: Insights and impacts from enhanced surveillance. Aust N Z J Public Health. 2020;44(1):59-64.
312. Malacova E, Butler T, Richters J, Yap L, Grant L, Richards A, et al. Knowledge of sexually transmissible infections: A comparison of prisoners and the general population. Int J STD AIDS. 2011;22(7):381-6.
313. Malacova E, Butler T, Yap L, Grant L, Richards A, Smith AMA, et al. Sexual coercion prior to imprisonment: Prevalence, demographic and behavioural correlates. Int J STD AIDS. 2012;23(8):533-9.
314. Malvaso C, Day A, Cale J, Hackett L, Delfabbro P, Ross S. Adverse childhood experiences and trauma among young people in the youth justice system. Trends Issues Crim Crim Justice. 2022(651):1-19.
315. Malvaso C, Day A, Casey S, Corrado R. Young offenders, maltreatment, and trauma: A pilot study. Psychiatr Psychol Law. 2017;24(3):458-69.
316. Marr C, Gaskin C, Kasinathan J, Kaye S, Singh Y, Dean K. The prevalence of mental illness in young people in custody over time: A comparison of three surveys in New South Wales. Psychiatr Psychol Law. 2023.

317. Marshall AD, Schroeder SE, Lafferty L, Drysdale K, Baldry E, Stoove M, et al. Perceived access to opioid agonist treatment in prison among people with a history of injection drug use: A qualitative study. J Subst Use Addict Treat. 2023;150:209066.
318. McDonald AM, Ryan JW, Brown PR, Manners CJ, Falconer AD, Kinnear RC, et al. HIV prevalence at reception into Australian prisons, 1991-1997. Med J Aust. 1999;171(1):18-21.
319. McDonald D, Thomson NJ. Australian deaths in custody, 1980-1989. 2. Causes. Med J Aust. 1993;159(9):581-5.
320. McGillivray JA, Gaskin CJ, Newton DC, Richardson BA. Substance use, offending, and participation in alcohol and drug treatment programmes: A comparison of prisoners with and without intellectual disabilities. J Appl Res Intellect Disabil. 2016;29(3):289-94.
321. McGregor C, Ali R, Lokan R, Christie P, Darke S. Accidental fatalities among heroin users in South Australia, 1994-1997: Toxicological findings and circumstances of death. Addict Res Theory. 2002;10(4):335-46.
322. Merone L, Ashton S, Harris A, Edwards WS, Preston-Thomas A, Gair R, et al. A complex increase in hepatitis C virus in a correctional facility: Bumps in the road. Aust N Z J Public Health. 2022;46(3):377-81.
323. Miller ER, Bi P, Ryan P. The prevalence of HCV antibody in South Australian prisoners. J Infect. 2006;53(2):125-30.
324. Miller ER, Bi P, Ryan P. Hepatitis C virus infection in South Australian prisoners: Seroprevalence, seroconversion, and risk factors. Int J Infect Dis. 2009;13(2):201-8.
325. Miller ER, Hellard ME, Bowden S, Bharadwaj M, Aitken CK. Markers and risk factors for HCV, HBV and HIV in a network of injecting drug users in Melbourne, Australia. J Infect. 2009;58(5):375-82.
326. Mills KL, Teesson M, Ross J, Darke S. Predictors of trauma and PTSD among heroin users: A prospective longitudinal investigation. J Drug Issues. 2008;38(2):585.
327. Miner M, Gorta A. Heroin use in the lives of women prisoners in Australia. Aust N Z J Criminol. 1987;20(1):3.
328. Moore E, Gaskin C, Indig D. Childhood maltreatment and post-traumatic stress disorder among incarcerated young offenders. Child Abuse Negl. 2013;37(10):861-70.
329. Moore E, Gaskin C, Indig D. Attempted suicide, self-harm, and psychological disorder among young offenders in custody. J Correct Health Care. 2015;21(3):243-54.
330. Moore E, Indig D, Haysom L. Traumatic brain injury, mental health, substance use, and offending among incarcerated young people. J Head Trauma Rehabil. 2014;29(3):239-47.
331. Moore E, Sunjic S, Kaye S, Archer V, Indig D. Adult ADHD among NSW Prisoners: Prevalence and psychiatric comorbidity. J Atten Disord. 2016;20(11):958-67.
332. Moore E, Winter R, Indig D, Greenberg D, Kinner SA. Non-fatal overdose among adult prisoners with a history of injecting drug use in two Australian states. Drug Alcohol Depend. 2013;133(1):45-51.
333. Morrison S. Custodial suicide in Australia: A comparative study of different populations. Med Sci Law. 1996;36(2):167-77.
334. Moyo N, Tay EL, Denholm J. 'Know your epidemic': Are prisons a potential barrier to TB elimination in an Australian context? Trop Med Infect Dis. 2018;3(3).
335. Murray N, LePage E, Butler T. Hearing health of New South Wales prison inmates. Aust N Z J Public Health. 2004;28(6):537-41.
336. Newnham GM, Munton K, Shaw M, McLachlan SA. Cancer in Victorian prisoners: A description of cancer diagnoses, demographics, risk factors and barriers to optimal care. Intern Med J. 2024;54(2):295-300.
337. Nielssen OB, Stone W, Jones NM, Challis S, Nielssen A, Elliott G, et al. Characteristics of people attending psychiatric clinics in inner Sydney homeless hostels. Med J Aust. 2018;208(4):169-73.
338. Nielssen O, Misrachi S. Prevalence of psychoses on reception to male prisons in New South Wales. Aust N Z J Psychiatry. 2005;39(6):453-9.
339. Nielssen O, Yee NYL, Dean K, Large M. Outcome of serious violent offenders with psychotic illness and cognitive disorder dealt with by the New South Wales criminal justice system. Aust N Z J Psychiatry. 2019;53(5):441-6.
340. O'Driscoll C, Larney S, Indig D, Basson J. The impact of personality disorders, Substance use and other mental illness on re-offending. J Forens Psychiatry Psychol. 2012;23(3):382-91.
341. O'Driscoll CN, Samuels A, Zacka M. Suicide in New South Wales Prisons, 1995-2005: Towards a better understanding. Australian and New Zealand Journal of Psychiatry. 2007;41(6):519-24.
342. O'Sullivan BG, Levy MH, Dolan KA, Post JJ, Barton SG, Dwyer DE, et al. Hepatitis C transmission and HIV post-exposure prophylaxis after needle- and syringe-sharing in Australian prisons. Med J Aust. 2003;178(11):546-9.

343. Ober C, Dingle K, Clavarino A, Najman JM, Alati R, Heffernan EB. Validating a screening tool for mental health and substance use risk in an indigenous prison population. Drug Alcohol Rev. 2013;32(6):611-7.
344. Ogilvie EL, Veit F, Crofts N, Thompson SC. Hepatitis infection among adolescents resident in Melbourne Juvenile Justice Centre: Risk factors and challenges. J Adolesc Health. 1999;25(1):46-51.
345. Ogloff JRP, Pfeifer JE, Shepherd SM, Ciorciari J. Assessing the mental health, substance abuse, cognitive functioning, and social/emotional well-being needs of Aboriginal prisoners in Australia. J Correct Health Care. 2017;23(4):398-411.
346. Osborn M, Butler T, Barnard PD. Oral health status of prison inmates--New South Wales, Australia. Aust Dent J. 2003;48(1):34-8.
347. Overton K, Clegg J, Pekin F, Wood J, McGrath C, Lloyd A, et al. Outcomes of a nurse-led model of care for hepatitis C assessment and treatment with direct-acting antivirals in the custodial setting. Int J Drug Policy. 2019;72:123-8.
348. Panozzo S, Bryan T, Marco D, Collins A, Lethborg C, Philip J. End of life in hospitalised prisoners: A group comparison of palliative medicine and hospital use. BMJ Support Palliat Care. 2021.
349. Papalia N, Baidawi S, Luebbers S, Shepherd S, Ogloff JRP. Patterns of maltreatment co-occurrence in incarcerated youth in Australia. J Interpers Violence. 2022;37(7):NP4341-NP71.
350. Papalia N, Simmons M, Ruffles J, Spivak B, Dunne A, Fullam R, et al. Discriminative and predictive validity of risk assessment measures for women incarcerated for serious violent offences in Australia. Psychiatr Psychol Law. 2024.
351. Papaluca T, Craigie A, McDonald L, Edwards A, Winter R, Hoang A, et al. Care navigation increases initiation of hepatitis C Treatment after release from prison in a prospective randomized controlled trial: The C-LINK study. Open Forum Infect Dis. 2022;9(8).
352. Papaluca T, McDonald L, Craigie A, Gibson A, Desmond P, Wong D, et al. Outcomes of treatment for hepatitis C in prisoners using a nurse-led, statewide model of care. J Hepatol. 2019;70(5):839-46.
353. Perkes I, Schofield PW, Butler T, Hollis SJ. Traumatic brain injury rates and sequelae: A comparison of prisoners with a matched community sample in Australia. Brain Inj. 2011;25(2):131-41.
354. Petschel K, Gall JA. A profile of deaths in custody in Victoria, 1991-96. J Clin Forensic Med. 2000;7(2):82-7.
355. Pham ST, Bull RA, Bennett JM, Rawlinson WD, Dore GJ, Lloyd AR, et al. Frequent multiple hepatitis C virus infections among injection drug users in a prison setting. Hepatology. 2010;52(5):1564-72.
356. Pounder DJ. Death behind bars: An 11-year survey of prisoner deaths in South Australia. Med Sci Law. 1986;26(3):207-13.
357. Puljević C, Coomber R, de Andrade D, Kinner SA. Barriers and facilitators of maintained smoking abstinence following release from smoke-free prisons: A qualitative enquiry. Int J Drug Policy. 2019;68:9-17.
358. Puljević C, de Andrade D, Carroll M, Spittal MJ, Kinner SA. Use of prescribed smoking cessation pharmacotherapy following release from prison: A prospective data linkage study. Tob Control. 2018;27(4):474-8.
359. Puljević C, de Andrade D, Coomber R, Kinner SA. Relapse to smoking following release from smoke-free correctional facilities in Queensland, Australia. Drug Alcohol Depend. 2018;187:127-33.
360. Putniņš AL. Recent drug use and suicidal behaviour among young offenders. Drug Alcohol Rev. 1995;14(2):151-8.
361. Putniņš AL. The Adolescent Alcohol Involvement Scale: Some findings with young offenders. Drug Alcohol Rev. 1992;11(3):253-8.
362. Putniņš AL. Correlates and predictors of self-reported suicide attempts among incarcerated youths. Int J Offender Ther Comp Criminol. 2005;49(2):143-57.
363. Putniņš AL, Harvey SJ. Alcohol abuse among young offenders. Med J Aust. 1992;156(11):753-5.
364. Quinn S, Rance G. The extent of hearing impairment amongst Australian Indigenous prisoners in Victoria, and implications for the correctional system. Int J Audiol. 2009;48(3):123-34.
365. Rasmussen MK, Donoghue DA, Sheehan NW. Suicide/self-harm-risk reducing effects of an Aboriginal art program for Aboriginal prisoners. Adv Ment Health. 2018;16(2):141.
366. Reekie JM, Levy MH, Richards AH, Wake CJ, Siddall DA, Beasley HM, et al. Trends in HIV, hepatitis B and hepatitis C prevalence among Australian prisoners - 2004, 2007, 2010. Med J Aust. 2014;200(5):277-80.
367. Reser JP. Australian Aboriginal suicide deaths in custody: Cultural context and cluster evidence. Aust Psychol. 1989;24(3):325-42.
368. Reutens S, Butler T, Hwang YIJ, Withall A. A comparison of older and younger offenders with delusional jealousy. Psychiatr Psychol Law. 2023;30(5):618-31.

369. Rice A, Thompson JA, Briffa K. Bladder and bowel symptoms following imprisonment in West Australian female prisons. Int J Prison Health. 2021.
370. Riches VC, Parmenter TR, Wiese M, Stancliffe RJ. Intellectual disability and mental illness in the NSW criminal justice system. Int J Law Psychiatry. 2006;29(5):386-96.
371. Richmond RL, Indig D, Butler TG, Wilhelm KA, Archer VA, Wodak AD. Smoking and other drug characteristics of Aboriginal and non-Aboriginal prisoners in Australia. J Addict. 2013;2013:516342.
372. Richmond R, Indig D, Butler T, Wilhelm K, Archer V, Wodak A. A randomized controlled trial of a smoking cessation intervention conducted among prisoners. Addiction. 2013;108(5):966-74.
373. Richmond RL, Wilhelm KA, Indig D, Butler TG, Archer VA, Wodak AD. Cardiovascular risk among Aboriginal and non-Aboriginal smoking male prisoners: Inequalities compared to the wider community. BMC Public Health. 2011;11:783.
374. Richters J, Butler T, Schneider K, Yap L, Kirkwood K, Grant L, et al. Consensual sex between men and sexual violence in Australian prisons. Arch Sex Behav. 2012;41(2):517-24.
375. Riddell S, Nielssen O, Butler T, Christie M, Starmer G. The relationship between amphetamine use, crime and psychiatric disorder among prisoners in New South Wales. Psychiatr Psychol Law. 2006;13(2):160-5.
376. Riley BJ, Smith D, Baigent MF. Mindfulness and acceptance-based group therapy: An uncontrolled pragmatic pre-post pilot study in a heterogeneous population of female prisoners. Int J Offender Ther Comp Criminol. 2019;63(15):2572-85.
377. Rogerson B, Jacups SP, Caltabiano N. Cannabis use, dependence and withdrawal in Indigenous male inmates. J Subst Use. 2016;21(1):65-71.
378. Rose A, Trounson JS, Louise S, Shepherd S, Ogloff JRP. Mental health, psychological distress, and coping in Australian cross-cultural prison populations. J Trauma Stress. 2020;33(5):794-803.
379. Rose A, Trounson J, Skues J, Daffern M, Shepherd SM, Pfeifer JE, et al. Psychological wellbeing, distress and coping in Australian Indigenous and multicultural prisoners: A mixed methods analysis. Psychiatr Psychol Law. 2019;26(6):886-903.
380. Ross J, Field C, Kaye S, Bowman J. Prevalence and correlates of low self-reported physical health status among prisoners in New South Wales, Australia. Int J Prison Health. 2019;15(2):192-206.
381. Russell NK, Yee Tan K, Pestell CF, Connor S, Fitzpatrick JP. Therapeutic recommendations in the youth justice system cohort diagnosed with Foetal Alcohol Spectrum Disorder. Youth Justice. 2023;23(1):6-28.
382. Ryan N, Ackerman J, Bond C, Ready J, Kinner SA. Prison life and prior social experiences: Understanding their importance for Indigenous peoples’ re-entry outcomes. Br J Criminol. 2019;59(1):188-208.
383. Ryan N, Ackerman J, Ready J, Kinner SA. Indigeneity, prisoner visitation and reincarceration in Australia: The association between visits in prison and reincarceration for Indigenous and non-Indigenous people. Br J Criminol. 2020;60(4):1056-79.
384. Sapkota D, Dennison S, Allen J, Gamble J, Williams C, Malope-Rwodzi N, et al. Navigating pregnancy and early motherhood in prison: A thematic analysis of mothers' experiences. Health Justice. 2022;10(32).
385. Sawyer MG, Guidolin M, Schulz K, McGinnes B, Baghurst P, Zubrick SR. Mental health problems among young people on remand: Has anything changed since 1989? Aust N Z J Public Health. 2010;34(6):594-7.
386. Sawyer MG, Guidolin M, Schulz KL, McGinnes B, Zubrick SR, Baghurst PA. The mental health and wellbeing of adolescents on remand in Australia. Aust N Z J Psychiatry. 2010;44(6):551-9.
387. Sazzad HMS, McCredie L, Treloar C, Lloyd AR, Lafferty L. Violence and hepatitis C transmission in prison—A modified social ecological model. PLoS One. 2020;15(12):e0243106.
388. Schilders MR, Ogloff JRP. Review of point-of-reception mental health screening outcomes in an Australian Prison. J Forens Psychiatry Psychol. 2014;25(4):480-94.
389. Schneider K, Richters J, Butler T, Yap L, Richards A, Grant L, et al. Psychological distress and experience of sexual and physical assault among Australian prisoners. Crim Behav Ment Health. 2011;21(5):333-49.
390. Schofield PW, Butler TG, Hollis SJ, Smith NE, Lee SJ, Kelso WM. Neuropsychiatric correlates of traumatic brain injury (TBI) among Australian prison entrants. Brain Inj. 2006;20(13):1409-18.
391. Schofield PW, Butler TG, Hollis SJ, Smith NE, Lee SJ, Kelso WM. Traumatic brain injury among Australian prisoners: Rates, recurrence and sequelae. Brain Inj. 2006;20(5):499-506.
392. Schofield P, Butler T, Hollis S, D'Este C. Are prisoners reliable survey respondents? A validation of self-reported traumatic brain injury (TBI) against hospital medical records. Brain Inj. 2011;25(1):74-82.

393. Seamark RW, Gaughwin M, Owen N, Liew C. HIV infection among male prisoners in South Australia, 1989 to 1994. Aust N Z J Public Health. 1997;21(6):572-6.
394. Selvey LA, Lush D, Mistry SA, Sheridan JW, Krause V, Passaris I, et al. Investigation of notifications of hepatitis C in 1994: The experience of three health departments. Aust N Z J Public Health. 1996;20(5):525-9.
395. Sheehan Y, Cunningham EB, Cochrane A, Byrne M, Brown T, McGrath C, et al. A 'one-stop-shop' point-of-care hepatitis C RNA testing intervention to enhance treatment uptake in a reception prison: The PIVOT study. J Hepatol. 2023.
396. Shepherd S, Spivak B, Borschmann R, Kinner SA, Hachtel H. Correlates of self-harm and suicide attempts in justice-involved young people. PLoS One. 2018;13(2):e0193172.
397. Shepherd SM, Ogloff JRP, Thomas SDM. Are Australian prisons meeting the needs of Indigenous offenders? Health Justice. 2016;4(13):Article 13.
398. Shepherd SM, Ogloff JRP, Paradies Y, Pfeifer J. Aboriginal prisoners with cognitive impairment: Is this the highest risk group? Trends Issues Crim Crim Justice. 2017(536).
399. Shepherd SM, Ogloff JRP, Shea D, Pfeifer JE, Paradies Y. Aboriginal prisoners and cognitive impairment: the impact of dual disadvantage on Social and Emotional Wellbeing. J Intellect Disabil Res. 2017;61(4):385-97.
400. Shepherd SM, Hazel Delgado R, Sivasubramaniam D, Paradies Y. Predictors of distress and the protective impact of cultural engagement for Indigenous prisoners. J Offender Rehabil. 2018;57(6):367.
401. Shepherd SM, Spivak B, Arabena K, Paradies Y. Identifying the prevalence and predictors of suicidal behaviours for Indigenous males in custody. BMC Public Health. 2018;18(1159).
402. Shinkfield AJ, Graffam J. Community reintegration of ex-prisoners: Type and degree of change in variables influencing successful reintegration. Int J Offender Ther Comp Criminol. 2009;53(1):29-42.
403. Shinkfield AJ, Graffam J. The relationship between emotional state and success in community reintegration for ex-prisoners. Int J Offender Ther Comp Criminol. 2010;54(3):346-60.
404. Silva D, Colvin L, Glauert R, Bower C. Contact with the juvenile justice system in children treated with stimulant medication for attention deficit hyperactivity disorder: A population study. Lancet Psychiatry. 2014;1(4):278-85.
405. Sindicich N, Mills KL, Barrett EL, Indig D, Sunjic S, Sannibale C, et al. Offenders as victims: Post-traumatic stress disorder and substance use disorder among male prisoners. J Forens Psychiatry Psychol. 2014;25(1):44-60.
406. Skov SJ, Miller P, Hateley W, Bastian IB, Davis J, Tait PW. Urinary diagnosis of gonorrhoea and Chlamydia in men in remote Aboriginal communities. Med J Aust. 1997;166(9):468-71.
407. Smirnov A, Kemp R, Ward J, Henderson S, Williams S, Abhilash D, et al. Patterns of drug dependence in a Queensland (Australia) sample of Indigenous and non-Indigenous people who inject drugs. Drug Alcohol Rev. 2016;35(5):611-9.
408. Smirnov A, Kemp R, Ward J, Henderson S, Williams S, Abhilash D, et al. Hepatitis C viral infection and imprisonment among Aboriginal and Torres Strait Islander and non-Indigenous people who inject drugs. Drug Alcohol Rev. 2018;37(7):831-6.
409. Snow PC, Woodward M, Mathis M, Powell MB. Language functioning, mental health and alexithymia in incarcerated young offenders. Int J Speech Lang Pathol. 2016;18(1):20-31.
410. Snow KJ, Richards AH, Kinner SA. Use of multiple data sources to estimate hepatitis C seroprevalence among prisoners: A retrospective cohort study. PLoS One. 2017;12(7):e0180646.
411. Snow KJ, Young JT, Preen DB, Lennox NG, Kinner SA. Incidence and correlates of hepatitis C virus infection in a large cohort of prisoners who have injected drugs. BMC Public Health. 2014;14:830.
412. Snow PC, Powell MB. Oral language competence in incarcerated young offenders: Links with offending severity. Int J Speech Lang Pathol. 2011;13(6):480-9.
413. Snow KJ, Petrie D, Young JT, Preen DB, Heffernan E, Kinner SA. Impact of dual diagnosis on healthcare and criminal justice costs after release from Queensland prisons: A prospective cohort study. Aust J Prim Health. 2022;28(3):264-70.
414. Sodhi-Berry N, Knuiman M, Alan J, Morgan VA, Preen DB. Pre- and post-sentence mental health service use by a population cohort of older offenders (>=45 years) in Western Australia. Soc Psychiatry Psychiatr Epidemiol. 2015;50(7):1097-110.
415. Sodhi-Berry N, Knuiman M, Alan J, Morgan VA, Preen DB. Pre-sentence mental health service use predicts post-sentence mortality in a population cohort of first-time adult offenders. Soc Psychiatry Psychiatr Epidemiol. 2015;50(1):109-24.
416. Sodhi-Berry N, Knuiman M, Preen DB, Alan J, Morgan VA. Predictors of post-sentence mental health service use in a population cohort of first-time adult offenders in Western Australia. Crim Behav Ment Health. 2015;25(5):355-74.

417. Spittal MJ, Forsyth S, Borschmann R, Young JT, Kinner SA. Modifiable risk factors for external cause mortality after release from prison: A nested case-control study. Epidemiol Psychiatr Sci. 2019;28(2):224-33.
418. Spittal MJ, Forsyth S, Pirkis J, Alati R, Kinner SA. Suicide in adults released from prison in Queensland, Australia: A cohort study. J Epidemiol Community Health. 2014;68(10):993-8.
419. Spivak B, Shepherd S, Borschmann R, Kinner SA, Ogloff JRP, Hachtel H. Crystalline methamphetamine (ice) use prior to youth detention: A forensic concern or a public health issue? PLoS One. 2020;15(5):e0229389.
420. Stathis SL, Doolan I, Letters P, Arnett A, Cory S, Quinlan L. Use of the Westerman Aboriginal symptoms checklist - youth (WASC-Y) to screen for mental health problems in Indigenous youth in custody. Adv Ment Health. 2012;10(3):235-9.
421. Stathis S, Letters P, Dacre E, Doolan I, Health K, Litchfield B. The role of an Indigenous Health Worker in contributing to equity of access to a mental health and substance abuse service for Indigenous young people in a youth detention centre. Aus Adv Ment Health. 2007;6(1):1-10.
422. Stathis S, Letters P, Doolan I, Fleming R, Heath K, Arnett A, et al. Use of the Massachusetts Youth Screening Instrument to assess mental health problems in young people within an Australian youth detention centre. J Paediatr Child Health. 2008;44(7):438-43.
423. Steele ML, Meurk C, Schess J, Yap L, Jones J, Harden S, et al. Substance use and help-seeking among justice-involved young people in Queensland and Western Australia: A cross-sectional survey of 14-17-year-olds. Drug Alcohol Rev. 2021;40(4):617-26.
424. Stewart A, Ogilvie JM, Thompson C, Dennison S, Allard T, Kisely S, et al. Lifetime prevalence of mental illness and incarceration: An analysis by gender and Indigenous status. Aust J Soc Issues. 2021;56(2):244-68.
425. Stewart AC, Cossar R, Walker S, Wilkinson AL, Quinn B, Dietze P, et al. Strategies to maximise study retention and limit attrition bias in a prospective cohort study of men reporting a history of injecting drug use released from prison: The prison and transition health study. BMC Med Res Methodol. 2021;21(1):Article 185.
426. Stewart AC, Cossar RD, Wilkinson AL, Quinn B, Dietze P, Walker S, et al. The prison and transition health (PATH) cohort study: Prevalence of health, social, and crime characteristics after release from prison for men reporting a history of injecting drug use in Victoria, Australia. Drug Alcohol Depend. 2021;227.
427. Stewart AC, Cossar R, Wilkinson AL, Scott N, Dietze P, Quinn B, et al. Psychiatric well-being among men leaving prison reporting a history of injecting drug use: A longitudinal analysis. Aust N Z J Psychiatry. 2022;56(8):1034-43.
428. Stewart LM, Henderson CJ, Hobbs MST, Ridout SC, Knuiman MW. Risk of death in prisoners after release from jail. Aust N Z J Public Health. 2004;28(1):32-6.
429. Stewart AC, Cossar R, Dietze P, Armstrong G, Curtis M, Kinner SA, et al. Lifetime prevalence and correlates of self-harm and suicide attempts among male prisoners with histories of injecting drug use. Health Justice. 2018;6(1):Article 19.
430. Stoové MA, Dietze PM, Aitken CK, Jolley D. Mortality among injecting drug users in Melbourne: A 16-year follow-up of the Victorian Injecting Cohort Study (VICS). Drug Alcohol Depend. 2008;96(3):281-5.
431. Strand S, Luebbers S, Shepherd SM. Psychopathic features in young incarcerated females. J Crim Psychol. 2016;6(2):63-75.
432. Sturman N, Saiepour N. Eligibility for opiate substitution therapy in recently released prisoners with high-risk amphetamine use, and their perceptions of its effectiveness. J Subst Use. 2017;22(5):484-9.
433. Sullivan RP, Baird R, Freeman K, Heggie H, Davis JS, Marshall CS, et al. Viral hepatitis in correctional facilities in the Northern Territory of Australia 2003–2017. BMC Infect Dis. 2021;21(1):Article 584.
434. Sullivan E, Zeki R, Ward S, Sherwood J, Remond M, Chang S, et al. Effects of the connections program on return-to-custody, mortality and treatment uptake among people with a history of opioid use: Retrospective cohort study in an Australian prison system. Addiction. 2024;119(1):169-79.
435. Sullivan EA, Kendall S, Chang S, Baldry E, Zeki R, Gilles M, et al. Aboriginal mothers in prison in Australia: A study of social, emotional and physical wellbeing. Aust N Z J Public Health. 2019;43(3):241-7.
436. Taflan P, Simpson PL, Wilson M, Jones J, Donovan B, Amin J, et al. Sexually transmissible infections (STI) and HIV testing and diagnosis among Aboriginal and non-Aboriginal adolescents in contact with the Australian justice system: A cross-sectional study. Sex Health. 2023;20(4):303-14.

437. Tambakis G, Schildkraut T, Delaney I, Gilmore R, Loebenstein M, Taylor A, et al. Management of foreign body ingestion in adults: Time to STOP and rethink endoscopy. Endosc Int Open. 2023;11(12):E1161-E7.
438. Tan GKY, Pestell CF, Fitzpatrick J, Cross D, Adams I, Symons M. Exploring offending characteristics of young people with foetal alcohol spectrum disorder in Western Australia. Psychiatr Psychol Law. 2023;30(4):514-35.
439. Tatkovic A, Moore K, Lim JC. Casemix and performance of Australian emergency departments: A comparison of major city, regional and remote locations. Emerg Med Australas. 2023.
440. Taylor M, Caffery LJ, Scuffham PA, Smith AC. Economic modelling of telehealth substitution of face-to-face specialist outpatient consultations for Queensland correctional facilities. Aust Health Rev. 2018;42(5):522-8.
441. Templeton DJ, Tyson BA, Meharg JP, Habgood KE, Bullen PM, Malek S, et al. Aboriginal health worker screening for sexually transmissible infections and blood-borne viruses in a rural Australian juvenile correctional facility. Sex Health. 2010;7(1):44-8.
442. Teutsch S, Luciani F, Scheuer N, McCredie L, Hosseiny P, Rawlinson W, et al. Incidence of primary hepatitis C infection and risk factors for transmission in an Australian prisoner cohort. BMC Public Health. 2010;10(633).
443. Thein HH, Butler T, Krahn M, Rawlinson W, Levy MH, Kaldor JM, et al. The effect of hepatitis C virus infection on health-related quality of life in prisoners. J Urban Health. 2006;83(2):275-88.
444. Thomas EG, Spittal MJ, Heffernan EB, Taxman FS, Alati R, Kinner SA. Trajectories of psychological distress after prison release: Implications for mental health service need in ex-prisoners. Psychol Med. 2016;46(3):611-21.
445. Thomas EG, Spittal MJ, Taxman FS, Kinner SA. Health-related factors predict return to custody in a large cohort of ex-prisoners: New approaches to predicting re-incarceration. Health Justice. 2015;3(1):Article 10.
446. Thomas EG, Spittal MJ, Taxman FS, Puljevic C, Heffernan EB, Kinner SA. Association between contact with mental health and substance use services and reincarceration after release from prison. PLoS One. 2022;17(9):e0272870.
447. Thomas E, Degenhardt L, Alati R, Kinner S. Predictive validity of the AUDIT for hazardous alcohol consumption in recently released prisoners. Drug Alcohol Depend. 2014;134:322-9.
448. Thompson SC, Ogilvie E, Veit F, Crofts N. Serostatus for vaccine-preventable diseases in residents at Melbourne Juvenile Justice Centre. Aust N Z J Public Health. 1998;22(5):573-7.
449. Thompson SC, Ogilvie EL, Veit FC, Crofts N. Juvenile offenders and hepatitis B: Risk, vaccine uptake and vaccination status. Med J Aust. 1998;169(6):306-9.
450. Treloar C, Idle J, Valentine K. Child sexual abuse, alcohol and other drug use and the criminal justice system: The meanings of trauma in survivor narratives for a national royal commission. Qual Health Res. 2023;33(1):117-26.
451. Treloar C, Jackson C, Gray R, Newl, Jamee, Wilson H, et al. Care and treatment of hepatitis C among Aboriginal people in New South Wales, Australia: Implications for the implementation of new treatments. Ethn Health. 2016;21(1):39-57.
452. Trofimovs J, Dowse L. Mental health at the intersections: The impact of complex needs on police contact and custody for Indigenous Australian men. Int J Law Psychiatry. 2014;37(4):390-8.
453. Trofimovs J, Dowse L, Srasuebkul P, Trollor JN. Impact of post-release community mental health and disability support on reincarceration for prisoners with intellectual disability and serious mental illness in NSW, Australia. BJPsych Open. 2023;9(2):e44.
454. Trofimovs J, Dowse L, Srasuebkul P, Trollor JN. Using linked administrative data to determine the prevalence of intellectual disability in adult prison in New South Wales, Australia. J Intellect Disabil Res. 2021;65(6):589-600.
455. Trofimovs J, Srasuebkul P, Trollor JN, Dowse L. Disability support and reincarceration after a first adult prison custody episode for people with intellectual disability in New South Wales, Australia. J Crim. 2022;55(2):239-59.
456. Trotter C, Baidawi S. Older prisoners: Challenges for inmates and prison management. Australian and New Zealand Journal of Criminology. 2015;48(2):200-18.
457. Trotter C, McIvor G, Sheehan R. The effectiveness of support and rehabilitation services for women offenders. Aust Soc Work. 2012;65(1):6-20.
458. Tye CS, Mullen PE. Mental disorders in female prisoners. Australian and New Zealand Journal of Psychiatry. 2006;40(3):266-71.
459. Valerio H, Alavi M, Conway A, Silk D, Treloar C, Martinello M, et al. Declining prevalence of current HCV infection and increased treatment uptake among people who inject drugs: The ETHOS engage study. Int J Drug Policy. 2022;105.

460. Valerio H, Alavi M, Marshall AD, Hajarizadeh B, Amin J, Law M, et al. Factors associated with hepatitis C treatment uptake among females of childbearing age in New South Wales, Australia: A population-based study. Drug Alcohol Rev. 2023:1-13.
461. Valerio H, Alavi M, Silk D, Treloar C, Martinello M, Milat A, et al. Progress Towards Elimination of Hepatitis C Infection Among People Who Inject Drugs in Australia: The ETHOS Engage Study. Clin Infect Dis. 2021;73(1):e69-e78.
462. van Beek I, Dwyer R, Dore GJ, Luo K, Kaldor JM. Infection with HIV and hepatitis C virus among injecting drug users in a prevention setting: Retrospective cohort study. Br Med J. 1998;317(7156):433-7.
463. van der Poorten D, Kenny DT, George J. Prevalence of and risk factors for hepatitis C in Aboriginal and non-Aboriginal adolescent offenders. Med J Aust. 2008;188(10):610-4.
464. van Dooren K, Claudio F, Kinner SA, Williams M. Beyond reintegration: A framework for understanding ex-prisoner health. Int J Prison Health. 2011;7(4):26-36.
465. van Dooren K, Kinner SA, Butler T. Young prisoners: An important group for health research? J Correct Health Care. 2010;16(4):322-7.
466. van Dooren K, Kinner SA, Forsyth S. Risk of death for young ex-prisoners in the year following release from adult prison. Aust N Z J Public Health. 2013;37(4):377-82.
467. van Dooren K, Kinner SA, Hellard M. A comparison of risk factors for hepatitis C among young and older adult prisoners. J Correct Health Care. 2014;20(4):280-91.
468. Walker A. Dissociation in incarcerated juvenile male offenders--A pilot study in Australia. Psychiatr Psychol Law. 2002;9(1):56-61.
469. Walker JR, Hilder L, Levy MH, Sullivan EA. Pregnancy, prison and perinatal outcomes in New South Wales, Australia: A retrospective cohort study using linked health data. BMC Pregnancy Childbirth. 2014;14:214.
470. Walker MR, Li H, Teutsch S, Betz-Stablein B, Luciani F, Lloyd AR, et al. Incident hepatitis C virus genotype distribution and multiple infection in Australian prisons. J Clin Microbiol. 2016;54(7):1855-61.
471. Walker S, Higgs P, Stoove M, Wilson M. Narratives of young men with injecting drug use histories leaving adult prison. Int J Offender Ther Comp Criminol. 2018;62(12):3681-707.
472. Walker S, Seear K, Higgs P, Stoove M, Wilson M. "A spray bottle and a lollipop stick": An examination of policy prohibiting sterile injecting equipment in prison and effects on young men with injecting drug use histories. Int J Drug Policy. 2020;80:102532.
473. Wallis C, O'Flynn M, Fenech M, Grimstrup D. Hepatitis C virus point-of-care RNA testing: Experience from screening an entire high-security Australian prison population over 3 days. Aust N Z J Public Health. 2023;47(5).
474. Walsh T. Women who die in custody: What Australian coroners' reports tell us. Howard J Crim Justice. 2022;61(4):540-55.
475. Walsh T, Counter A. Deaths in custody in Australia: A quantitative analysis of coroners' reports. Curr Issues Crim Justice. 2019;31(2):143-63.
476. Wand H, Iversen J, Wilson D, Topp L, Maher L. Developing and validating a scoring tool for identifying people who inject drugs at increased risk of hepatitis C virus infection. BMJ Open. 2012;2(1):e000387.
477. Wand H, Richmond R, Adily A, Le A, Wilhelm K, Butler T. Identifying significant contributors for smoking cessation among male prisoners in Australia: Results from a randomised clinical trial. BMJ Open. 2020;10(7):e034046.
478. Watkins RE, Mak DB, Connelly C. Testing for sexually transmitted infections and blood borne viruses on admission to Western Australian prisons. BMC Public Health. 2009;9(385).
479. Watson PG, Watts JR, Nelson M. The incidence of Australia antigen and antibody in male prisoners of two Sydney penitentiaries. Med J Aust. 1973;2(9):421-3.
480. White B, Dore GJ, Lloyd A, Rawlinson W, Maher L. Ongoing susceptibility to hepatitis B virus infection among people who inject drugs in Sydney. Aust N Z J Public Health. 2012;36(4):351-6.
481. White P, Chant D. The psychometric properties of a psychosis screen in a correctional setting. Int J Law Psychiatry. 2006;29(2):137-44.
482. White P, Chant D, Whiteford H. A comparison of Australian men with psychotic disorders remanded for criminal offences and a community group of psychotic men who have not offended. Aust N Z J Psychiatry. 2006;40(3):260-5.
483. White R, Boyer K. Alcoholism amongst the Tasmanian prison population: Research note. Aust N Z J Criminol. 1985;18(2):109.

484. Whiteman D, McCall B, Falconer A. Prevalence and determinants of hepatitis A virus exposure among prison entrants in Queensland, Australia: Implications for public health control. J Viral Hepat. 1998;5(4):277-83.
485. Willoughby M, Keen C, Young JT, Spittal MJ, Borschmann R, Janca E, et al. Violence-related morbidity among people released from prison in Australia: A data linkage study. Drug Alcohol Rev. 2021;41(2):457-66.
486. Willoughby M, Spittal MJ, Borschmann R, Tibble H, Kinner SA. Violence-related deaths among people released from prison: A data linkage study. J Interpers Violence. 2021;36(23):NP13229-NP53.
487. Willoughby M, Young JT, Borschmann R, Spittal MJ, Keen C, Hail-Jares K, et al. Violence-related death in young Australians after contact with the youth justice system: A data linkage study. J Interpers Violence. 2023:8862605231169490.
488. Willoughby M, Young JT, Hail-Jares K, Spittal MJ, Borschmann R, Patton G, et al. Circumstances and toxicology of violence-related deaths among young people who have had contact with the youth justice system: A data linkage study. BMC Public Health. 2021;21(2207).
489. Winter RJ, Dietze PM, Gouillou M, Hellard ME, Robinson P, Aitken CK. Hepatitis B virus exposure and vaccination in a cohort of people who inject drugs: What has been the impact of targeted free vaccination? J Gastroenterol Hepatol. 2013;28(2):314-22.
490. Winter RJ, Stoove M, Agius PA, Hellard ME, Kinner SA. Injecting drug use is an independent risk factor for reincarceration after release from prison: A prospective cohort study. Drug Alcohol Rev. 2019;38(3):254-63.
491. Winter RJ, Stoové M, Degenhardt L, Hellard ME, Spelman T, Jenkinson R, et al. Incidence and predictors of non-fatal drug overdose after release from prison among people who inject drugs in Queensland, Australia. Drug Alcohol Depend. 2015;153:43-9.
492. Winter RJ, White B, Kinner SA, Stoové M, Guy R, Hellard ME. A nurse-led intervention improved blood-borne virus testing and vaccination in Victorian prisons. Aust N Z J Public Health. 2016;40(6):592-4.
493. Winter RJ, Young JT, Stoove M, Agius PA, Hellard ME, Kinner SA. Resumption of injecting drug use following release from prison in Australia. Drug Alcohol Depend. 2016;168:104-11.
494. Wolk J, Wodak A, Morlet A, Guinan JJ, Gold J. HIV-related risk-taking behaviour, knowledge and serostatus of intravenous drug users in Sydney. Med J Aust. 1990;152(9):453-8.
495. Wu J, Boyle S, Cockburn T, Kelly AM. What can coronial cases tell us about the quality of emergency healthcare for prisoners in Australia? Emerg Med Australas. 2023;35(3):510-4.
496. Yap L, Butler T, Richters J, Kirkwood K, Grant L, Saxby M, et al. Do condoms cause rape and mayhem? The long-term effects of condoms in New South Wales' prisons. Sex Transm Infect. 2007;83(3):219-22.
497. Yap L, Butler T, Richters J, Malacova E, H., an, et al. Penile implants among prisoners-a cause for concern? PLoS One. 2013;8(1):e53065.
498. Yap L, Richters J, Butler T, Schneider K, Grant L, Donovan B. The decline in sexual assaults in men's prisons in New South Wales: A "systems" approach. J Interpers Violence. 2011;26(15):3157-81.
499. Yee N, Chemjong P, Korobanova D, Scade S, Large M, Nielssen O, et al. The full spectrum of clinical stages of psychosis among mentally ill prisoners in New South Wales (NSW), Australia. Psychiatr Psychol Law. 2022.
500. Young JT, Arnold-Reed D, Preen D, Bulsara M, Lennox N, Kinner SA. Early primary care physician contact and health service utilisation in a large sample of recently released ex-prisoners in Australia: Prospective cohort study. BMJ Open. 2015;5(6):e008021.
501. Young JT, Borschmann R, Heffernan E, Spittal MJ, Brophy L, Ogloff JRP, et al. Contact with mental health services after acute care for self-harm among adults released from prison: A prospective data linkage study. Suicide Life Threat Behav. 2020;50(5):990-1006.
502. Young JT, Borschmann R, Preen DB, Spittal MJ, Brophy L, Wang EA, et al. Age-specific incidence of injury-related hospital contact after release from prison: A prospective data-linkage study. Inj Prev. 2020;26(3):204-14.
503. Young JT, Cumming C, van Dooren K, Lennox NG, Alati R, Spittal MJ, et al. Intellectual disability and patient activation after release from prison: A prospective cohort study. J Intellect Disabil Res. 2017;61(10):939-56.
504. Young JT, Heffernan E, Borschmann R, Ogloff JRP, Spittal MJ, Kouyoumdjian FG, et al. Dual diagnosis of mental illness and substance use disorder and injury in adults recently released from prison: A prospective cohort study. Lancet Public Health. 2018;3(5):e237-e48.
505. Young JT, van Dooren K, Lennox NG, Butler TG, Kinner SA. Inter-rater reliability of the Hayes Ability Screening Index in a sample of Australian prisoners. J Intellect Disabil Res. 2015;59(11):1055-60.

506. Young LC, Dwyer DE, Harris M, Guse Z, Noel V, Levy MH. Summer outbreak of respiratory disease in an Australian prison due to an influenza A/Fujian/411/2002(H3N2)-like virus. Epidemiol Infect. 2005;133(1):107-12.
507. Young M, Waters B, Falconer T, O'Rourke P. Opportunities for health promotion in the Queensland women's prison system. Aust N Z J Public Health. 2005;29(4):324-7.
508. Yousafzai MT, Alavi M, Valerio H, Hajarizadeh B, Grebely J, Dore GJ. Hepatitis C care cascade before and during the direct-acting antiviral eras in New South Wales, Australia: A population-based linkage study. J Viral Hepat. 2022;30(3):250-61.
509. Zilkens RR, Smith DA, Mukhtar SA, Semmens JB, Phillips MA, Kelly MC. Male sexual assault: Physical injury and vulnerability in 103 presentations. J Forensic Leg Med. 2018;58:145-51.
510. Butler T, Milner L. The 2001 New South Wales inmate health survey. Sydney (AU): Corrections Health Service, 2003.
511. Butler TG. Preliminary findings from the inmate health survey of the inmate population in the NSW correctional system. Sydney (AU): Corrections Health Service; 1997.
512. Indig D, Topp L, Ross B, Mamoon H, Border B, Kumar S, et al. 2009 NSW inmate health survey: Key findings report. Sydney (AU): Justice Health, 2010.
513. Indig D, Vecchiato C, Haysom L, Beilby R, Carter J, Champion U, et al. 2009 NSW young people in custody health survey: Full report. Internet. Sydney (AU): Justice Health and Juvenile Justice; 2011. Available from: https://www.nsw.gov.au/sites/default/files/2022-05/2009_NSW_YPiCHS_Full_report.pdf.
514. Allerton M, Champion U, Beilby R, Butler T, Fasher M, Kenny D, et al. 2003 NSW young people in custody health survey. Sydney (AU): NSW Department of Juvenile Justice; 2003 December. Available from: https://www.nsw.gov.au/sites/default/files/2022-05/2003_NSW_YPICHS_Key_findings_report.pdf.
